# Chick tendon fibroblast transcriptome and shape depend on whether the cell has made its own collagen matrix

**DOI:** 10.1038/srep13555

**Published:** 2015-09-04

**Authors:** Ching-Yan Chloé Yeung, Leo A. H. Zeef, Chloe Lallyett, Yinhui Lu, Elizabeth G. Canty-Laird, Karl E. Kadler

**Affiliations:** 1 Wellcome Trust Centre for Cell-Matrix Research, Oxford Road, Manchester M13 9PT United Kingdom; 2 Faculty of Life Sciences, University of Manchester, Michael Smith Building, Oxford Road, Manchester M13 9PT United Kingdom; 3 Department of Musculoskeletal Biology, Institute of Ageing and Chronic Disease, Faculty of Health and Life Sciences, University of Liverpool, United Kingdom; 4 The MRC-Arthritis Research UK Centre for Integrated research into Musculoskeletal Ageing (CIMA), United Kingdom

## Abstract

Collagen- and fibrin-based gels are extensively used to study cell behaviour. However, 2D–3D and collagen-fibrin comparisons of gene expression, cell shape and mechanotransduction, with an *in vivo* reference, have not been reported. Here we compared chick tendon fibroblasts (CTFs) at three stages of embryonic development with CTFs cultured in collagen- or fibrin-based tissue engineered constructs (TECs). CTFs synthesised their own collagen matrix in fibrin-based TECs and better recapitulated the gene expression, collagen fibril alignment and cell shape seen *in vivo*. In contrast, cells in 3D collagen gels exhibited a 2D-like morphology and expressed fewer of the genes expressed *in vivo*. Analysis of YAP/TAZ target genes showed that collagen gels desensitise mechanotransduction pathways. In conclusion, gene expression and cell shape are similar on plastic and 3D collagen whereas cells in 3D fibrin have a shape and transcriptome better resembling the *in vivo* situation. Implications for wound healing are discussed.

Three-dimensional (3D) gels made from collagen-I are widely used in studies of cell behaviour. This is because collagen-I is the most abundant structural protein in vertebrates, it contains ligands for cell attachment, is cheap to obtain in large quantities from animal tissues, and does not require specialised machinery to assemble into firm gels. The high sequence identity of collagen-I across a wide range of species means that fibrils formed from human, rat, mouse, bovine or chick collagen-I have the same ‘cell interaction domains’ and ‘matrix interaction domains’^1^,^2^, which greatly facilitates cross-study comparisons. A comprehensive list of studies using collagen gels is outside the scope of this article but a few recent examples include: cell migration^3^,^4^, cell signalling^5^,^6^,^7^, cell differentiation^8^,^9^, chondrogenesis and osteogenesis^10^, tendon formation^11^, mechanotransduction^12^, and wound healing^13^,^14^. Another popular scaffold system is fibrin (formed by cleavage of fibrinogen with thrombin)^15^,^16^,^17^ sometimes in combination with collagen and hyaluronan, e.g. in studies of wound healing^18^,^19^. A wide variety of cells have been cultured in 3D collagen and fibrin gels, of which the most popular are fibroblasts. Despite the popularity of 3D gels for cell culture, a comprehensive comparison of gene expression of fibroblasts *in vivo* versus fibroblasts in 3D collagen gels or 3D fibrin gels is not available. In addition, whereas fibroblasts *in vivo* are typically surrounded by a tensioned collagenous matrix, a comparison of gene expression in tensioned and untensioned gels has not been reported.

In this study we used embryonic chick tendon fibroblasts (CTFs) because they are highly motile in 2D culture, proliferate well, and can be isolated by clean dissection from chick embryos without the need for extensive handling procedures. Moreover, tendons can be dissected from chick embryos at embryonic day 11 (E11) through to hatching (E21), thereby allowing comparative studies of fibroblasts during tendon development. We used collagen and fibrin to prepare tissue engineered constructs (TECs) according to procedures previously described^17^. The TECs began as discs of gel within a plastic culture dish that contained pinned silk sutures positioned 10 mm apart. The TECs were either left attached to the rim (restrained) or detached, which triggered cell-induced contraction to a linear TEC. A critical difference between the collagen-based and fibrin-based TECs is that the collagen matrix in the fibrin-based TECs has been synthesised exclusively by the cells^15^.

## Results

### RNA sources for embryonic tendon tissue and TECs

**Figure 1:**
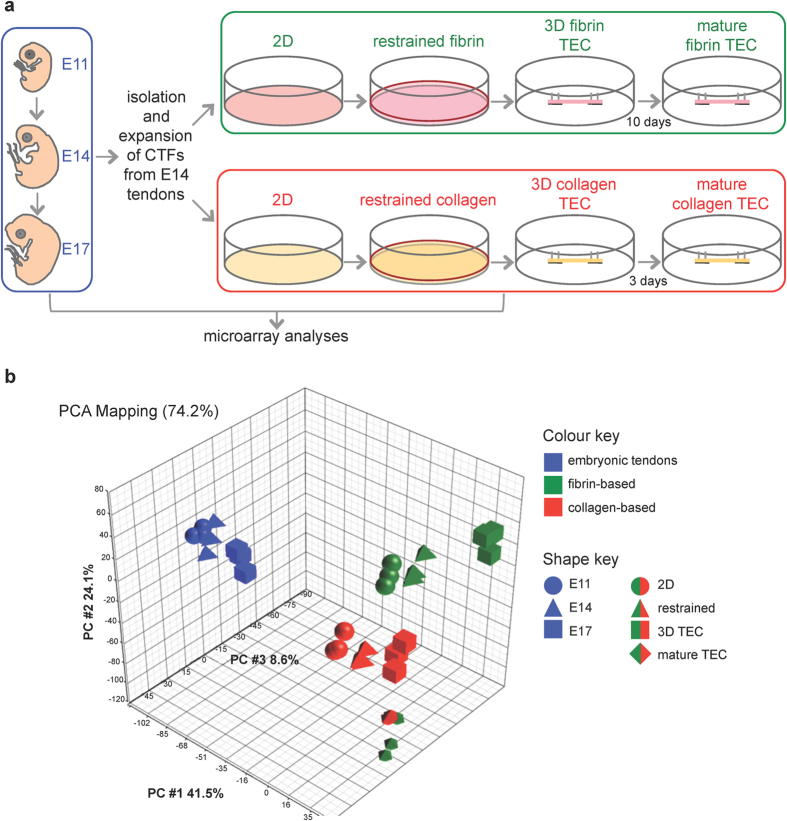
Experimental setup to compare transcriptomes of CTFs *in vivo* and in culture. (**a**) RNA was isolated from embryonic tendon at three stages of developmental – E11, E14 and E17. CTFs were isolated from E14 tendons and expanded in culture and subsequently seeded into fibrin or collagen gels and RNA was isolated from sequential steps of the culture model; 2D, 24 hours after being seeded into the gels (restrained), once a taut linear 3D TEC is formed and matured TEC (10 days for 3D fibrin TEC and 3 days for 3D collagen TEC). (**b**) Principal component analysis (PCA) was used to provide a statistical summary of the array samples, and the first three principle components are shown. See Supplementary Figure 1 for analysis of RNA and microarray readout quality.

Our first experiment was to identify genes expressed during tendon development *in vivo* and then determine which of these genes are expressed by CTFs cultured in collagen-based and fibrin-based TECs. A schematic showing the RNA samples used for microarray analyses is shown in Fig. 1a. Yield and quality of RNA isolated from triplicates of each group were assessed by gel electrophoresis (Supplementary Fig. 1a–c). RNA was isolated from the metatarsal toe tendons of chick embryos at E11, E14 and E17, during which time toe length more than doubles^20^ and tendon mechanical properties improve accordingly^21^. Primary cultures of CTFs were derived from E14 embryos and expanded in culture prior to transfer to either collagen or fibrin gels. RNA was isolated from CTFs cultured on 2D tissue culture plastic (2D), in restrained, attached fibrin gels (restrained fibrin), in taut tendon-like TECs (3D fibrin TEC), and in these fibrin-based TECs matured for 10 days in culture under tension (mature fibrin TEC)^15^. The 3D fibrin TEC consisted of two lengths of suture (10 mm apart secured to a base of silicone), between which the tendon-like construct formed^15^. Importantly, CTFs replace the fibrin with collagen by the time the TECs are fully-contracted and taut^15^. CTFs within 3D fibrin TECs exhibit contractile actin stress fibres aligned parallel to the alignment of the TEC, which are susceptible to ROCK inhibitor Y27632^22^, a mechanism similar to remodelling of collagen gels embedded with fibroblasts^23^.

RNA was also isolated from CTFs on 2D tissue culture plastic, CTFs in restrained, attached collagen gels (restrained collagen), from contracted collagen-based TECs (3D collagen TEC) and 3D collagen TECs matured for 3 days (mature collagen TEC). The 3D collagen TECs were formed similarly to 3D fibrin TECs in which two lengths of suture pinned 10 mm apart provided an interface between the collagen gel and the fixed minutien pins. 3D collagen constructs could only be cultured for up to 3 to 4 days post-contraction because they tended to detach from the fixed sutures.

### Global patterns of gene expression

Principal components analysis (PCA) was used to pick out patterns of gene expression in a dataset^24^. Quality control of the arrays prior to the PCA indicated a single technical outlier, which was excluded (see box plots in Supplementary Fig. 1d). PCA of the expression data from the remaining arrays clustered the samples according to their origin: embryonic tendon, fibrin gels and collagen gels, with the exception being the mature TECs (Fig. 1b). The first principal component (PC #1), accounting for the main pattern of gene expression, separates embryonic tendons in a tight group from the cultured cells. PC #2, accounting for the next most important pattern of gene expression, separates collagen and fibrin, with embryonic tendons more similar to CTFs in fibrin-based culture. However, with increasing time in fibrin or collagen (i.e. the mature TECs) the array data deviated further from the *in vivo* samples.

### Gene expression changes during 6 days of tendon development

We identified 2090 probe sets with significant differential gene expression between E11 and E14, E14 and E17, and E11 and E17 of tendon development, based on ≥±2-fold change (*q* < 0.05). Application of a clustering algorithm (as described in Material and Methods) produced 8 clusters based on similarity of expression profile across E11 to E17 and the expression of the genes within these clusters were compared to fibrin- and collagen-based TECs (see Supplementary Fig. 2a). Three of the eight clusters contained genes that exhibited similar expression trends during the formation of both fibrin- and collagen-based TECs and *in vivo* tendon development (Supplementary Fig. 2b). The DAVID online tool was used to perform gene ontology (GO) enrichment analysis on these three clusters and revealed that ‘lipid biosynthetic process’ and ‘microtubule cytoskeleton’ were similarly downregulated, whereas ‘extracellular region’ and ‘intermediate filament cytoskeleton’ were similarly upregulated during TEC formation and tendon development (Supplementary Fig. 2c). These data indicated that some elements of *in vivo* tendon development are recapitulated *in vitro*.

**Figure 2:**
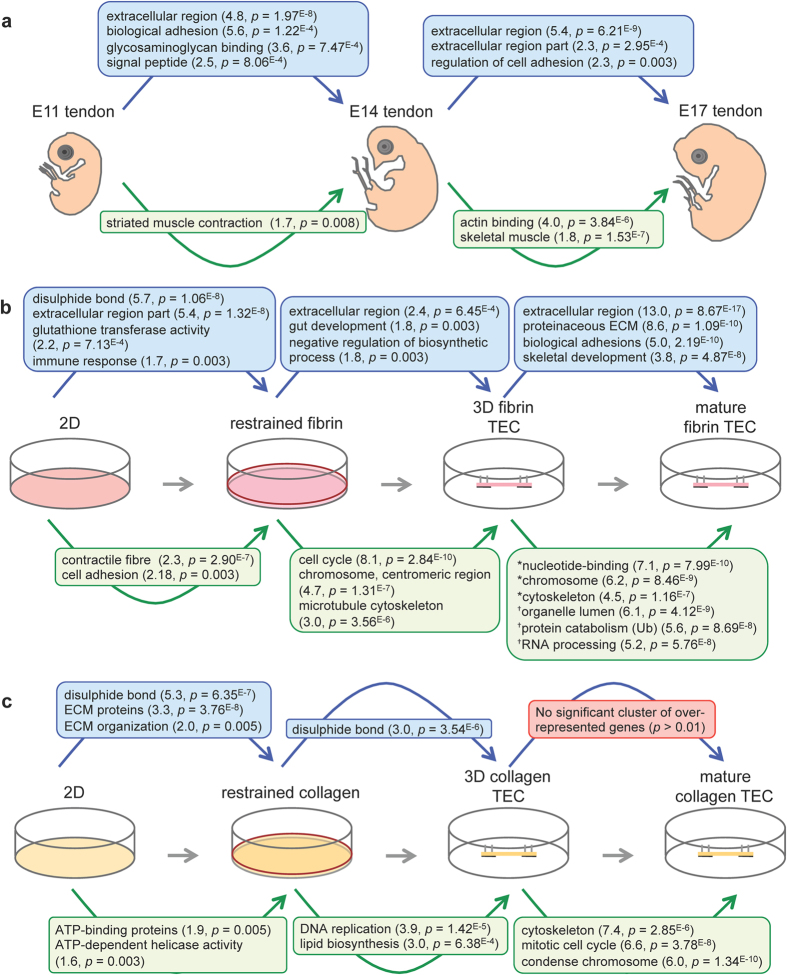
Gene ontology enrichment analysis of the differentially regulated genes tendon development and formation of 3D TECs. Significantly enriched GO terms identified in differentially expressed genes (≥±2-fold, *q* < 0.001; shown for highly significant GO terms with enrichment scores ≥1.5, *p* ≤ 0.008 as indicated in brackets) in stages of embryonic tendon development *in vivo* (**a**), and during formation of fibrin-based TECs (**b**) and collagen-based TECs (**c**). GO terms of upregulated genes are displayed in blue boxes, GO terms of downregulated genes are in green boxes. *GO terms derived from genes with a ≥2-3-fold change, ^†^GO terms derived from genes with a ≥3-fold change. See Supplementary Tables 1–3 for lists of genes of each GO term.

GO analysis of differentially expressed genes between the three stages of tendon development was performed to elucidate the biological processes that are over-represented (summarised in Fig. 2a; see Supplementary Table 1 for a list of the top annotation clusters with the highest enrichment scores (≥1.5) and the genes therein). Comparison between E11 and E14 tendons showed that 459 probe sets detected a significant difference ≥±2-fold (*q* < 0.001), of which 289 probe sets were upregulated and 170 probe sets were downregulated in E14 compared to E11. The top GO terms over-represented by the upregulated genes included ‘extracellular region’ and ‘biological adhesion’. The top GO term over-represented by the downregulated genes from E11 to E14 was ‘striated muscle contraction’.

Comparison between E14 and E17 tendons identified 656 probe sets with significantly different expression between the two stages of development (≥±2-fold, *q* < 0.001): 329 probe sets were upregulated and 327 were downregulated, from E14 to E17. The top GO terms of the upregulated genes were ‘extracellular region’, ‘extracellular region part’ and ‘regulation of cell adhesion’. The two GO terms over-represented by the downregulated genes were ‘actin binding’ and ‘skeletal’. Together, the GO analysis showed that as embryonic tendon developed there was a step-wise induction of specific ECM proteins and a dampening of genes regulating actin-mediated contractility and muscle specification.

### Gene expression changes during formation of fibrin-based TECs

GO analyses of the different phases of fibrin-based 3D TEC formation are summarised in Fig. 2b (see Supplementary Table 2 for a list of the top annotation clusters and the genes therein). The top three GO terms over-represented by genes that were upregulated from 2D to restrained fibrin gels (559 probe sets; *q* < 0.001) were ‘disulphide bond’, ‘extracellular region part’, ‘glutathione transferase activity’ and ‘immune response’. These gene lists included ECM structural proteins, ECM remodelling proteins, glutathione transferases and cytokines. Transfer of CTFs from 2D to restrained fibrin gels caused a significant downregulation of 614 probe sets. GO analysis revealed that the top clusters were ‘contractile fibre’ and ‘cell adhesion’. The downregulation of ‘contractile actin’ was similar to that observed during tendon development from E11 to E17.

CTFs in restrained fibrin gels were compared to fully-formed 3D fibrin-based TECs. Over 1600 probe sets detected significant changes (≥±2-fold change, *q* < 0.001), of which 821 were upregulated and 863 were downregulated. The top GO terms of upregulated genes were ‘extracellular region’, ‘gut development’ and ‘negative regulation of biosynthetic process’. Top GO terms clusters of the downregulated genes were ‘cell cycle’, ‘chromosome centromeric region’ and ‘microtubule cytoskeleton’.

Strikingly, comparing 3D fibrin-based TECs to matured fibrin TECs revealed that over 7000 probe sets detected differential regulation (≥±2-fold change, *q* < 0.001), over 5 times as many as was revealed when 2D and restrained fibrin gels were compared. Of these, 1758 probe sets were ≥+2-fold upregulated, 3213 were ≥−2<−3-fold downregulated and 2066 were ≥−3-fold downregulated (see Supplementary Table 2 for the top annotation clusters). The top GO terms for upregulated genes were ‘extracellular region’, ‘proteinaceous extracellular matrix’, ‘biological adhesion’ and ‘skeletal system development’. Within these top clusters there was a conspicuous number of genes encoding ECM structural proteins, ECM modifying enzymes, soluble growth factors, cadherins and skeletal development regulators. The upregulation in ECM structural proteins and cadherin expression during the maturation of fibrin-based TECs mirrors the GO terms identified during E11 to E14 of tendon development. Top GO terms for genes that were moderately downregulated (≥−2<−3-fold) during maturation of 3D fibrin TECs were ‘organelle lumen’, ‘modification-dependent macromolecule catabolic process’, many of which were mediators of ubiquitination, and ‘RNA processing’. Top GO terms of genes that were downregulated ≥−3-fold during maturation of 3D tendon constructs were ‘nucleotide-binding’, ‘chromosome’ and ‘cytoskeleton’, and contained gene profiles similar to that for clusters of downregulated genes from restrained fibrin to 3D fibrin TECs, which might suggest that the regulation of these genes are associated with tension and development of mechanical properties.

### Gene expression changes during formation of collagen-based TECs

GO analyses of the different phases of collagen-based TEC formation are summarised in Fig. 2c (see Supplementary Table 3 for a list of the top annotation clusters and the genes therein). A modest number (694) of probe sets displayed significant differential expression in CTFs cultured in 2D to restrained collagen gels (≥±2-fold change, *q* < 0.001), which suggests that embedment in collagen gels has a minor effect on the 2D phenotype of CTFs. The top three GO terms (‘disulphide bond’, ‘extracellular region’, ‘extracellular matrix organisation’) of upregulated genes in CTFs from 2D to restrained collagen gels were similar to those identified during the culture of fibrin-based TECs. The genes of these clusters were secreted growth factors, ECM structural proteins and ECM remodellers. However, analysis of the 302 probe sets that detected downregulation from 2D to restrained collagen gel were very different to the genes identified during tendon development *in vivo* and the formation of fibrin-based TECs. The GO terms of these downregulated genes were ‘ATP-binding’ and ‘ATP-dependent helicase activity’.

From untensioned restrained collagen gels to 3D collagen TECs, there were only 234 differentially regulated probe sets (≥±2-fold change, *q* < 0.001), further suggesting that CTFs in collagen gels are unable to detect differences in the physical/mechanical environment (i.e. 2D to 3D, untensioned to tensioned) and could explain why the microarray samples from collagen-based culture system were clearly grouped away from all the other groups in the PCA (Fig. 1b). The top GO term of the upregulated genes (detected by 43 probe sets) was ‘disulphide bond’ with very few ECM structural proteins (see Supplementary Table 3). The 191 probe sets that detected downregulation were clustered into the GO terms ‘DNA replication’ and ‘lipid biosynthetic process’.

The maturation of 3D collagen TECs for 3 days in culture induced differential gene expression detected by over 976 probe sets. However, only 58 probe sets detected upregulation and it was not possible to detect any enrichment in GO terms, suggesting that CTFs in collagen-based TECs are not particularly synthetic or that they are in homeostasis or undergoing senescence. The GO terms of downregulated genes were very similar to those identified during tensioning of fibrin-based TECs (restrained to 3D and 3D to mature), which included ‘cytoskeleton’ and ‘condensed chromosome’.

### Genes upregulated during tendon development are better represented in fibrin-based than collagen-based TECs

**Figure 3:**
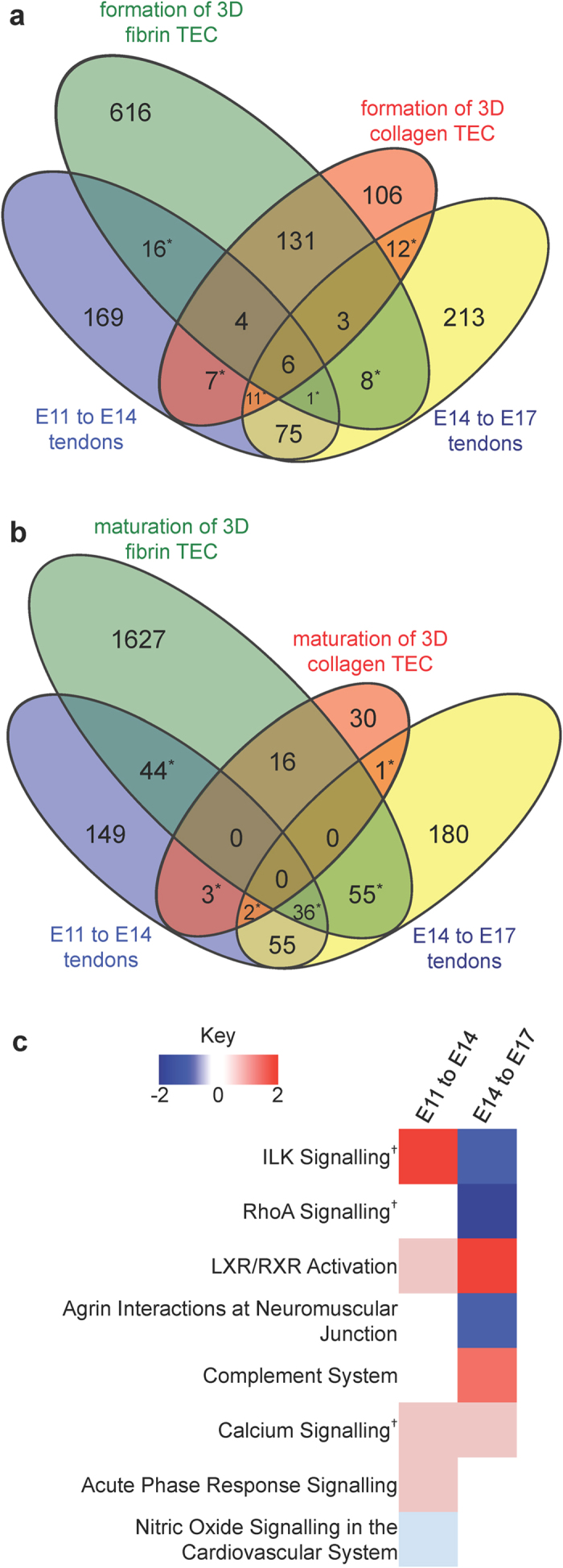
Comparison of similar genes upregulated during tendon development and formation of 3D TECs. Venn diagrams showing the number of common genes upregulated during tendon development from E11 to E14 and from E14 to E17 in comparison with formation of 3D TECs (**a**) and with maturation of 3D TECs (**b**). *Lists of genes in are available in Supplementary Table 4. (**c**) IPA-generated heat map comparison of pathways containing differentially regulated genes during E11 to E14 and during E14 to E17. Blue is downregulated, red is upregulated. ^†^Molecular networks of these pathways are available in Supplementary Figures 4 and 5.

The top GO terms over-represented by genes upregulated during the formation and maturation of both fibrin- and collagen-based TECs appeared to be very similar to tendon development. However, the GO terms’ gene lists suggested that tendon development *in vivo* was better recapitulated in formation and maturation of fibrin-based TECs than in the collagen-based system. To analyse this similarity more closely we filtered out genes that were significantly upregulated during the formation (restrained to 3D) or maturation (3D to mature) of TECs and made a comparison with E11 to E14 and E14 to E17 tendon development (≥2-fold, *q* < 0.001; see Fig. 3a,b; see Supplementary Table 4 for the gene lists of the highlighted comparisons). Of note, there were more uncommon than common genes in fibrin-based and collagen-based TECs when compared with *in vivo* tendon development. During the formation (restrained to 3D) of TECs there was greatest similarity between collagen-based TECs and tendon development *in vivo* (Fig. 3a). However, comparison of 3D TEC maturation under tension and tendon development *in vivo* showed that there were a large number of common genes in maturation of fibrin-based TECs than in maturation of collagen-based TECs (Fig. 3b). These data indicate that the variation between mature fibrin-based TECs and tendon *in vivo* identified in the first PCA (Fig. 1b) was probably due to the large number (>5000) of genes downregulated during fibrin TEC maturation.

To investigate further how the maturation of fibrin-based TECs better replicated tendon development, we performed a second PCA based on the 2090 probe sets differentially regulated during tendon development. The results showed that expression of these genes in mature 3D fibrin-based TECs was most similar to E17 tendons as distinguished by PC #2 which accounted for 18% of gene expression variance in the samples (see Supplementary Fig. 3). Pathway analysis was carried out to distinguish the processes that occur between E11 to E14 and E14 to E17 of tendon development. As expected, many of the 52 canonical pathways identified were common in both developmental transitions (Fig. 3c and Supplementary Table 5). One of the pathways that was similarly regulated during E11 to E14 and during E14 to E17 was ‘calcium signalling’ (see Supplementary Fig. 4). The two pathways that were regulated most differently during E11 to E14 compared with during E14 to E17 were ‘integrin-linked kinase signalling’ which upregulated in early tendon development and downregulated in late tendon development (Supplementary Fig. 5a and b), and ‘RhoA signalling’ which was downregulated from E14 to E17 (Supplementary Fig. 5c and d). The pathways regulated most differently between the *in vivo* developmental stages suggest that mechanotransduction in fibrin-based TECs may be an important process in mimicking tendon development.

### Ultrastructural differences between fibrin-based and collagen-based TECs

**Figure 4:**
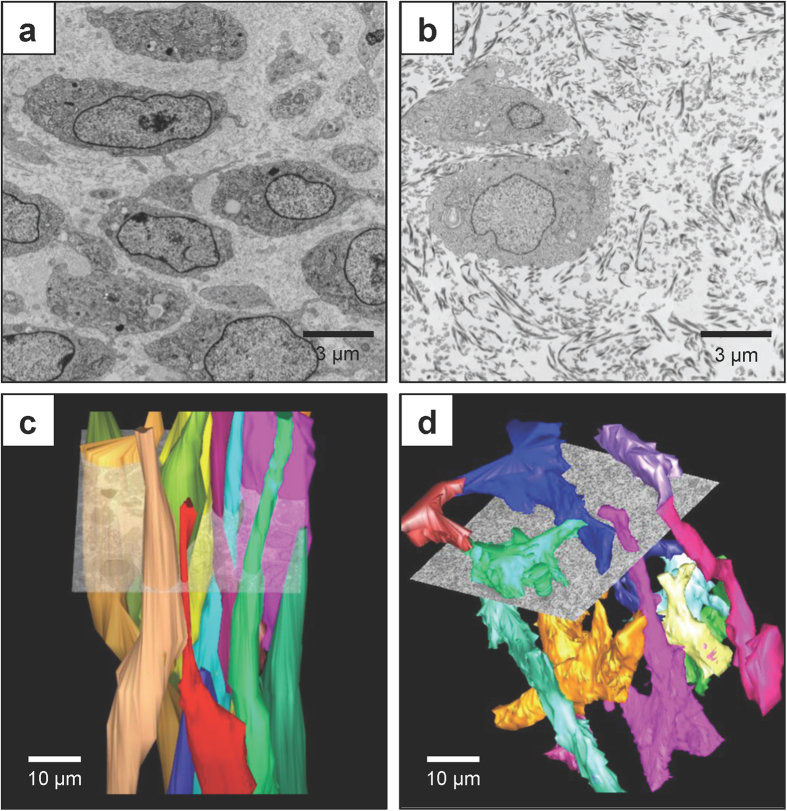
Ultrastructure of fibrin- and collagen-based 3D TECs. Images of transmission electron microscopy of transverse section across a 3D fibrin-based TEC (**a**) and a 3D collagen-based TEC (**b**). Scale bars 3 μm. 3D reconstruction of SBF-SEM of CTFs in 3D fibrin-based TECs (**c**) and 3D **c**ollagen-based TECs (**d**). Scale bars 10 μm. See Supplementary Figures 6 and 7 for a higher magnification images of 3D fibrin-based and collagen gel-based TECs. See Supplementary Movies 1 and 2 for step-through movies of 3D reconstruction of SBF-SEM of 3D fibrin-based and collagen-based TECs.

We prepared collagen-based and fibrin-based TECs for electron microscopy (EM), as previously described^25^. As expected, transmission EM showed the cells separated by extracellular collagen fibrils. The fibrin-based TECs contained narrow diameter (~35 nm) collagen fibrils that were, to a close approximation, aligned parallel to the long axis of the TECs (Fig. 4a and Supplementary Fig. 6). In contrast, the collagen-based TECs contained collagen fibrils that were irregular in diameter and could be randomly oriented (Fig. 4b). Closer inspection of the images of the collagen-based TECs showed the presence of narrow diameter (~35 nm) collagen fibrils that were always in close association with cell (some in fibripositors^26^) and aligned parallel to the long axis of the TECs (see Supplementary Fig. 7). Therefore, the collagen fibrils in the fibrin-based TECs had been synthesised exclusively by the CTFs whereas in the collagen-based TECs some of the collagen fibrils were from the pre-existing gel and some were newly-synthesised.

Serial block face-scanning electron microscopy (SBF-SEM) has emerged as a powerful tool with which to study the ultrastructure of developing tissues (for review, see^27^). Therefore, we used SBF-SEM to determine the shape of CTFs in collagen- and fibrin-based TECs. The results showed that CTFs in fibrin-based TECs were ellipsoid and aligned parallel to the long axis of the TEC, as previously observed in fibrin-based gels and *in vivo*^28^,^29^ (see Fig. 4c and Supplementary Movie 1). In contrast, CTFs in collagen-based TECs had numerous cytoplasmic projections with no particular orientation with respect to the long axis of the TEC (see Fig. 4d and Supplementary Movie 2). The CTFs in collagen-based TECs appeared to be stretched in random directions as the cells adhered to pre-existing collagen fibrils. The shape of the cell in 3D was similar to the stellate shape observed in 2D^30^.

### Collagen and 2D culture have similar mechanotransduction properties

**Figure 5:**
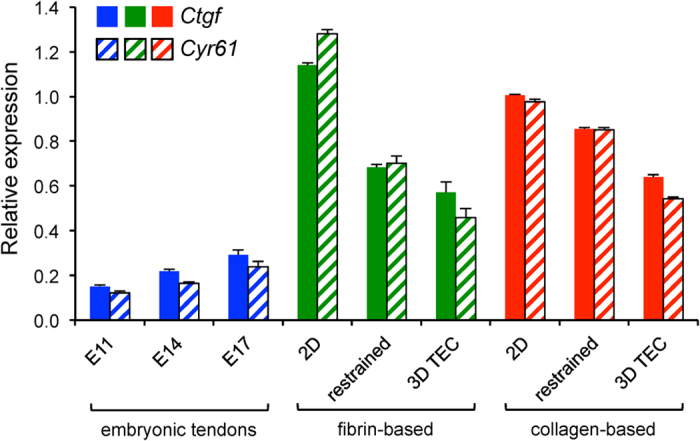
Expression of YAP/TAZ target genes in tendons *in vivo* and CTFs in culture. Expression levels of TAP/TAZ target genes *Ctgf* and *Cyr61* from the microarray data were normalised relative to the expression level in CTFs culture in 2D. Bars show SEM.

With regard to cell shape, we hypothesised that the strong adhesion of CTFs to collagen and the random arrangement of fibrils in the gels prevented cells from aligning with the TEC long axis. As a consequence, CTFs in collagen-based TECs adopted a shape that was in-between 2D and 3D. We normalised the microarray expression data of two bona fida target genes of the YAP/TAZ mechanosensing pathway^31^, *Ctgf* and *Cyr61*^32^ to levels expressed in 2D and compared them with *in vivo* expression levels. There was no obvious difference in expression levels of *Yap1* detected by two probe sets in the microarrays (see Supplementary Fig. 8). Compared to all the *in vitro* samples, the expression of *Ctgf* and *Cyr61* genes were halved in embryonic tendons. CTFs in 2D culture had the highest *Ctgf* and *Cyr61* expression, which was reduced 40–50% in fibrin-based TECs compared with a 10–20% reduction in collagen-based TECs (see Fig. 5). Interestingly, during tendon development *in vivo* YAP/TAZ target gene expression increases ~2-folds from E11 to E17, possibly reflecting the development of ECM mechanical properties.

## Discussion

We compared and contrasted the transcriptomes of CTFs in 2D, in tendon *in vivo* and in fibrin gel- and collagen gel-based tendon-like TECs. Based on the results of functional enrichment analysis and ultrastructural analysis, we conclude that neither collagen-based nor fibrin-derived TECs faithfully copy the changes in gene expression that occur *in vivo*. Although not studied here, it is likely that differences in cytokine profiles that the cells are exposed to *in vivo* versus in culture, are likely to explain some of these differences. However, the differences between *in vivo* and *in vitro* were less pronounced in fibrin-based gels. The upregulation and downregulation of genes that occur during embryonic tendon development was better reproduced in the formation of fibrin-based TECs. In particular, maturation of fibrin-based TECs under tension in culture mimicked a late aspect of tendon development. Collagen gels did not provide the environment to recapitulate the 3D cell morphology and ECM organisation seen in tendons *in vivo* or to reduce YAP/TAZ signalling to *in vivo* levels. Although the mechanical properties of tissue culture plastic and 3D collagen gels are very different, the response of the cell in terms of shape and transcriptome are remarkably similar. Presumably the opportunity for the cell to interact with collagen fibrils in all directions and the high affinity of CTFs for collagen, prevent the cells from freely re-organising their cytoskeleton. As a result the mechanotransduction pathways in the cells are desensitised from 2D to collagen gels.

The stiffness of the collagen-based and fibrin-based gels used in this study is an important point for consideration. A detailed study of the mechanical properties of similar TECs was performed by Breidenbach and co-workers^17^. They showed that the material and time in culture affects the mechanical properties of the TECs, with both collagen- and fibrin-based TECs exhibiting increases in linear stiffness and modulus with time in culture^17^. However, both fibrin and collagen TECs approached similar stiffness values when fully contracted between the restraining pins at each end of the TECs. Furthermore, both collagen- and fibrin-based TECs exhibited toe and linear regions in the deformation curves characteristic of viscoelastic tendon tissues^17^.

Although the functional enrichment analysis indicated that similar GO terms (e.g. ‘extracellular region’, ‘extracellular region part’) were upregulated in both culture systems and during tendon development, a closer comparison of genes within these GO terms revealed that fibrin-based TECs were most similar to late tendon development. Pathway analysis to determine the difference between early (E11 to E14) and late (E14 to E17) tendon development and reduced YAP/TAZ activation levels gave an indication that fibrin-based TEC culture might mimic the mechanotransduction pathways in late tendon development for the upregulation of similar genes. Our recent study comparing the mechanical properties of fibrin gel- and collagen gel-derived TECs showed that fibrin-based TECs were mechanically superior^17^. This is explained by the improved alignment of collagen fibrils deposited by fibroblasts cultured in fibrin gels, also observed in this current study.

The ultrastructure of CTFs in fibrin gels was strikingly different to the stellate morphology seen on tissue culture plastic and in 3D collagen gels. CTFs in fibrin-based TECs had a similar shape to CTFs *in vivo* in that they were cylindrical and aligned with the long axis of the TEC. The cells had replaced the fibrin with newly-synthesised collagen fibrils that were parallel (to a close approximation) to the long axis of the TEC. The cells were mechanically integrated into the collagen fibril via their fibripositors^26^,^29^. In contrast, CTFs in collagen-based TECs had long protrusions that reached out in all directions to attach to the pre-existing collagen fibrils in the gel. As a consequence, the cells were stellate in shape, with numerous projections, and thus similar in shape to fibroblasts on 2D tissue culture plastic. Therefore, CTFs in fibrin-based TECs are a better model for tendon development and CTFs in collagen-based TECs are a better model for the stages in dermal wound healing when fibroblasts from the wound edge emerge into the wound bed.

Migrating fibroblasts *in vivo* might be expected to encounter a fibrillar collagen matrix that is disorganised and which they can adhere to. These cells are in a 3D environment but their interaction with an existing collagen matrix could be viewed as 2D. A cell interacting with pre-existing collagen matrix has multiple 2D interactions with collagen fibrils that are themselves mechanically loaded but are collectively mechanically unconnected. A consequence of such an interaction might be the spread of attachment sites in multiple directions, thereby flattening the cell and activating mechanotransduction pathways, such as the YAP/TAZ pathway^31^. Furthermore, in this extended shape new collagen fibrils are likely to be misaligned to the tissue axis, as seen in Fig. S7. Fibrin-based TECs are presumably more similar to the early phase of wound healing in which fibroblasts encounter a fibrin-based granulation tissue that is replaced during the course of wound healing by fibrillar collagen^33^. Within the fibrin-based gel the cells are free to respond to mechanical cues across the wound bed, as shown in the shape of CTFs in Fig. 4c. Furthermore, the collagen fibrils in the fibrin-based cells are in close contact along their length and experience efficient stress transfer between fibrils^17^. We noticed that CTFs in collagen-based gels assemble their own collagen fibrils close to the plasma membrane and in fibripositors. The alignment of the newly-synthesised collagen fibrils was influenced by the shape of the cell, which in turn influenced by the alignment of collagen fibrils in the collagen gel. Interestingly, we observed little evidence of CTFs degrading or remodelling the pre-existing collagen fibrils.

In conclusion, we show that the shape and transcriptome of fibroblasts are strongly influenced by the presence or absence of an existing collagen fibril matrix. When placed in a tensioned degradable matrix, such as tensioned fibrin, fibroblasts can align like close-packed cylinders parallel to the axis of uniaxial tension. The reverse happens in a collagen gel in which the randomly arranged fibrils provide strong points of adhesion for multiple cell processes. As a consequence, the cell adopts a stellate shape that is not too dissimilar to the shape it adopts on 2D tissue culture plastic. The transcriptome of fibroblasts within a fibrin-based TECs most probably resembles that of fibroblasts *in vivo* because the cell shape is not constrained by pre-existing collagen fibrils.

## Materials and Methods

### Animals

All animal work was approved by The University of Manchester Breeding and Supply Unit and was in accordance with the 1986 Home Office Animal Procedures Act (UK), and following local ethical review.

### Embryonic chick tendon and primary culture of tendon fibroblasts

Fertilised chicken eggs were supplied by Henry Stewart and Company (Lincolnshire, UK). Fertilised chicken eggs were incubated at 39 °C in a humidified environment upon delivery. At E11, E14 and E17, eggs were chilled for 30–60 minutes and embryos were decapitated. Metatarsal tendons were pulled and snap frozen for RNA isolation. Tendon fibroblasts were released from E14 tendons by treatment with 1000 U/ml bacterial collagenase type 4 (Worthington Biochemical Corporation) in trypsin (2.5 g/l from Sigma) for 60 to 90 minutes, pipetting every 10 to 15 minutes. Cells were passed through a 70 μm cell strainer, centrifuged for 5 minutes at 400 × *g* and resuspended in Dulbecco’s modified Eagle’s medium containing 4.5 mg/l glucose, non-essential amino acids and 110 mg/l sodium pyruvate (DMEM), supplemented with 10% foetal calf serum (FCS), 2 mM L-glutamine, 10,000 units (U)/ml penicillin and 10 mg/ml streptomycin at 37 °C, 5% CO_2_ in a humidified environment, passaged 1 to 4 when subconfluent and used between passages 2 to 3.

### Preparation of tissue engineered constructs (TECs)

TECs were prepared using the fibrin gel method as previously described^15^. In brief, 6-well culture plates were lined with Sylgard Silicone Elastomer (Dow Corning). Two ~8 mm lengths of Mersilk Suture 3-0 (Ethicon) were placed 1 cm apart and pinned into the silicone with 0.1 mm stainless steel minutien pins (Fine Science Tools GmbH). Plates were sterilised by incubation for 1 hour in 100% ethanol under ultraviolet light and washed with sterile phosphate-buffered saline (PBS) before cells were seeded. For each well, 7.5 × 10^5^ cells in 500 μl fibrin gel solution (final concentration 3.4 mg/ml fibrinogen, from Sigma) were seeded, then incubated for 5 minutes at 37 °C. After, 5 ml of DMEM, supplemented with 10% FCS, 2 mM L-glutamine, 200 μM L-ascorbic acid 2-phosphate, 10,000 U/ml penicillin and 10 mg/ml streptomycin was added to each well. Plates were incubated at 37 °C, 5% CO_2_ in a humidified environment with fresh medium replaced every 2 to 3 days. During medium changes, fibrin gels were scored using a sterile pipette tip to detach the edges of fibrin gels and allow them to contract and form a 3D tendon-like tissue construct between the two sutures. Point of full contraction took 10 days. Maturation of 3D fibrin-based TECs was achieved by culturing from point of contraction under tension for an additional 10 days.

TECs were prepared using the collagen gel method as described previously^34^, in combination with the plate set up described above for fibrin-based gels. In brief, 7.5 × 10^5^ cells was suspended in 237 μl rat tail collagen type I (Life Technologies; final concentration 2.6 mg/ml) in 25× DPBS (Sigma), pH 7 and then incubated for 45 minutes at 37 °C. After, the collagen gels were cultured in the same medium and in the same manner as the fibrin-based gels. As the collagen gels contract faster than the fibrin-based gels, point of full contraction took ~3 days and gels could only be matured for an additional 3 days.

### RNA isolation and microarray

For the isolation of RNA from dissected tendons and 3D TECs, samples were washed with ice-cold PBS 3 times and snap frozen in liquid nitrogen with 500 μl TRIzol. Frozen tissues were homogenised using a Mikro-Dismembrator U (B. Braun Biotech International) at 2000 Hz for 90 seconds two times. The volumes of dismembrated samples were made up to 1 ml with TRIzol and CTFs in 2D were lysed with 1 ml TRIzol before continuing with the manufacturer’s instructions. Integrity and measurement of total RNA was further performed using Agilent 2100 Bioanalyzer (Agilent Technologies). Labelled cRNA was synthesised and hybridised to Mouse Genome 430 2.0 GeneChip arrays (Affymetrix). Microarray data are available in the ArrayExpress database (www.ebi.ac.uk/arrayexpress) under accession number E-MTAB-3488.

### Microarray analysis

Microarray data sets were analysed by dChip (DNA-Chip) Analyzer and boxplots^35^ to detect outlierarrays (date of access 27^th^ July 2015). Microarrays were analysed using the Robust Multichip Average (RMA) method^36^. Differential expression analysis was performed using Limma using the functions lmFit and eBayes^37^. Gene lists of differentially expressed genes were controlled for false discovery rate (fdr) errors using the method of QVALUE^38^.

### Functional annotation and pathway analysis

Gene ontology analysis was performed on probe sets that detected fold changes ≥±2 (*q* < 0.001) between stages of tendon development or steps of *in vitro* TEC formation using the Database for Annotation, Visualisation and Integrated Discovery (DAVID) online tool^39^. Probe sets differentially expressed (fold changes ≥±2, *q* < 0.05) between E11 and E14, E14 and E17, and E11 and E17 of tendon development were subjected to PCA and a *k*-means clustering algorithm. *k*-means clustering was performed on the basis of similarity of profiles (Manhattan Distance) across the dataset using the ‘Super Grouper’ plugin of maxdView software (available from http://bioinf.man.ac.uk/microarray/maxd/). Clustering was performed on the means of each sample group (log2) that had been z-transformed (for each probe set the mean was set to 0, standard deviation to 1). A similar normalisation was applied to the fibrin- and collagen-based TEC datasets, although they were not used in the clustering process. Data from each expression profile cluster was evaluated using DAVID. Venn diagrams were generated using Venny 2.0.2 online tool^40^. Probe sets expressed differentially (fold changes ≥±2, *q* < 0.05) between E11 and E14, and E14 and E17 were evaluated using Ingenuity Pathway Analysis (IPA; Qiagen). IPA maps each gene within a global molecular network developed from information contained in the Ingenuity Pathways Knowledge Base. Gene networks were generated algorithmically based on their connectivity in terms of expression, activation and transcription.

### Electron microscopy

Tissues were prepared for transmission electron microscopy and serial block face-scanning electron microscopy (SBF-SEM) and 3D reconstructions were generated as previously described^25^.

## Additional Information

**How to cite this article**: Yeung, C.-Y. C. *et al.* Chick tendon fibroblast transcriptome and shape depend on whether the cell has made its own collagen matrix. *Sci. Rep.* **5**, 13555; doi: 10.1038/srep13555 (2015).

## Supplementary Material

Supplementary Movie 1

Supplementary Movie 2

Supplementary Information
