# Supplementary material for: Chick tendon fibroblast transcriptome and shape depend on whether the cell has made its own collagen matrix
Source: Sci Rep. 2015 Sep 4;5:13555. doi: 10.1038/srep13555 (PMC4559659; doi:10.1038/srep13555)
Supplement: Supplementary Information [file srep13555-s3.pdf]

Supplementary information for:

Chick tendon fibroblast transcriptome and shape depend on whether the cell has made its own collagen matrix

Ching-Yan Chloé Yeung[1,2]*, Leo A. H. Zeef[2], Chloe Lallyett[1,2], Yinhui Lu[1], Elizabeth G. Canty-Laird[3,4] and Karl E. Kadler[1,2]*

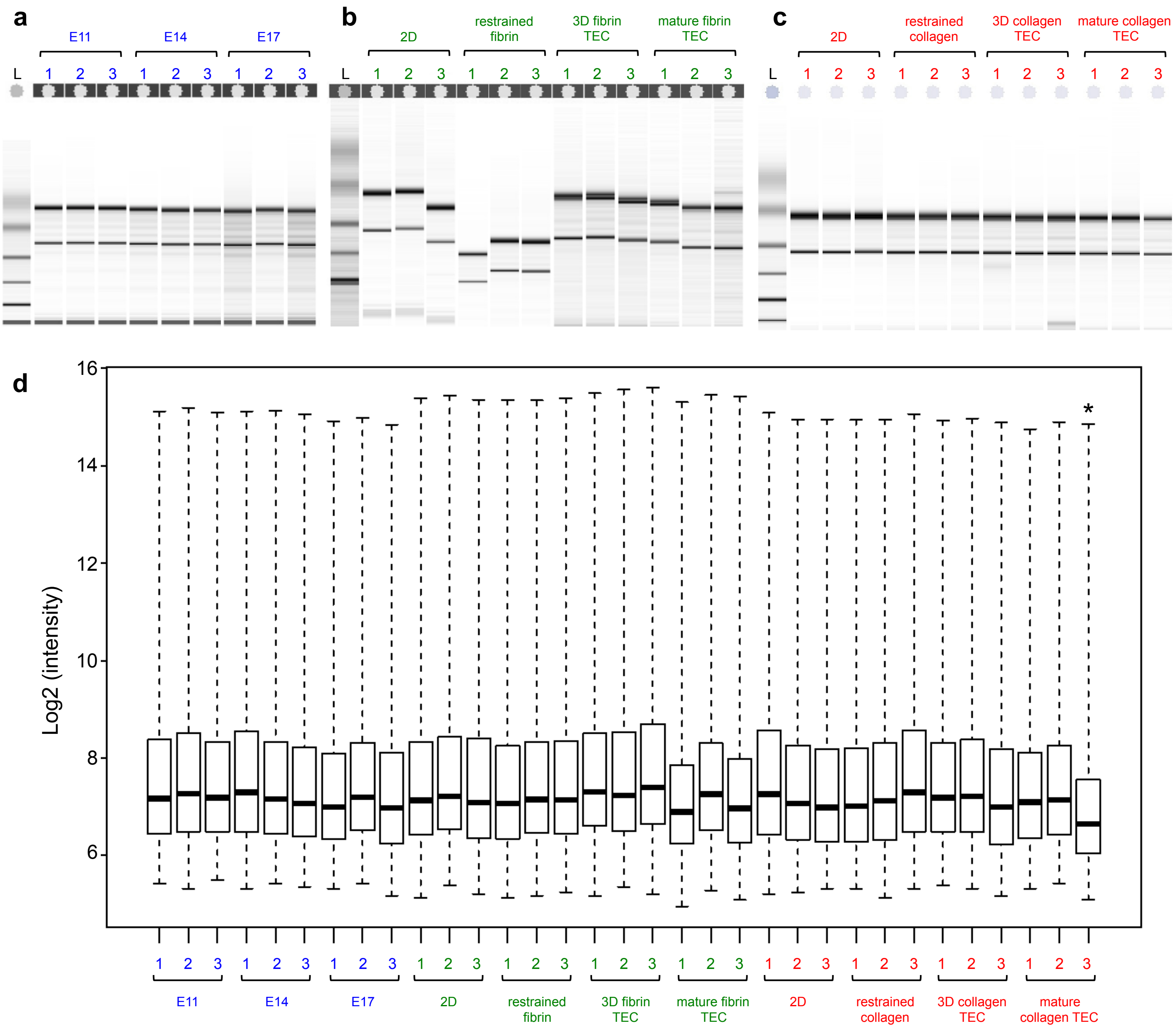
a
E11
E14
E17
L 1 2 3 1 2 3 1 2 3
b
2D
restrained fibrin
3D fibrin TEC
mature fibrin TEC
L 1 2 3 1 2 3 1 2 3 1 2 3
c
2D
restrained collagen
3D collagen TEC
mature collagen TEC
L 1 2 3 1 2 3 1 2 3 1 2 3
d
Log2 (intensity)
16
14
12
10
8
6
*
1 2 3 1 2 3 1 2 3 1 2 3 1 2 3 1 2 3 1 2 3 1 2 3 1 2 3 1 2 3 1 2 3
E11
E14
E17
2D
restrained fibrin
3D fibrin TEC
mature fibrin TEC
2D
restrained collagen
3D collagen TEC
mature collagen TEC

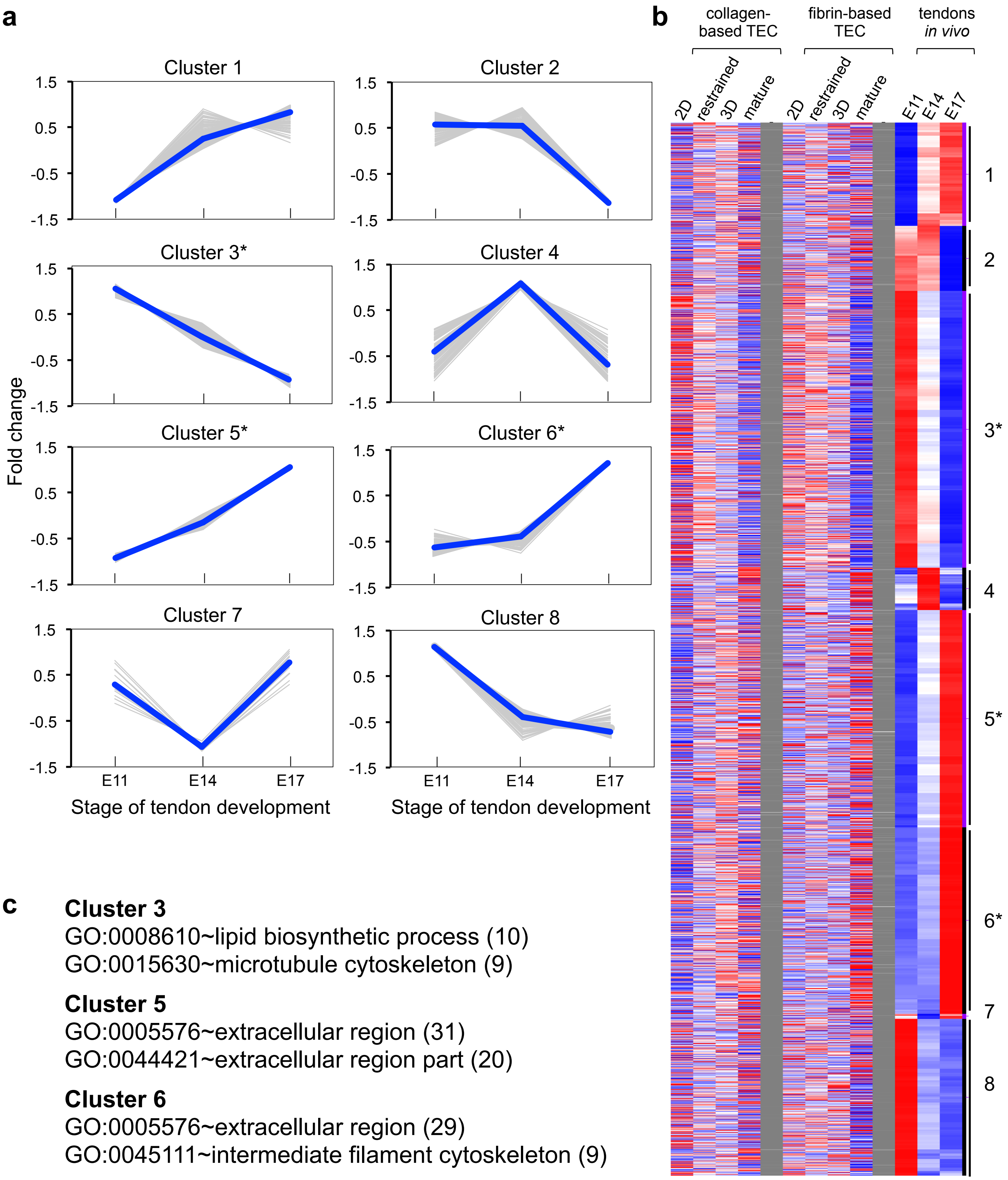

c

Cluster 3
GO:0008610~lipid biosynthetic process (10)
GO:0015630~microtubule cytoskeleton (9)

Cluster 5
GO:0005576~extracellular region (31)
GO:0044421~extracellular region part (20)

Cluster 6
GO:0005576~extracellular region (29)
GO:0045111~intermediate filament cytoskeleton (9)

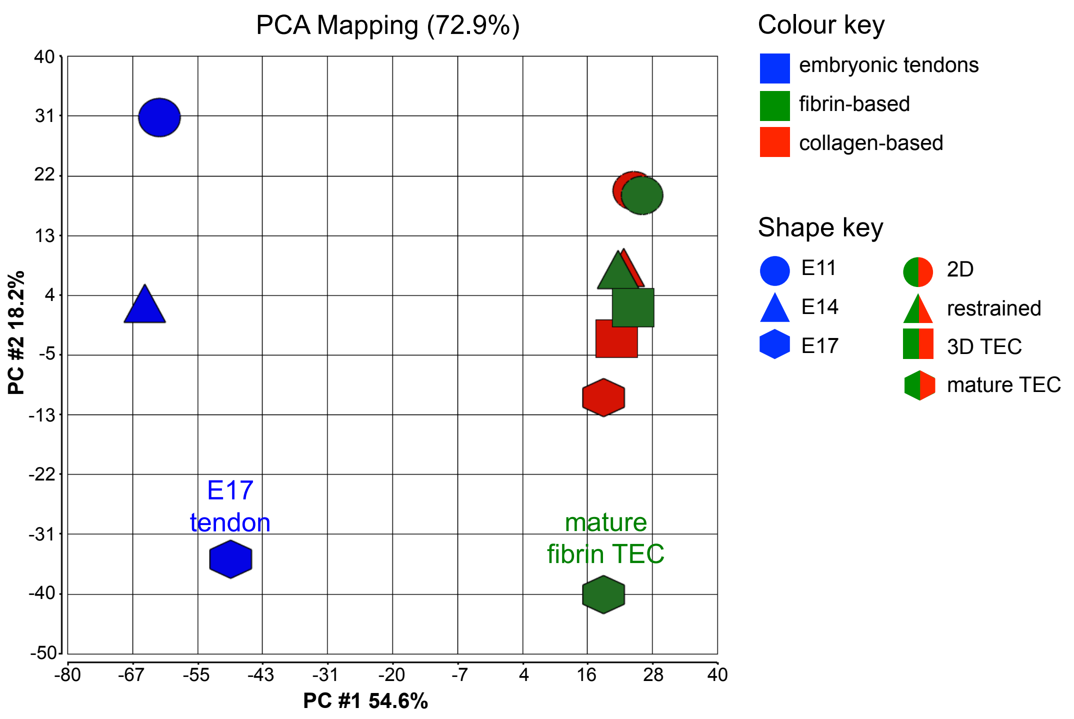

PCA Mapping (72.9%)
PC #2 18.2%
PC #1 54.6%
40
31
22
13
4
-5
-13
-22
-31
-40
-50
-80
-67
-55
-43
-31
-20
-7
4
16
28
40
E17
tendon
mature
fibrin TEC
Colour key
embryonic tendons
fibrin-based
collagen-based
Shape key
E11
E14
E17
2D
restrained
3D TEC
mature TEC

a

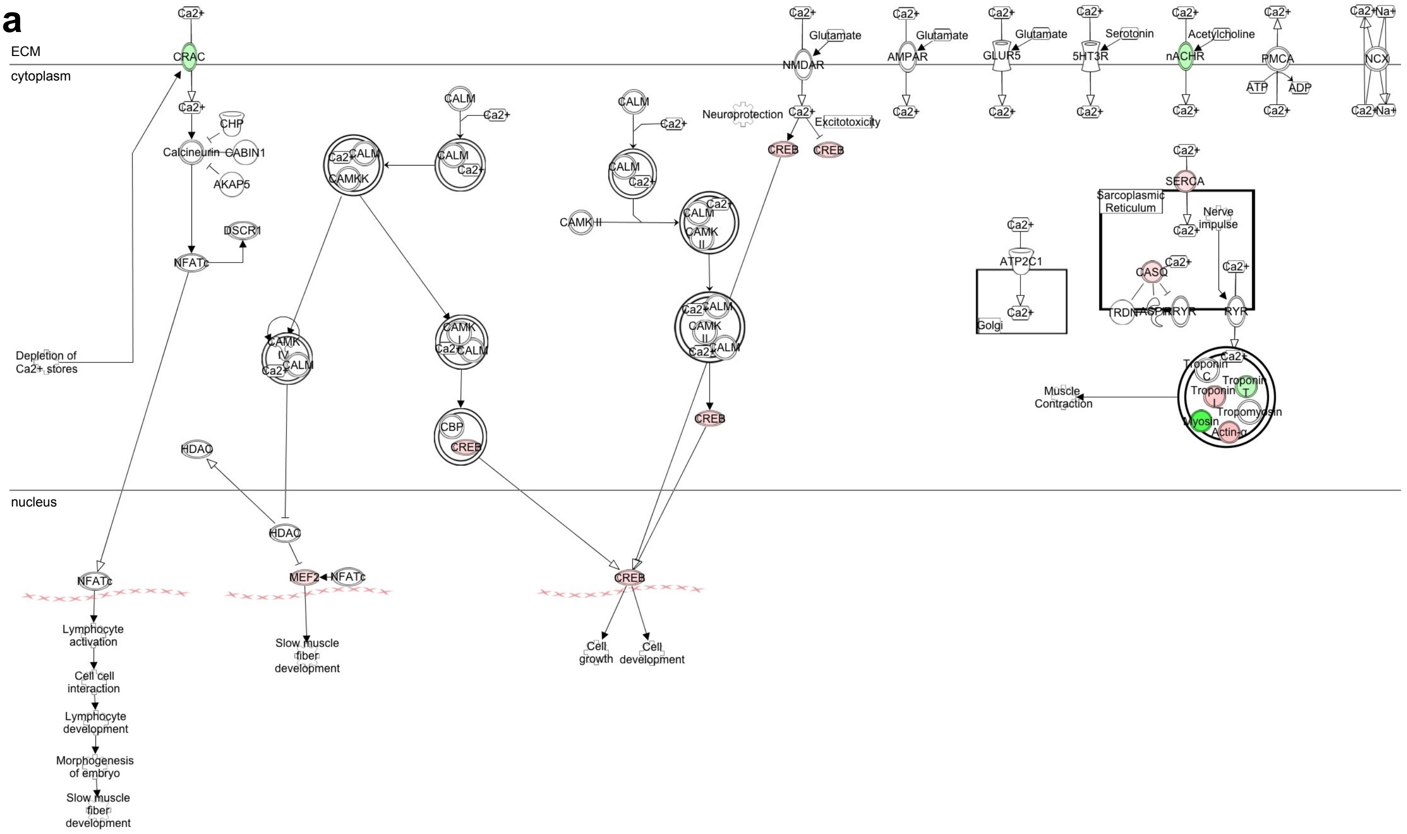
ECM
cytoplasm
nucleus
Ca2+
CRAC
Calcineurin
CHP
CABIN1
AKAP5
DSCR1
NFATc
Depletion of Ca2+ stores
CALM
CAMKK
CAMK IV
CAMK I
CAMK II
CBP
CREB
HDAC
MEF2
NMDAR
Glutamate
AMPAR
GLUR5
5HT3R
Serotonin
nAChR
Acetylcholine
PMCA
ATP
ADP
NCX
Na+
Neuroprotection
Excitotoxicity
SERCA
Sarcoplasmic Reticulum
Nerve impulse
CASQ
TRDN
RYR
ATP2C1
Golgi
Troponin C
Troponin T
Troponin I
Tropomyosin
Myosin
Actin-α
Muscle Contraction
Lymphocyte activation
Cell cell interaction
Lymphocyte development
Morphogenesis of embryo
Slow muscle fiber development
Cell growth
Cell development

b

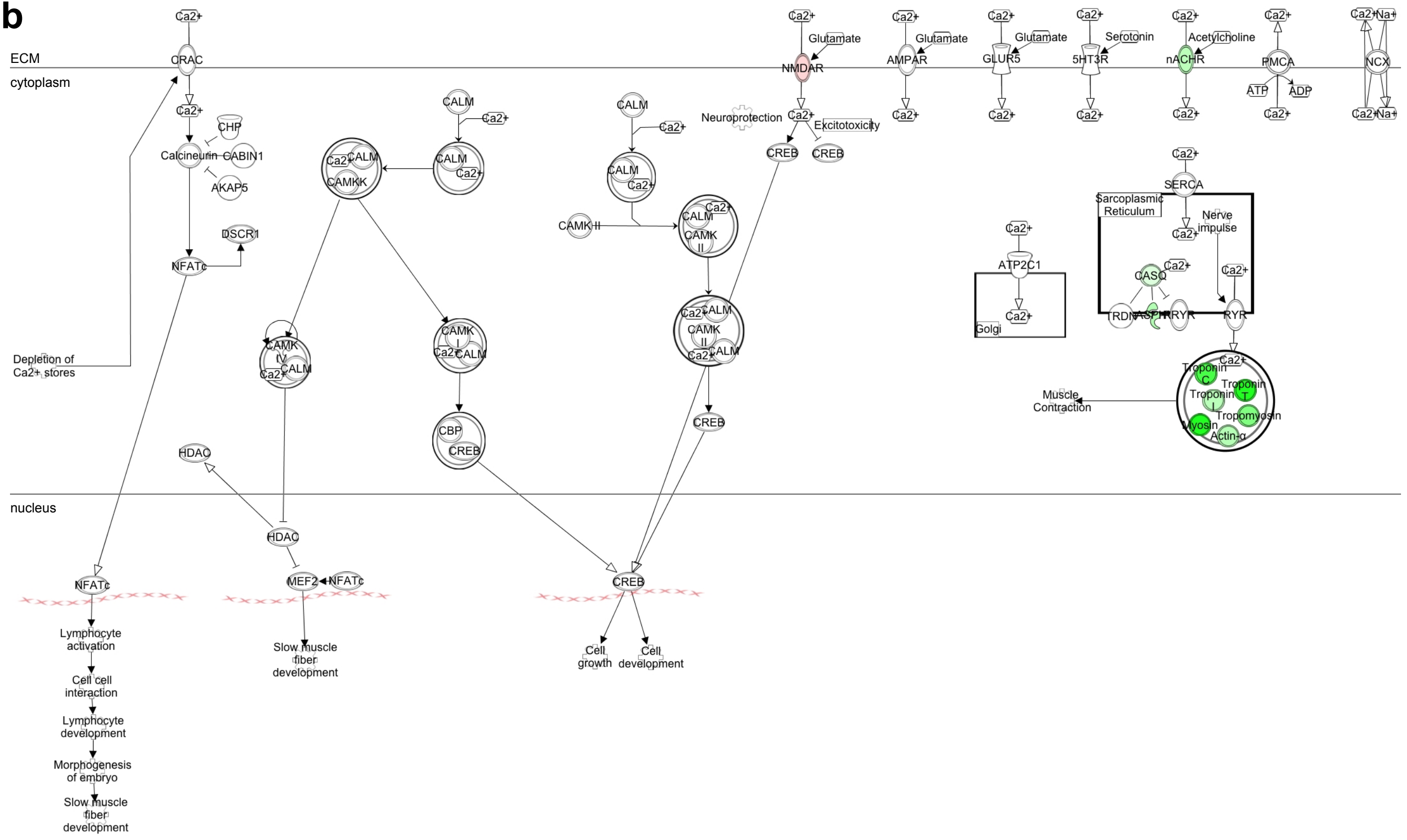
ECM
cytoplasm
nucleus
Ca2+
CRAC
Calcineurin
CHP
CABIN1
AKAP5
DSCR1
NFATc
Depletion of Ca2+ stores
CALM
CAMKK
CAMK IV
CAMK I
CAMK II
CBP
CREB
HDAC
MEF2
NMDAR
Glutamate
AMPAR
GLUR5
5HT3R
Serotonin
nAChR
Acetylcholine
PMCA
ATP
ADP
NCX
Na+
Neuroprotection
Excitotoxicity
SERCA
Sarcoplasmic Reticulum
Nerve impulse
CASQ
TRDN
RYR
ATP2C1
Golgi
Troponin C
Troponin T
Troponin I
Tropomyosin
Myosin
Actin-α
Muscle Contraction
Lymphocyte activation
Cell cell interaction
Lymphocyte development
Morphogenesis of embryo
Slow muscle fiber development
Cell growth
Cell development

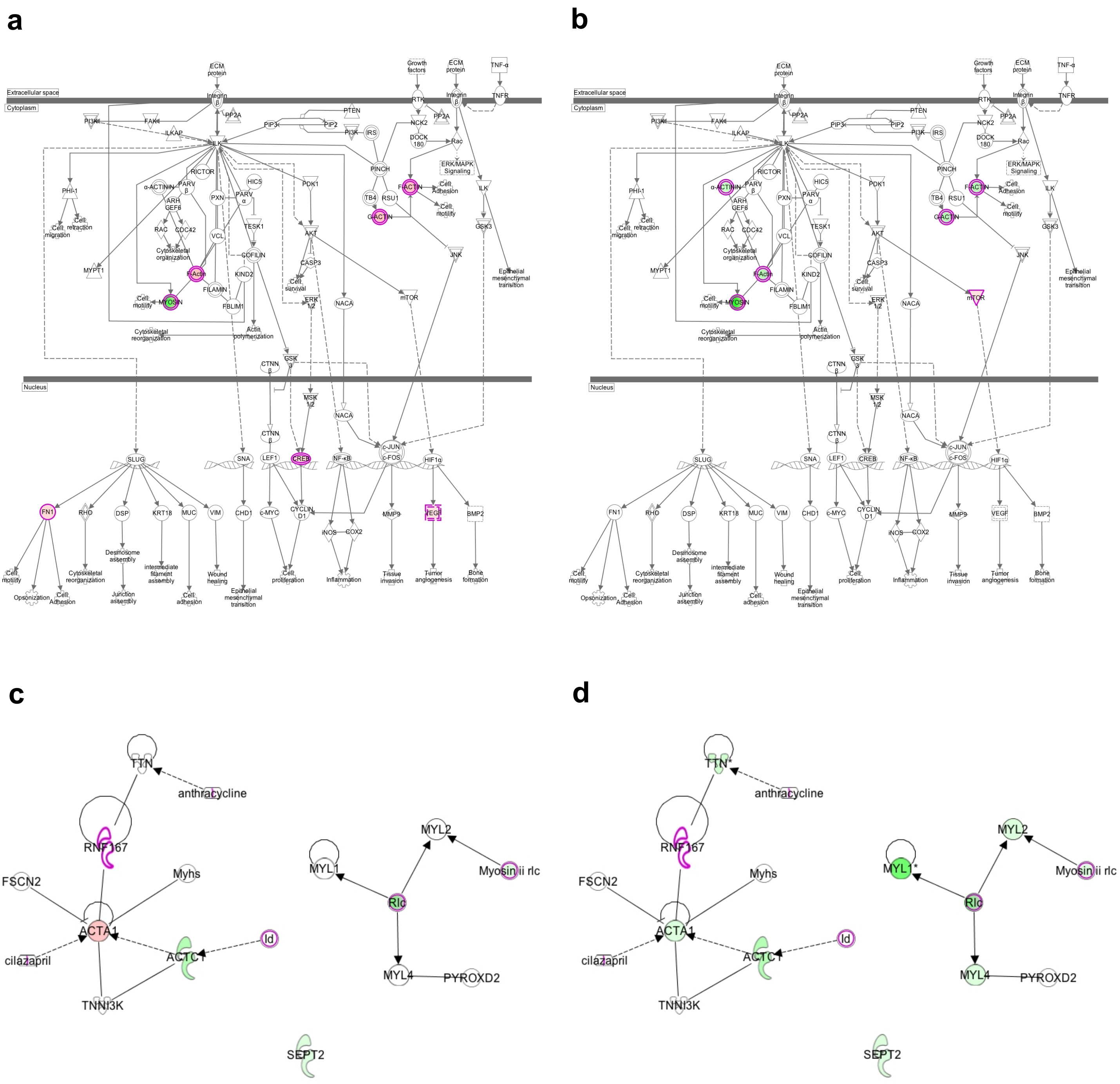
a
b
c
d
Extracellular space
Cytoplasm
Nucleus
ECM protein
Growth factors
TNF-α
Integrin β
RTK
TNFR
PI3Kt
FAK
PP2A
ILKAP
ILK
PIP3
PIP2
PTEN
PI3K
IRS
NCK2
DOCK 180
Rac
ERK/MAPK Signaling
PINCH
PHI-1
α-ACTININ
PARV β
RICTOR
HIC5
PDK1
F-ACTIN
TB4
RSU1
G-ACTIN
Cell Adhesion
Cell motility
Cell migration
Cell retraction
ARH GEF6
RAC
CDC42
Cytoskeletal organization
PXN
PARV α
VCL
TESK1
COFILIN
AKT
CASP3
Cell survival
ERK 1/2
NACA
mTOR
JNK
GSK3
Epithelial mesenchymal transition
MYPT1
F-Actin
KIND2
FILAMIN
MYOSIN
FBLIM1
Cytoskeletal reorganization
Actin polymerization
CTNN β
GSK 3
MSK 1/2
c-JUN
c-FOS
SLUG
SNA
LEF1
CREB
NF-κB
HIF1α
FN1
RHO
DSP
KRT18
MUC
VIM
CHD1
c-MYC
CYCLIN D1
iNOS
COX2
MMP9
VEGF
BMP2
Opsonization
Desmosome assembly
Junction assembly
intermediate filament assembly
Cell adhesion
Wound healing
Cell proliferation
Inflammation
Tissue invasion
Tumor angiogenesis
Bone formation
TTN
anthracycline
RNF167
FSCN2
Myhs
ACTA1
cilazapril
ACTC1
Id
TNNI3K
MYL2
MYL1
Myosin ii rlc
Rlc
MYL4
PYROXD2
SEPT2
TTN*
MYL1*

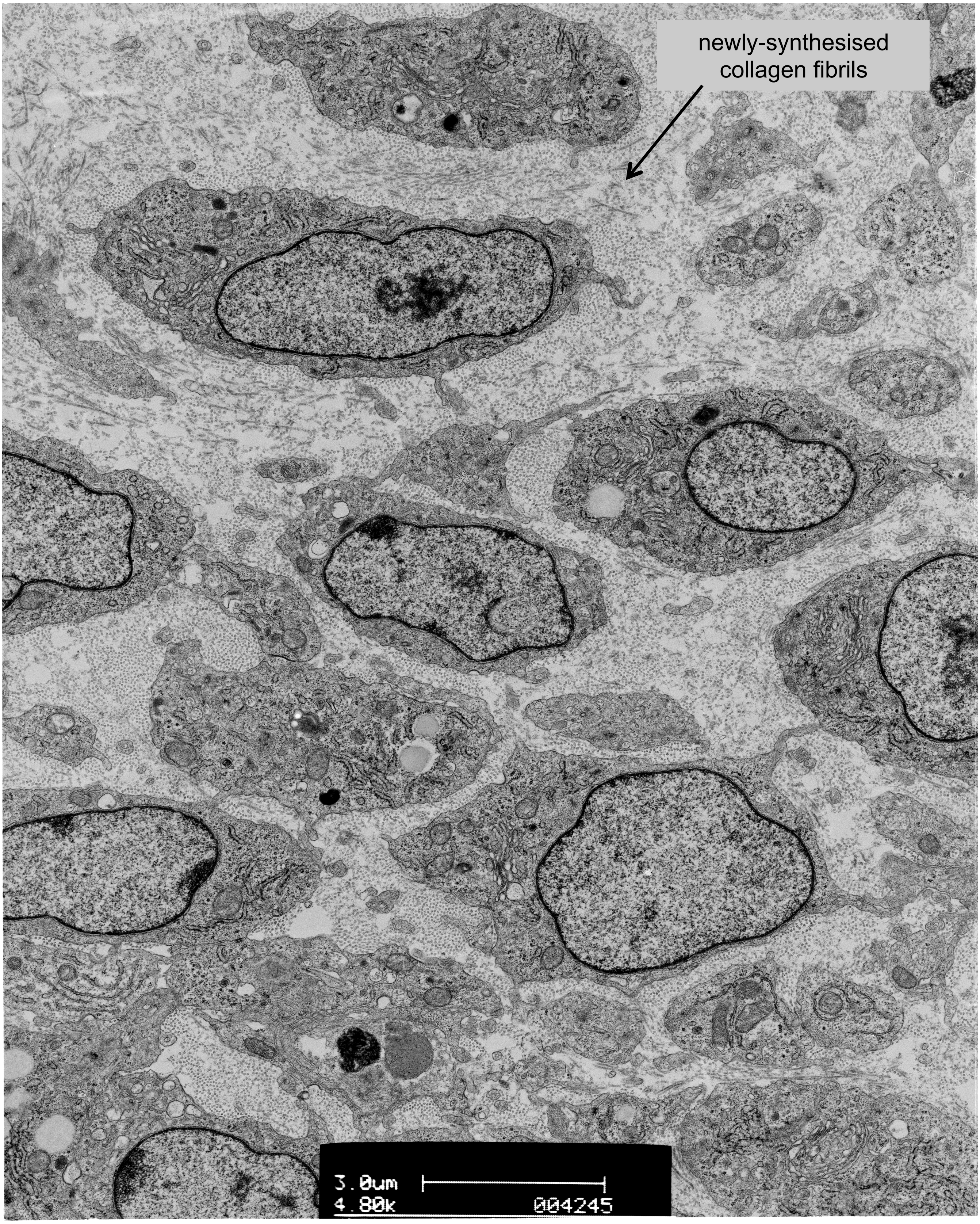
newly-synthesised collagen fibrils
3.0um
4.80k
004245

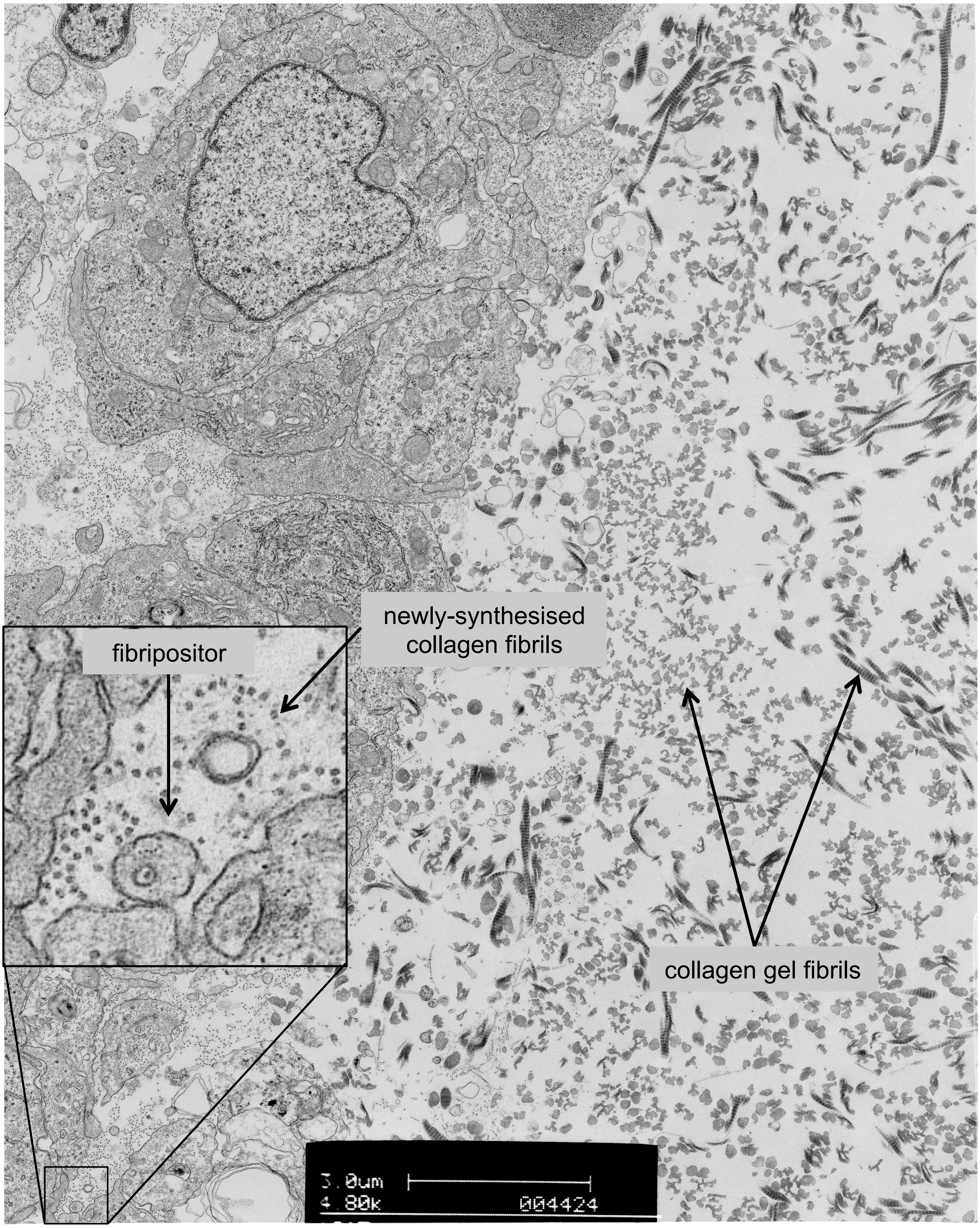
newly-synthesised
collagen fibrils
fibripositor
collagen gel fibrils
3.0um
4.80k
004424

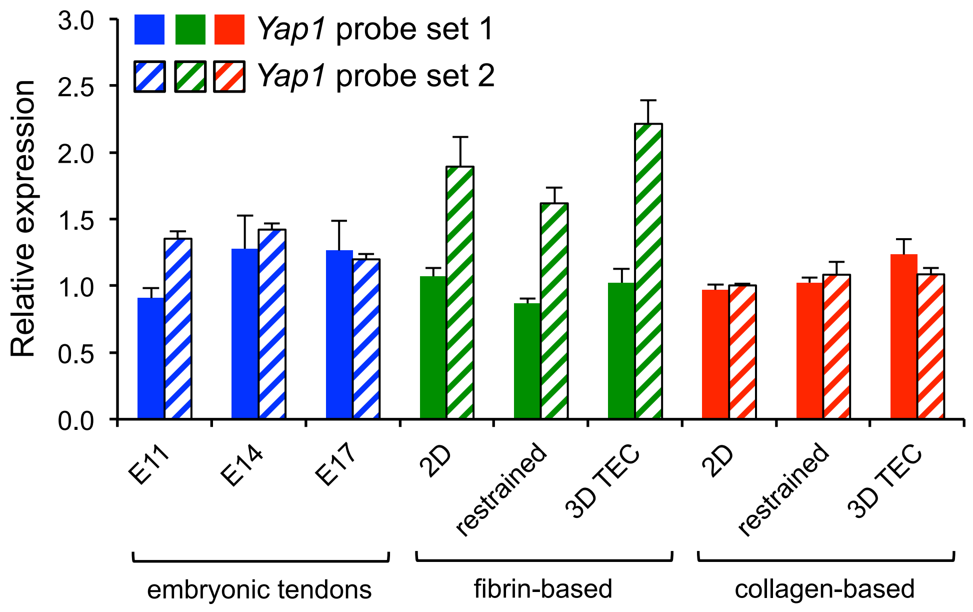

Yap1 probe set 1
Yap1 probe set 2
Relative expression
3.0
2.5
2.0
1.5
1.0
0.5
0.0
E11
E14
E17
2D
restrained
3D TEC
2D
restrained
3D TEC
embryonic tendons
fibrin-based
collagen-based

Supplementary Table 1. List of genes expressed during chick embryonic tendon development.

Total probe sets with significant ≥2-fold difference bewteen E11 tendon and E14 tendon = 459 (q < 0.01)

Top annotation clusters upregulated from E11 tendon and E14 tendon (from 289 probe sets)

**Enrichment Score: 4.840541620180573**	**GOTERM_CC_FAT: 0005576~extracellular region**	**Count: 29**	***p* = 1.97E-08**
GGA.3033.1.S1_AT	similar to Small inducible cytokine A13 precursor (CCL13) (Monocyte chemotactic protein 4) (MCP-4) (Monocyte chemoattractant protein 4) (CK-beta-10) (NCC-1)		
GGAAFFX.10135.1.S1_AT	CD109 molecule		
GGAAFFX.24555.1.S1_S_AT	collagen, type X, alpha 1(Schmid metaphyseal chondrodysplasia)		
GGA.150.2.S2_AT	fms-related tyrosine kinase 1 (vascular endothelial growth factor/vascular permeability factor receptor)		
GGA.4248.1.S1_AT	lipoprotein lipase		
GGAAFFX.21769.1.S1_S_AT	lipoprotein lipase		
GGAAFFX.25194.1.S1_AT	angiopoietin-like 5		
GGAAFFX.102.1.S1_AT	ADAM metallopeptidase with thrombospondin type 1 motif, 19		
GGA.3641.1.S1_AT	annexin A2		
GGA.1328.1.S2_AT	beta-2-microglobulin		
GGA.16.1.S1_AT	ghrelin/obestatin preprohormone		
GGA.4257.1.S1_AT	collagen, type VI, alpha 2		
GGA.19409.1.S1_S_AT	collagen, type IV, alpha 2		
GGA.3572.1.S2_S_AT	CD44 molecule (Indian blood group)		
GGA.606.1.S1_AT	v-kit Hardy-Zuckerman 4 feline sarcoma viral oncogene homolog		
GGA.10439.1.S1_AT	fibronectin 1		
GGA.822.1.S1_A_AT	elastin (supravalvular aortic stenosis, Williams-Beuren syndrome)		
GGA.540.1.S1_AT	matrix Gla protein		
GGA.690.1.S1_AT	lymphocyte antigen 86		
GGA.888.1.S1_AT	collagen, type VI, alpha 3		
GGA.3745.1.S1_AT	carboxypeptidase Z; similar to carboxypeptidase Z		
GGA.3745.1.S2_AT	carboxypeptidase Z; similar to carboxypeptidase Z		
GGA.16038.1.S1_AT	adiponectin, C1Q and collagen domain containing		
GGA.4719.1.S1_S_AT	apolipoprotein A-I		
GGA.4328.1.S1_AT	insulin-like growth factor binding protein 7		
GGA.3587.1.S1_S_AT	matrilin 3		
GGA.19049.1.S1_AT	interleukin 18 (interferon-gamma-inducing factor)		
GGA.17040.2.S1_A_AT	fibroblast growth factor 2 (basic)		
GGA.6218.1.S1_AT	microfibrillar associated protein 5		
GGAAFFX.24388.1.S1_AT	microfibrillar associated protein 5		
GGA.653.1.S1_AT	tenascin XB		
GGA.9725.1.S1_AT	angiopoietin-like 2		

**Enrichment Score: 3.573630556708628**	**GOTERM_BP_FAT: 0022610~biological adhesion**	**Count: 13**	***p* = 1.22E-04**
GGA.5171.1.S1_AT	cadherin 5, type 2, VE-cadherin (vascular epithelium)		
GGA.888.1.S1_AT	collagen, type VI, alpha 3		
GGA.3665.1.S2_AT	cadherin 20, type 2		
GGA.4394.1.S1_AT	B-cell CLL/lymphoma 2		
GGA.4394.1.S2_AT	B-cell CLL/lymphoma 2		
GGA.14634.1.S1_AT	B-cell CLL/lymphoma 2		
GGA.4394.1.S2_S_AT	B-cell CLL/lymphoma 2		
GGAAFFX.25095.1.S1_AT	integrin, beta-like 1 (with EGF-like repeat domains)		
GGAAFFX.10422.3.S1_S_AT	collagen, type XXI, alpha 1		
GGAAFFX.10422.1.S1_AT	collagen, type XXI, alpha 1		
GGA.4257.1.S1_AT	collagen, type VI, alpha 2		
GGA.566.1.S1_AT	integrin, alpha 1		
GGA.3572.1.S2_S_AT	CD44 molecule (Indian blood group)		
GGA.4017.1.S1_AT	ezrin		
GGA.10439.1.S1_AT	fibronectin 1		
GGA.13583.1.S1_AT	similar to fatty acid translocase; similar to CD36 molecule (thrombospondin receptor); CD36 molecule (thrombospondin receptor)		
GGAAFFX.11849.1.S1_S_AT	similar to fatty acid translocase; similar to CD36 molecule (thrombospondin receptor); CD36 molecule (thrombospondin receptor)		
GGA.20061.1.S1_AT	ninjurin 2		

**Enrichment Score: 3.573630556708628**	**GOTERM_MF_FAT: 0005539~glycosaminoglycan binding**	**Count: 5**	***p* = 7.47E-04**
GGA.3572.1.S2_S_AT	CD44 molecule (Indian blood group)		

GGAAFFX.21769.1.S1_S_AT	lipoprotein lipase
GGA.4248.1.S1_AT	lipoprotein lipase
GGA.10439.1.S1_AT	fibronectin 1
GGA.17040.2.S1_A_AT	fibroblast growth factor 2 (basic)
GGAAFFX.9770.3.S1_S_AT	interphotoreceptor matrix proteoglycan 2

**Enrichment Score: 2.5463222084174273**	**UP_SEQ_FEATURE: signal peptide**	**Count: 19**	***p* = 8.06E-04**
GGA.3332.1.S1_AT	matrix-remodelling associated 8		
GGA.540.1.S1_AT	matrix Gla protein		
GGA.5171.1.S1_AT	cadherin 5, type 2, VE-cadherin (vascular epithelium)		
GGA.690.1.S1_AT	lymphocyte antigen 86		
GGA.888.1.S1_AT	collagen, type VI, alpha 3		
GGAAFFX.24555.1.S1_S_AT	collagen, type X, alpha 1(Schmid metaphyseal chondrodysplasia)		
GGA.3665.1.S2_AT	cadherin 20, type 2		
GGA.4248.1.S1_AT	lipoprotein lipase		
GGAAFFX.21769.1.S1_S_AT	lipoprotein lipase		
GGA.3745.1.S2_AT	carboxypeptidase Z; similar to carboxypeptidase Z		
GGA.3745.1.S1_AT	carboxypeptidase Z; similar to carboxypeptidase Z		
GGA.150.2.S2_AT	fms-related tyrosine kinase 1 (vascular endothelial growth factor/vascular permeability factor receptor)		
GGAAFFX.9770.3.S1_S_AT	interphotoreceptor matrix proteoglycan 2		
GGA.1328.1.S2_AT	beta-2-microglobulin		
GGA.16.1.S1_AT	ghrelin/obestatin preprohormone		
GGA.4257.1.S1_AT	collagen, type VI, alpha 2		
GGA.4719.1.S1_S_AT	apolipoprotein A-I		
GGA.3587.1.S1_S_AT	matrilin 3		
GGA.606.1.S1_AT	v-kit Hardy-Zuckerman 4 feline sarcoma viral oncogene homolog		
GGA.822.1.S1_A_AT	elastin (supravalvular aortic stenosis, Williams-Beuren syndrome)		
GGA.653.1.S1_AT	tenascin XB		

Top annotation clusters downregulated from E11 tendon and E14 tendon (from 170 probe sets)

**Enrichment Score: 1.7096809121359748**	**GOTERM_BP_FAT: 0006941~striated muscle contraction**	**Count: 3**	***p* = 0.0077641**
GGA.5135.1.S1_S_AT	myosin, heavy chain 7, cardiac muscle, beta; similar to slow myosin heavy chain 3		
GGA.4134.1.S2_AT	cholinergic receptor, nicotinic, alpha 1 (muscle)		
GGA.2617.1.S1_AT	myosin, heavy polypeptide 6, cardiac muscle, alpha		

**Enrichment Score: 1.3697230673287633**	**GOTERM_MF_FAT: GO:0020037~heme binding**	**Count: 5**	***p* = 0.0044964**
GGA.11099.1.S1_AT	guanylate cyclase 1, soluble, beta 2		
GGAAFFX.7307.1.S1_AT	cytochrome P450, family 27, subfamily C, polypeptide 1		
GGAAFFX.6670.1.S1_AT	cytochrome P450, family 1, subfamily B, polypeptide 1		
GGA.1960.2.S1_A_AT	myoglobin		
GGA.331.1.S1_AT	cytochrome P450, family 26, subfamily A, polypeptide 1		

Total probe sets with significant ≥2-fold difference bewteen E14 tendon and E17 tendon = 656 (q < 0.01)

Top annotation clusters upregulated from E14 tendon and E17 tendon (from 329 probe sets)

**Enrichment Score: 5.360570535321089**	**GOTERM_CC_FAT: 0005576~extracellular region**	**Count: 31**	***p* = 6.21E-09**
GGA.837.1.S1_A_AT	neuropeptide Y		
GGA.19956.1.S1_S_AT	chitinase, acidic		
GGAAFFX.25194.1.S1_AT	angiopoietin-like 5		
GGA.1328.1.S2_AT	beta-2-microglobulin		
GGAAFFX.21581.1.S1_S_AT	chemokine (C-X-C motif) ligand 14		
GGA.19409.1.S1_S_AT	collagen, type IV, alpha 2		
GGA.3104.1.S1_AT	collagen, type IV, alpha 2		
GGA.3357.2.S1_A_AT	lysozyme G-like 2		
GGA.1148.1.S2_AT	ST6 beta-galactosamide alpha-2,6-sialyltranferase 1		
GGA.1148.1.S1_AT	ST6 beta-galactosamide alpha-2,6-sialyltranferase 1		
GGA.2551.2.S1_A_AT	lactotransferrin		
GGA.2551.2.S1_S_AT	lactotransferrin		
GGAAFFX.21842.1.S1_S_AT	Gal 7		
GGA.822.1.S1_A_AT	elastin (supravalvular aortic stenosis, Williams-Beuren syndrome)		
GGA.540.1.S1_AT	matrix Gla protein		
GGA.690.1.S1_AT	lymphocyte antigen 86		

GGA.701.1.S1_AT	leukocyte ribonuclease A-1; leukocyte ribonuclease A-2
GGA.3972.1.S1_AT	sema domain, immunoglobulin domain (Ig), short basic domain, secreted, (semaphorin) 3D
GGA.100.1.S1_A_AT	Fas (TNF receptor superfamily, member 6)
GGA.729.1.S1_AT	mature avidin; similar to Avidin-related protein 3
GGA.155.1.S1_S_AT	lectin, galactoside-binding, soluble, 3
GGA.3745.1.S1_AT	carboxypeptidase Z; similar to carboxypeptidase Z
GGA.3745.1.S2_AT	carboxypeptidase Z; similar to carboxypeptidase Z
GGA.3928.1.S1_AT	hyaluronan and proteoglycan link protein 1
GGA.13484.1.S1_AT	interleukin 15
GGA.5847.1.S1_AT	ProSAPiP1 protein
GGA.16038.1.S1_AT	adiponectin, C1Q and collagen domain containing
GGAAFFX.2012.1.S1_AT	angiopoietin-like 7
GGA.176.1.S1_A_AT	gallinacin 1
GGAAFFX.9557.1.S1_S_AT	laminin, alpha 3
GGA.4719.1.S1_S_AT	apolipoprotein A-I
GGA.3587.1.S1_AT	matrilin 3
GGA.3587.1.S1_S_AT	matrilin 3
GGA.9962.1.S1_AT	ectonucleoside triphosphate diphosphohydrolase 1
GGAAFFX.24388.1.S1_AT	microfibrillar associated protein 5
GGA.653.1.S1_AT	tenascin XB

**Enrichment Score: 2.293252046433252**	**GOTERM_CC_FAT: GO:0044421~extracellular region part**	**Count: 16**	***p* = 2.95E-04**
GGA.690.1.S1_AT	lymphocyte antigen 86		
GGA.155.1.S1_S_AT	lectin, galactoside-binding, soluble, 3		
GGA.3928.1.S1_AT	hyaluronan and proteoglycan link protein 1		
GGA.3745.1.S2_AT	carboxypeptidase Z; similar to carboxypeptidase Z		
GGA.3745.1.S1_AT	carboxypeptidase Z; similar to carboxypeptidase Z		
GGA.13484.1.S1_AT	interleukin 15		
GGAAFFX.25194.1.S1_AT	angiopoietin-like 5		
GGA.16038.1.S1_AT	adiponectin, C1Q and collagen domain containing		
GGAAFFX.2012.1.S1_AT	angiopoietin-like 7		
GGAAFFX.9557.1.S1_S_AT	laminin, alpha 3		
GGA.4719.1.S1_S_AT	apolipoprotein A-I		
GGA.3104.1.S1_AT	collagen, type IV, alpha 2		
GGA.19409.1.S1_S_AT	collagen, type IV, alpha 2		
GGA.3587.1.S1_AT	matrilin 3		
GGA.3587.1.S1_S_AT	matrilin 3		
GGA.9962.1.S1_AT	ectonucleoside triphosphate diphosphohydrolase 1		
GGAAFFX.24388.1.S1_AT	microfibrillar associated protein 5		
GGA.822.1.S1_A_AT	elastin (supravalvular aortic stenosis, Williams-Beuren syndrome)		
GGA.653.1.S1_AT	tenascin XB		

**Enrichment Score: 2.293252046433252**	**GOTERM_BP_FAT: 0030155~regulation of cell adhesion**	**Count: 5**	***p* = 0.0025264**
GGAAFFX.9557.1.S1_S_AT	laminin, alpha 3		
GGAAFFX.11648.1.S1_AT	B-cell CLL/lymphoma 6 (zinc finger protein 51)		
GGA.16038.1.S1_AT	adiponectin, C1Q and collagen domain containing		
GGA.13583.1.S1_AT	molecule (thrombospondin receptor)		
GGAAFFX.11849.1.S1_S_AT	molecule (thrombospondin receptor)		
GGA.4394.1.S2_AT	B-cell CLL/lymphoma 2		
GGA.14634.1.S1_AT	B-cell CLL/lymphoma 2		
GGA.4394.1.S2_S_AT	B-cell CLL/lymphoma 2		
GGA.4394.1.S1_AT	B-cell CLL/lymphoma 2		

Top annotation clusters downregulated from E14 tendon and E17 tendon (from 327 probe sets)

**Enrichment Score: 4.027321125994694**	**GOTERM_MF_FAT: :0003779~actin binding**	**Count: 11**	***p* = 3.84E-06**
GGA.4958.1.S1_AT	xin actin-binding repeat containing 2		
GGA.700.1.S1_AT	troponin I type 2 (skeletal, fast)		
GGA.4217.2.S1_AT	myosin, heavy chain 6, cardiac muscle, alpha; similar to fast myosin heavy chain HCIII; myosin, heavy chain 8, skeletal muscle, perinatal; myosin, heavy chain 1, skeletal muscle, adult; myosin, heavy chain 3, skeletal muscle, embryonic; similar to fast myosin heavy chain HCII; myosin, heavy chain 2, skeletal muscle, adult		
GGA.4217.5.S1_X_AT	myosin, heavy chain 6, cardiac muscle, alpha; similar to fast myosin heavy chain HCIII; myosin, heavy chain 8, skeletal muscle, perinatal; myosin, heavy chain 1, skeletal muscle, adult; myosin, heavy chain 3, skeletal muscle, embryonic; similar to fast myosin heavy chain HCII; myosin, heavy chain 2, skeletal muscle, adult		

GGA.4217.5.S1_AT	myosin, heavy chain 6, cardiac muscle, alpha; similar to fast myosin heavy chain HCIII; myosin, heavy chain 8, skeletal muscle, perinatal; myosin, heavy chain 1, skeletal muscle, adult; myosin, heavy chain 3, skeletal muscle, embryonic; similar to fast myosin heavy chain HCII; myosin, heavy chain 2, skeletal muscle, adult
GGA.4217.1.S1_A_AT	myosin, heavy chain 6, cardiac muscle, alpha; similar to fast myosin heavy chain HCIII; myosin, heavy chain 8, skeletal muscle, perinatal; myosin, heavy chain 1, skeletal muscle, adult; myosin, heavy chain 3, skeletal muscle, embryonic; similar to fast myosin heavy chain HCII; myosin, heavy chain 2, skeletal muscle, adult
GGA.3685.1.S1_AT	myosin, heavy chain 6, cardiac muscle, alpha; similar to fast myosin heavy chain HCIII; myosin, heavy chain 8, skeletal muscle, perinatal; myosin, heavy chain 1, skeletal muscle, adult; myosin, heavy chain 3, skeletal muscle, embryonic; similar to fast myosin heavy chain HCII; myosin, heavy chain 2, skeletal muscle, adult
GGA.4217.6.S1_X_AT	myosin, heavy chain 6, cardiac muscle, alpha; similar to fast myosin heavy chain HCIII; myosin, heavy chain 8, skeletal muscle, perinatal; myosin, heavy chain 1, skeletal muscle, adult; myosin, heavy chain 3, skeletal muscle, embryonic; similar to fast myosin heavy chain HCII; myosin, heavy chain 2, skeletal muscle, adult
GGA.4217.4.S1_X_AT	myosin, heavy chain 6, cardiac muscle, alpha; similar to fast myosin heavy chain HCIII; myosin, heavy chain 8, skeletal muscle, perinatal; myosin, heavy chain 1, skeletal muscle, adult; myosin, heavy chain 3, skeletal muscle, embryonic; similar to fast myosin heavy chain HCII; myosin, heavy chain 2, skeletal muscle, adult
GGA.841.1.S1_AT	myosin, light chain 2, regulatory, cardiac, slow
GGA.4975.1.S1_A_AT	tropomyosin 3
GGA.823.1.S1_AT	troponin C type 2 (fast)
GGA.2698.1.S1_AT	myosin, light chain 4, alkali; atrial, embryonic
GGA.8433.1.S1_A_AT	phosphatase and actin regulator 1
GGA.4108.4.S1_S_AT	tropomyosin 1 (alpha)
GGA.4108.4.S1_X_AT	tropomyosin 1 (alpha)
GGA.4108.2.S1_A_AT	tropomyosin 1 (alpha)
GGA.4108.5.S1_X_AT	tropomyosin 1 (alpha)
GGA.4108.4.S1_A_AT	tropomyosin 1 (alpha)
GGA.10659.1.S1_S_AT	formin 2
GGA.4843.2.S1_A_AT	actinin, alpha 2; similar to alpha-actinin

**Enrichment Score: 1.7524123155828648**	**SP_PIR_KEYWORDS: skeletal muscle**	**Count: 7**	***p* = 1.53E-07**
GGA.4217.6.S1_X_AT	myosin, heavy chain 6, cardiac muscle, alpha; similar to fast myosin heavy chain HCIII; myosin, heavy chain 8, skeletal muscle, perinatal; myosin, heavy chain 1, skeletal muscle, adult; myosin, heavy chain 3, skeletal muscle, embryonic; similar to fast myosin heavy chain HCII; myosin, heavy chain 2, skeletal muscle, adult		
GGA.3685.1.S1_AT	myosin, heavy chain 6, cardiac muscle, alpha; similar to fast myosin heavy chain HCIII; myosin, heavy chain 8, skeletal muscle, perinatal; myosin, heavy chain 1, skeletal muscle, adult; myosin, heavy chain 3, skeletal muscle, embryonic; similar to fast myosin heavy chain HCII; myosin, heavy chain 2, skeletal muscle, adult		
GGA.4217.5.S1_AT	myosin, heavy chain 6, cardiac muscle, alpha; similar to fast myosin heavy chain HCIII; myosin, heavy chain 8, skeletal muscle, perinatal; myosin, heavy chain 1, skeletal muscle, adult; myosin, heavy chain 3, skeletal muscle, embryonic; similar to fast myosin heavy chain HCII; myosin, heavy chain 2, skeletal muscle, adult		
GGA.4217.5.S1_X_AT	myosin, heavy chain 6, cardiac muscle, alpha; similar to fast myosin heavy chain HCIII; myosin, heavy chain 8, skeletal muscle, perinatal; myosin, heavy chain 1, skeletal muscle, adult; myosin, heavy chain 3, skeletal muscle, embryonic; similar to fast myosin heavy chain HCII; myosin, heavy chain 2, skeletal muscle, adult		
GGA.4217.4.S1_X_AT	myosin, heavy chain 6, cardiac muscle, alpha; similar to fast myosin heavy chain HCIII; myosin, heavy chain 8, skeletal muscle, perinatal; myosin, heavy chain 1, skeletal muscle, adult; myosin, heavy chain 3, skeletal muscle, embryonic; similar to fast myosin heavy chain HCII; myosin, heavy chain 2, skeletal muscle, adult		
GGA.4217.1.S1_A_AT	myosin, heavy chain 6, cardiac muscle, alpha; similar to fast myosin heavy chain HCIII; myosin, heavy chain 8, skeletal muscle, perinatal; myosin, heavy chain 1, skeletal muscle, adult; myosin, heavy chain 3, skeletal muscle, embryonic; similar to fast myosin heavy chain HCII; myosin, heavy chain 2, skeletal muscle, adult		
GGA.4217.2.S1_AT	myosin, heavy chain 6, cardiac muscle, alpha; similar to fast myosin heavy chain HCIII; myosin, heavy chain 8, skeletal muscle, perinatal; myosin, heavy chain 1, skeletal muscle, adult; myosin, heavy chain 3, skeletal muscle, embryonic; similar to fast myosin heavy chain HCII; myosin, heavy chain 2, skeletal muscle, adult		
GGA.750.1.S1_AT	myogenic differentiation 1		
GGA.4835.1.S1_AT	myosin, light chain 1, alkali; skeletal, fast		
GGA.18909.1.S1_S_AT	myosin, light chain 1, alkali; skeletal, fast		
GGA.4975.1.S1_A_AT	tropomyosin 3		
GGA.4108.4.S1_X_AT	tropomyosin 1 (alpha)		
GGA.4108.4.S1_A_AT	tropomyosin 1 (alpha)		

GGA.4108.4.S1_S_AT	tropomyosin 1 (alpha)
GGA.4108.2.S1_A_AT	tropomyosin 1 (alpha)
GGA.4108.5.S1_X_AT	tropomyosin 1 (alpha)
GGA.4843.2.S1_A_AT	actinin, alpha 2; similar to alpha-actinin
GGA.4090.6.S1_A_AT	troponin T type 3 (skeletal, fast)
GGA.4090.1.S1_A_AT	troponin T type 3 (skeletal, fast)

Supplementary Table 2. List of genes expressed during formation of fibrin-gel based TECs.

Total probe sets with significant ≥2-fold change from 2D to fibrin gel (restrained) = 1173 (q < 0.01)

Top annotation clusters upregulated from 2D to fibrin gel (restrained) (from 559 probe sets)

**Enrichment Score: 5.6531672323796025**	**SP_PIR_KEYWORDS: disulfide bond**	**Count: 32**	***p* = 1.06E-08**
GGA.5002.1.S1_AT	midkine (neurite growth-promoting factor 2)		
GGA.2679.1.S1_AT	fibulin 1		
GGA.4248.1.S1_AT	lipoprotein lipase		
GGA.1171.1.S1_AT	lymphocyte antigen 6 complex, locus E		
GGA.16844.2.S1_AT	collagen, type III, alpha 1		
GGA.10960.1.S1_AT	collectin sub-family member 12		
GGA.2551.2.S1_S_AT	lactotransferrin		
GGAAFFX.22381.3.S1_AT	ankyrin repeat and kinase domain containing 1		
GGA.5058.1.S1_S_AT	cystatin C		
GGA.4345.1.S1_AT	secreted frizzled-related protein 2		
GGA.110.1.S3_AT	frizzled homolog 7 (Drosophila)		
GGA.481.1.S1_AT	fibromodulin		
GGA.822.1.S1_A_AT	elastin (supravalvular aortic stenosis, Williams-Beuren syndrome)		
GGA.156.2.S1_A_AT	neuroblastoma, suppression of tumorigenicity 1		
GGA.13301.1.S1_AT	glycerophosphodiester phosphodiesterase domain containing 5; similar to glycerophosphodiester phosphodiesterase 2		
GGA.1148.1.S1_AT	ST6 beta-galactosamide alpha-2,6-sialyltranferase 1		
GGA.1784.1.S1_AT	integrin, alpha 8		
GGA.3745.1.S1_AT	carboxypeptidase Z; similar to carboxypeptidase Z		
GGA.2679.2.S1_A_AT	fibulin 1		
GGA.156.1.S1_AT	neuroblastoma, suppression of tumorigenicity 1		
GGA.701.1.S1_S_AT	leukocyte ribonuclease A-1; leukocyte ribonuclease A-2		
GGA.3745.1.S2_AT	carboxypeptidase Z; similar to carboxypeptidase Z		
GGA.3332.1.S1_AT	matrix-remodelling associated 8		
GGA.4194.1.S1_AT	osteoglycin		
GGA.2558.1.S1_A_AT	similar to collagen XIV; collagen, type XIV, alpha 1 (undulin); similar to collagen, type XIV, alpha 1 (undulin)		
GGA.15998.1.S1_AT	collectin sub-family member 12		
GGA.16844.2.S1_S_AT	collagen, type III, alpha 1		
GGA.817.1.S1_AT	plasminogen activator, urokinase		
GGAAFFX.22381.3.S1_S_AT	ankyrin repeat and kinase domain containing 1		
GGA.312.1.S1_AT	frizzled homolog 2 (Drosophila)		
GGA.3573.2.S1_A_AT	dickkopf homolog 3 (Xenopus laevis)		
GGA.1479.2.S1_A_AT	pleiotrophin		
GGA.1784.1.S2_AT	integrin, alpha 8		
GGA.483.1.S1_AT	cathepsin K		
GGA.1763.1.S1_AT	WAP four-disulfide core domain 1; similar to PS20 protein		
GGAAFFX.25534.1.S1_S_AT	collagen, type III, alpha 1		
GGA.739.1.S1_AT	quiescence-specific protein		
GGA.1148.1.S2_AT	ST6 beta-galactosamide alpha-2,6-sialyltranferase 1		
GGA.1479.1.S1_AT	pleiotrophin		
GGA.98.2.S1_A_AT	toll-like receptor 2		
GGA.729.1.S1_AT	mature avidin; similar to Avidin-related protein 3		
GGA.595.1.S1_AT	NEL-like 2 (chicken)		
GGA.2551.2.S1_A_AT	lactotransferrin		
GGAAFFX.21769.1.S1_S_AT	lipoprotein lipase		

**Enrichment Score: 5.351504372199212**	**GOTERM_CC_FAT: 0044421~extracellular region part**	**Count: 28**	***p* = 1.32E-08**
GGA.5002.1.S1_AT	midkine (neurite growth-promoting factor 2)		
GGA.4248.1.S1_AT	lipoprotein lipase		
GGAAFFX.11429.1.S1_S_AT	matrix metallopeptidase 7 (matrilysin, uterine)		
GGA.501.1.S1_AT	matrix metallopeptidase 27		
GGA.2679.1.S1_AT	fibulin 1		
GGA.13484.1.S1_AT	interleukin 15		
GGA.16844.2.S1_AT	collagen, type III, alpha 1		
GGA.19049.1.S1_AT	interleukin 18 (interferon-gamma-inducing factor)		
GGA.481.1.S1_AT	fibromodulin		
GGA.822.1.S1_A_AT	elastin (supravalvular aortic stenosis, Williams-Beuren syndrome)		
GGAAFFX.26374.1.S1_AT	TIMP metallopeptidase inhibitor 4		
GGA.12614.2.S1_S_AT	similar to TL1A; tumor necrosis factor (ligand) superfamily, member 15		

GGA.690.1.S1_AT	lymphocyte antigen 86
GGA.496.1.S1_AT	reelin
GGA.12614.1.S1_AT	similar to TL1A; tumor necrosis factor (ligand) superfamily, member 15
GGA.3745.1.S1_AT	carboxypeptidase Z; similar to carboxypeptidase Z
GGA.2679.2.S1_A_AT	fibulin 1
GGAAFFX.24566.1.S1_AT	laminin, alpha 4
GGA.398.1.S1_AT	matrix metalloproteinase-13
GGAAFFX.25194.1.S1_AT	angiopoietin-like 5
GGA.16572.1.S1_AT	arginyl aminopeptidase (aminopeptidase B)
GGAAFFX.1557.1.S1_AT	glypican 1; similar to Glypican-1 precursor (Heparan sulfate proteoglycan core protein)
GGA.3745.1.S2_AT	carboxypeptidase Z; similar to carboxypeptidase Z
GGAAFFX.2012.1.S1_AT	angiopoietin-like 7
GGAAFFX.9643.1.S1_AT	proprotein convertase subtilisin/kexin type 5
GGA.4194.1.S1_AT	osteoglycin
GGA.2558.1.S1_A_AT	similar to collagen XIV; collagen, type XIV, alpha 1 (undulin); similar to collagen, type XIV, alpha 1 (undulin)
GGAAFFX.9641.1.S1_AT	proprotein convertase subtilisin/kexin type 5
GGA.16844.2.S1_S_AT	collagen, type III, alpha 1
GGA.1768.1.S1_AT	mannosyl (beta-1,4-)-glycoprotein beta-1,4-N-acetylglucosaminyltransferase
GGA.12800.1.S1_AT	matrix metallopeptidase 3 (stromelysin 1, progelatinase)
GGA.3219.1.S1_AT	c-fos induced growth factor (vascular endothelial growth factor D)
GGAAFFX.25534.1.S1_S_AT	collagen, type III, alpha 1
GGA.1776.1.S1_AT	similar to nidogen 1; entactin; similar to nidogen; hypothetical protein LOC769017; nidogen 1
GGAAFFX.21769.1.S1_S_AT	lipoprotein lipase

**Enrichment Score: 2.1564476319346078**	**GOTERM_MF_FAT: 0004364~glutathione transferase activity**	**Count: 4**	***p* = 7.13E-04**
GGA.788.1.S1_AT	glutathione transferase		
GGA.8359.1.S1_AT	glutathione S-transferase alpha 3		
GGA.12514.1.S1_A_AT	glutathione S-transferase class-alpha		
GGA.2263.1.S1_S_AT	glutathione S-transferase class-alpha		

**Enrichment Score: 1.713496895347525**	**GOTERM_BP_FAT: 0006955~immune response**	**Count: 11**	***p* = 0.0023651**
GGA.8774.1.S1_S_AT	tumor necrosis factor (ligand) superfamily, member 10		
GGA.13484.1.S1_AT	interleukin 15		
GGA.10960.1.S1_AT	collectin sub-family member 12		
GGA.100.1.S1_A_AT	Fas (TNF receptor superfamily, member 6)		
GGA.15998.1.S1_AT	collectin sub-family member 12		
GGA.19049.1.S1_AT	interleukin 18 (interferon-gamma-inducing factor)		
GGA.11016.1.S1_AT	B locus M alpha chain 1		
GGA.12614.2.S1_S_AT	similar to TL1A; tumor necrosis factor (ligand) superfamily, member 15		
GGA.8774.1.S2_S_AT	tumor necrosis factor (ligand) superfamily, member 10		
GGA.8408.2.S1_S_AT	tumor necrosis factor (ligand) superfamily, member 10		
GGA.9513.1.S2_AT	chemokine (C-X-C motif) ligand 12 (stromal cell-derived factor 1)		
GGA.690.1.S1_AT	lymphocyte antigen 86		
GGAAFFX.21581.1.S1_S_AT	chemokine (C-X-C motif) ligand 14		
GGA.12614.1.S1_AT	similar to TL1A; tumor necrosis factor (ligand) superfamily, member 15		
GGA.8774.1.S2_AT	tumor necrosis factor (ligand) superfamily, member 10		
GGA.10683.1.S1_AT	Fas (TNF receptor superfamily, member 6)		
GGA.98.2.S1_A_AT	toll-like receptor 2		

Top annotation clusters downregulated from 2D to fibrin gel (restrained) (from 614 probe sets)

**Enrichment Score: 2.26384407935878**	**GOTERM_CC_FAT: 0043292~contractile fiber**	**Count: 10**	***p* = 6.34E-07**
GGA.1137.1.S2_AT	myosin binding protein C, cardiac		
GGA.4217.3.S1_X_AT	myosin, heavy chain 6, cardiac muscle, alpha; similar to fast myosin heavy chain HCIII; myosin, heavy chain 8, skeletal muscle, perinatal; myosin, heavy chain 1, skeletal muscle, adult; myosin, heavy chain 3, skeletal muscle, embryonic; similar to fast myosin heavy chain HCII; myosin, heavy chain 2, skeletal muscle, adult		
GGA.4217.3.S1_AT	myosin, heavy chain 6, cardiac muscle, alpha; similar to fast myosin heavy chain HCIII; myosin, heavy chain 8, skeletal muscle, perinatal; myosin, heavy chain 1, skeletal muscle, adult; myosin, heavy chain 3, skeletal muscle, embryonic; similar to fast myosin heavy chain HCII; myosin, heavy chain 2, skeletal muscle, adult		
GGA.5751.1.S1_AT	ankyrin repeat domain 1 (cardiac muscle)		
GGA.5751.1.S2_AT	ankyrin repeat domain 1 (cardiac muscle)		
GGA.698.1.S1_AT	vinculin		
GGA.698.1.S2_AT	vinculin		

GGA.4870.3.S1_A_AT	actin, beta-like 2; actin, alpha, cardiac muscle 1; actin, alpha 1, skeletal muscle
GGA.5962.1.S1_AT	actin, beta-like 2; actin, alpha, cardiac muscle 1; actin, alpha 1, skeletal muscle
GGA.4530.1.S1_AT	actin, alpha 2, smooth muscle, aorta; actin, gamma 2, smooth muscle, enteric
GGA.2617.1.S1_AT	myosin, heavy polypeptide 6, cardiac muscle, alpha
GGA.4108.1.S2_AT	tropomyosin 1 (alpha)
GGA.4108.2.S1_A_AT	tropomyosin 1 (alpha)
GGA.4108.5.S1_X_AT	tropomyosin 1 (alpha)
GGA.718.1.S1_AT	dystrophin
GGAAFFX.12773.1.S1_S_AT	actinin, alpha 1

**Enrichment Score: 2.1780253575150805**	**GOTERM_BP_FAT: 0007155~cell adhesion**	**Count: 17**	***p* = 0.0024689**
GGA.1137.1.S2_AT	myosin binding protein C, cardiac		
GGA.2734.1.S2_AT	activated leukocyte cell adhesion molecule		
GGA.2734.1.S1_AT	activated leukocyte cell adhesion molecule		
GGAAFFX.20275.1.S1_S_AT	activated leukocyte cell adhesion molecule		
GGA.888.2.S1_A_AT	collagen, type VI, alpha 3		
GGA.3754.2.S1_AT	hairy and enhancer of split 1, (Drosophila)		
GGA.3928.1.S1_AT	hyaluronan and proteoglycan link protein 1		
GGA.10389.1.S1_S_AT	integrin, alpha 11		
GGA.3551.1.S1_AT	secreted phosphoprotein 1 (osteopontin, bone sialoprotein I, early T-lymphocyte activation 1)		
GGA.1193.1.S2_AT	protein tyrosine phosphatase, receptor type, C		
GGA.3852.1.S2_S_AT	ras homolog gene family, member B		
GGA.3852.1.S1_AT	ras homolog gene family, member B		
GGAAFFX.22987.1.S1_AT	thrombospondin 1		
GGA.3448.1.S1_AT	thrombospondin 1		
GGA.10300.1.S1_S_AT	thrombospondin 1		
GGA.9480.1.S1_AT	FAT tumor suppressor homolog 1 (Drosophila)		
GGAAFFX.8655.1.S1_S_AT	FAT tumor suppressor homolog 1 (Drosophila)		
GGA.698.1.S1_AT	vinculin		
GGA.698.1.S2_AT	vinculin		
GGA.4974.1.S2_AT	versican		
GGA.2967.1.S2_AT	integrin, alpha 6; similar to integrin alpha 6 subunit		
GGA.2967.1.S1_AT	integrin, alpha 6; similar to integrin alpha 6 subunit		
GGA.10439.1.S1_AT	fibronectin 1		
GGA.9772.1.S1_S_AT	fibronectin 1		
GGA.13391.1.S1_AT	similar to collagen, type XXVII, alpha 1		
GGA.3348.2.S1_S_AT	protocadherin 9		

Total probe sets with significant ≥2-fold change from fibrin gel (restrained) to 3D fibrin TEC = 1684 (q < 0.01)

Top annotation clusters upregulated from fibrin gel (restrained) to 3D fibrin TEC (from 821 probe sets)

**Enrichment Score: 2.363300696455806**	**GOTERM_CC_FAT: 0005576~extracellular region**	**Count: 33**	***p* = 6.46E-04**
GGA.501.1.S1_AT	matrix metallopeptidase 27		
GGAAFFX.11429.1.S1_S_AT	matrix metallopeptidase 7 (matrilysin, uterine)		
GGA.13484.1.S1_AT	interleukin 15		
GGA.10439.1.S1_AT	fibronectin 1		
GGA.520.2.S1_A_AT	activin beta B		
GGA.520.1.S1_AT	activin beta B		
GGAAFFX.22982.1.S1_AT	transforming growth factor, beta 2		
GGA.4719.1.S1_A_AT	apolipoprotein A-I		
GGA.3982.1.S1_AT	inhibin, beta A		
GGA.5162.1.S1_AT	coagulation factor V (proaccelerin, labile factor)		
GGA.652.1.S1_AT	neurohypophyseal)		
GGA.12454.1.S1_AT	relaxin 3		
GGAAFFX.26374.1.S1_AT	TIMP metallopeptidase inhibitor 4		
GGA.12614.2.S1_S_AT	similar to TL1A; tumor necrosis factor (ligand) superfamily, member 15		
GGA.1812.1.S1_AT	tumor necrosis factor receptor superfamily, member 11b (osteoprotegerin)		
GGA.12006.1.S1_AT	adrenomedullin		
GGA.19330.1.S1_AT	similar to collagen, type XXVII, alpha 1		
GGA.12614.1.S1_AT	similar to TL1A; tumor necrosis factor (ligand) superfamily, member 15		
GGA.3551.1.S1_AT	secreted phosphoprotein 1 (osteopontin, bone sialoprotein I, early T-lymphocyte activation 1)		
GGA.909.1.S1_AT	slit homolog 2 (Drosophila); slit-2		
GGA.701.1.S1_S_AT	leukocyte ribonuclease A-1; leukocyte ribonuclease A-2		

GGAAFFX.20296.1.S1_S_AT	similar to similar to 60 kDa heat shock protein, mitochondrial precursor (Hsp60) (60 kDa chaperonin) (CPN60) (Heat shock protein 60) (HSP-60) (Mitochondrial matrix protein P1) (HSP-65); heat shock 60kDa protein 1 (chaperonin); similar to 60 kDa heat shock protein, mitochondrial precursor (Hsp60) (60 kDa chaperonin) (CPN60) (Heat shock protein 60) (HSP-60) (Mitochondrial matrix protein P1) (P60 lymphocyte protein) (HuCHA60)
GGA.305.1.S1_AT	wingless-type MMTV integration site family, member 4
GGA.398.1.S1_AT	matrix metalloproteinase-13
GGAAFFX.1557.1.S1_AT	glypican 1; similar to Glypican-1 precursor (Heparan sulfate proteoglycan core protein)
GGA.3151.1.S1_AT	interferon (alpha, beta and omega) receptor 2
GGAAFFX.10059.1.S1_AT	ADAM metallopeptidase with thrombospondin type 1 motif, 5 (aggrecanase-2)
GGAAFFX.21780.2.S1_S_AT	glucagon
GGAAFFX.20296.1.S1_AT	similar to similar to 60 kDa heat shock protein, mitochondrial precursor (Hsp60) (60 kDa chaperonin) (CPN60) (Heat shock protein 60) (HSP-60) (Mitochondrial matrix protein P1) (HSP-65); heat shock 60kDa protein 1 (chaperonin); similar to 60 kDa heat shock protein, mitochondrial precursor (Hsp60) (60 kDa chaperonin) (CPN60) (Heat shock protein 60) (HSP-60) (Mitochondrial matrix protein P1) (P60 lymphocyte protein) (HuCHA60)
GGA.4719.1.S1_S_AT	apolipoprotein A-I
GGA.9772.1.S1_S_AT	fibronectin 1
GGA.817.1.S1_AT	plasminogen activator, urokinase
GGA.1768.1.S1_AT	mannosyl (beta-1,4-)-glycoprotein beta-1,4-N-acetylglucosaminyltransferase
GGA.7960.1.S1_AT	wingless-type MMTV integration site family, member 5B
GGA.198.1.S1_AT	matrix metallopeptidase 9 (gelatinase B, 92kDa gelatinase, 92kDa type IV collagenase)
GGA.13487.1.A1_AT	fibronectin 1
GGA.514.1.S1_AT	similar to Coagulation factor X precursor (Stuart factor) (Virus-activating protease) (VAP); coagulation factor X
GGA.537.2.S1_A_AT	vascular endothelial growth factor A
GGA.701.1.S1_AT	leukocyte ribonuclease A-1; leukocyte ribonuclease A-2
GGA.3982.1.S2_AT	inhibin, beta A
GGA.9772.1.S1_AT	fibronectin 1
GGA.826.1.S1_S_AT	interleukin 8

**Enrichment Score: 1.8152848525498053**	**GOTERM_BP_FAT: 0048565~gut development**	**Count: 5**	***p* = 0.0031992**
GGA.10829.1.S1_S_AT	endothelin receptor type B		
GGA.3306.1.S1_S_AT	endothelin receptor type B		
GGA.764.1.S1_AT	activin A receptor, type IIB		
GGA.3297.1.S1_AT	GLI-Kruppel family member GLI3 (Greig cephalopolysyndactyly syndrome)		
GGA.19087.1.S1_AT	v-ral simian leukemia viral oncogene homolog B (ras related; GTP binding protein); GLI family zinc finger 2		
GGA.9866.2.S1_A_AT	v-ral simian leukemia viral oncogene homolog B (ras related; GTP binding protein); GLI family zinc finger 2		
GGA.9606.1.S1_S_AT	transcription factor 7-like 2 (T-cell specific, HMG-box)		

**Enrichment Score: 1.7783257033515862**	**GOTERM_BP_FAT: :0009890~negative regulation of biosynthetic process**	**Count: 14**	***p* = 0.0027671**
GGA.16905.1.S1_AT	similar to nuclear receptor coactivator 2; nuclear receptor coactivator 2		
GGA.520.2.S1_A_AT	activin beta B		
GGA.520.1.S1_AT	activin beta B		
GGA.137.1.S1_AT	endothelin receptor type A		
GGA.1039.1.S1_AT	integrin, beta 3 (platelet glycoprotein IIIa, antigen CD61)		
GGA.3297.1.S1_AT	GLI-Kruppel family member GLI3 (Greig cephalopolysyndactyly syndrome)		
GGAAFFX.12452.1.S1_S_AT	Kruppel-like factor 11		
GGA.2815.1.S1_AT	human immunodeficiency virus type I enhancer binding protein 1		
GGA.9866.2.S1_A_AT	v-ral simian leukemia viral oncogene homolog B (ras related; GTP binding protein); GLI family zinc finger 2		
GGA.17558.1.S1_S_AT	similar to nuclear receptor coactivator 2; nuclear receptor coactivator 2		
GGA.892.1.S1_AT	inhibitor of DNA binding 1, dominant negative helix-loop-helix protein		
GGA.126.1.S1_AT	transducin-like enhancer of split 4 (E(sp1) homolog, Drosophila)		
GGAAFFX.8053.1.S1_AT	jumonji, AT rich interactive domain 2		
GGA.890.1.S1_AT	SRY (sex determining region Y)-box 9		
GGA.19087.1.S1_AT	v-ral simian leukemia viral oncogene homolog B (ras related; GTP binding protein); GLI family zinc finger 2		
GGA.701.1.S1_S_AT	leukocyte ribonuclease A-1; leukocyte ribonuclease A-2		
GGA.701.1.S1_AT	leukocyte ribonuclease A-1; leukocyte ribonuclease A-2		
GGA.768.1.S1_AT	TGFB-induced factor homeobox 1		

Top annotation clusters downregulated from fibrin gel (restrained) to 3D fibrin TEC (from 863 probe sets)

Enrichment Score: 8.054421413715191	GOTERM_BP_FAT: 0007049~cell cycle	Count: 28	*p* = 2.84E-10
GGAAFFX.4223.1.S1_S_AT	kinesin family member 11; hypothetical LOC426105; similar to similar to kinesin like protein		
GGAAFFX.11417.1.S1_AT	kinesin family member 11; hypothetical LOC426105; similar to similar to kinesin like protein		
GGA.4547.2.S1_A_AT	myosin, heavy chain 10, non-muscle; myosin, heavy chain 11, smooth muscle; similar to Myosin-11 (Myosin heavy chain, gizzard smooth muscle); similar to myosin, heavy chain 9, non-muscle		
GGAAFFX.4223.1.S1_S_AT	kinesin family member 11; hypothetical LOC426105; similar to similar to kinesin like protein		
GGA.4547.1.S1_AT	myosin, heavy chain 10, non-muscle; myosin, heavy chain 11, smooth muscle; similar to Myosin-11 (Myosin heavy chain, gizzard smooth muscle); similar to myosin, heavy chain 9, non-muscle		
GGA.7132.1.S1_AT	CDC28 protein kinase regulatory subunit 1B		
GGAAFFX.5404.1.S1_S_AT	nucleolar and spindle associated protein 1		
GGAAFFX.12701.1.S1_AT	polo-like kinase 1 (Drosophila)		
GGA.8462.1.S1_AT	BUB1 budding uninhibited by benzimidazoles 1 homolog beta (yeast)		
GGAAFFX.13010.1.S1_AT	NUF2, NDC80 kinetochore complex component, homolog (S. cerevisiae)		
GGA.9459.1.S1_S_AT	NUF2, NDC80 kinetochore complex component, homolog (S. cerevisiae)		
GGA.15741.1.S1_AT	growth arrest-specific 2		
GGA.4516.2.S1_S_AT	septin 11		
GGA.3200.1.S1_S_AT	structural maintenance of chromosomes 4		
GGAAFFX.11513.1.S1_AT	cyclin E2		
GGAAFFX.11513.1.S1_S_AT	cyclin E2		
GGA.10660.2.S1_AT	polo-like kinase 2 (Drosophila)		
GGAAFFX.24493.1.S1_AT	polo-like kinase 2 (Drosophila)		
GGA.10660.1.S1_S_AT	polo-like kinase 2 (Drosophila)		
GGA.1984.2.A1_A_AT	MAD2 mitotic arrest deficient-like 1 (yeast)		
GGA.1251.2.S1_A_AT	inner centromere protein antigens 135/155kDa		
GGA.4066.1.S1_AT	structural maintenance of chromosomes 2		
GGA.4493.1.S2_AT	breast cancer 1, early onset		
GGA.4493.1.S1_AT	breast cancer 1, early onset		
GGA.14834.1.S1_S_AT	breast cancer 1, early onset		
GGAAFFX.6135.1.S1_AT	centromere protein F, 350/400ka (mitosin)		
GGA.3705.1.S1_AT	centromere protein F, 350/400ka (mitosin)		
GGAAFFX.20508.1.S1_S_AT	centromere protein F, 350/400ka (mitosin)		
GGA.292.1.S1_AT	centromere protein F, 350/400ka (mitosin)		
GGAAFFX.6125.1.S1_AT	centromere protein F, 350/400ka (mitosin)		
GGA.1500.1.S1_AT	septin 6		
GGAAFFX.12568.1.S1_AT	coiled-coil domain containing 5 (spindle associated)		
GGAAFFX.1153.1.S1_S_AT	coiled-coil domain containing 5 (spindle associated)		
GGAAFFX.11558.1.S1_S_AT	nudE nuclear distribution gene E homolog 1 (A. nidulans)		
GGA.1551.2.S1_A_AT	baculoviral IAP repeat-containing 5 (survivin)		
GGA.7355.1.S1_AT	MIS12, MIND kinetochore complex component, homolog (yeast)		
GGA.9350.1.S1_S_AT	NDC80 homolog, kinetochore complex component (S. cerevisiae)		
GGAAFFX.11749.1.S1_AT	NDC80 homolog, kinetochore complex component (S. cerevisiae)		
GGA.687.1.S1_AT	cyclin B3		
GGA.3146.1.S1_AT	cyclin B2		
GGA.726.2.S1_A_AT	cell division cycle 2, G1 to S and G2 to M		
GGA.4129.1.S1_AT	cyclin A2		
GGAAFFX.11402.1.S1_AT	NIMA (never in mitosis gene a)-related kinase 2		

Enrichment Score: 4.6972626825342685	GOTERM_CC_FAT: 0000775~chromosome, centromeric region	Count: 12	*p* = 1.31E-07
GGA.1551.2.S1_A_AT	baculoviral IAP repeat-containing 5 (survivin)		
GGAAFFX.8595.2.S1_S_AT	centromere protein N; similar to centromere protein N		
GGAAFFX.8595.2.S1_AT	centromere protein N; similar to centromere protein N		
GGA.7355.1.S1_AT	MIS12, MIND kinetochore complex component, homolog (yeast)		
GGA.1251.2.S1_A_AT	inner centromere protein antigens 135/155kDa		
GGA.31.1.S1_AT	centromere protein I		
GGA.9350.1.S1_S_AT	NDC80 homolog, kinetochore complex component (S. cerevisiae)		
GGAAFFX.11749.1.S1_AT	NDC80 homolog, kinetochore complex component (S. cerevisiae)		
GGAAFFX.12087.1.S1_S_AT	centromere protein L		
GGA.944.1.S1_AT	centromere protein H		
GGAAFFX.6135.1.S1_AT	centromere protein F, 350/400ka (mitosin)		
GGAAFFX.6125.1.S1_AT	centromere protein F, 350/400ka (mitosin)		
GGAAFFX.20508.1.S1_S_AT	centromere protein F, 350/400ka (mitosin)		
GGA.292.1.S1_AT	centromere protein F, 350/400ka (mitosin)		
GGA.3705.1.S1_AT	centromere protein F, 350/400ka (mitosin)		
GGA.8462.1.S1_AT	BUB1 budding uninhibited by benzimidazoles 1 homolog beta (yeast)		
GGA.9459.1.S1_S_AT	NUF2, NDC80 kinetochore complex component, homolog (S. cerevisiae)		
GGAAFFX.13010.1.S1_AT	NUF2, NDC80 kinetochore complex component, homolog (S. cerevisiae)		

GGA.7180.1.S1_S_AT	BUB1 budding uninhibited by benzimidazoles 1 homolog (yeast)

**Enrichment Score: 2.958318190615208**	**GOTERM_CC_FAT: 0015630~microtubule cytoskeleton**	**Count: 20**	***p* = 3.56E-06**
GGAAFFX.11417.1.S1_AT	kinesin family member 11; hypothetical LOC426105; similar to similar to kinesin like protein		
GGAAFFX.4223.1.S1_S_AT	kinesin family member 11; hypothetical LOC426105; similar to similar to kinesin like protein		
GGA.8347.1.S1_AT	tubulin, alpha 4a		
GGA.4547.1.S1_AT	myosin, heavy chain 10, non-muscle; myosin, heavy chain 11, smooth muscle; similar to Myosin-11 (Myosin heavy chain, gizzard smooth muscle); similar to myosin, heavy chain 9, non-muscle		
GGA.4547.2.S1_A_AT	myosin, heavy chain 10, non-muscle; myosin, heavy chain 11, smooth muscle; similar to Myosin-11 (Myosin heavy chain, gizzard smooth muscle); similar to myosin, heavy chain 9, non-muscle		
GGA.1251.2.S1_A_AT	inner centromere protein antigens 135/155kDa		
GGAAFFX.5404.1.S1_S_AT	nucleolar and spindle associated protein 1		
GGAAFFX.5077.1.S1_S_AT	kinesin family member 23		
GGAAFFX.13032.1.S1_S_AT	kinesin family member 23		
GGA.13824.1.S1_AT	dynein, axonemal, heavy chain 10		
GGAAFFX.6135.1.S1_AT	centromere protein F, 350/400ka (mitosin)		
GGAAFFX.6125.1.S1_AT	centromere protein F, 350/400ka (mitosin)		
GGA.292.1.S1_AT	centromere protein F, 350/400ka (mitosin)		
GGA.3705.1.S1_AT	centromere protein F, 350/400ka (mitosin)		
GGAAFFX.20508.1.S1_S_AT	centromere protein F, 350/400ka (mitosin)		
GGA.9459.1.S1_S_AT	NUF2, NDC80 kinetochore complex component, homolog (S. cerevisiae)		
GGAAFFX.13010.1.S1_AT	NUF2, NDC80 kinetochore complex component, homolog (S. cerevisiae)		
GGAAFFX.12568.1.S1_AT	coiled-coil domain containing 5 (spindle associated)		
GGAAFFX.1153.1.S1_S_AT	coiled-coil domain containing 5 (spindle associated)		
GGAAFFX.11558.1.S1_S_AT	nudE nuclear distribution gene E homolog 1 (A. nidulans)		
GGAAFFX.12208.1.S1_S_AT	kinesin family member 20A		
GGA.1551.2.S1_A_AT	baculoviral IAP repeat-containing 5 (survivin)		
GGAAFFX.12873.1.S1_AT	spermatid perinuclear RNA binding protein		
GGAAFFX.11749.1.S1_AT	NDC80 homolog, kinetochore complex component (S. cerevisiae)		
GGA.9350.1.S1_S_AT	NDC80 homolog, kinetochore complex component (S. cerevisiae)		
GGA.3146.1.S1_AT	cyclin B2		
GGA.159.1.S1_AT	similar to midline-1; midline 1		
GGA.2844.2.S1_S_AT	stathmin 1		
GGA.4358.1.S1_A_AT	kinesin family member 4A		
GGA.4358.2.S1_A_AT	kinesin family member 4A		
GGA.4358.1.S1_AT	kinesin family member 4A		
GGAAFFX.11402.1.S1_AT	NIMA (never in mitosis gene a)-related kinase 2		

Total probe sets with significant ≥2-fold change from 3D fibrin TEC to mature fibrin TEC = 7037 (q < 0.01)

Top annotation clusters upregulated from 3D fibrin TEC to mature fibrin TEC (from 1758 probe sets)

**Enrichment Score: 13.01414408881739**	**GOTERM_CC_FAT: 0005576~extracellular region**	**Count: 92**	***p* = 8.67E-17**
GGA.3448.1.S1_AT	thrombospondin 1		
GGA.8352.1.S1_AT	similar to merosin; similar to laminin alpha 2 chain; similar to laminin alpha 2; laminin, alpha 2 (merosin, congenital muscular dystrophy); similar to mKIAA4087 protein		
GGA.739.1.S1_AT	quiescence-specific protein		
GGAAFFX.9647.2.S1_AT	proprotein convertase subtilisin/kexin type 5		
GGAAFFX.20104.1.S1_S_AT	periostin, osteoblast specific factor		
GGA.5128.1.S1_AT	chemokine (C-C motif) ligand 20		
GGA.3551.1.S1_AT	secreted phosphoprotein 1 (osteopontin, bone sialoprotein I, early T-lymphocyte activation 1)		
GGA.148.1.S1_AT	quiescin Q6 sulfhydryl oxidase 1		
GGA.155.1.S1_S_AT	lectin, galactoside-binding, soluble, 3		
GGA.6271.1.S1_AT	hormone/ beta-melanocyte stimulating hormone/ beta-endorphin)		
GGA.3484.1.S1_AT	antigen p97 (melanoma associated) identified by monoclonal antibodies 133.2 and 96.5		
GGA.3903.1.S1_AT	cadherin 13, H-cadherin (heart)		
GGA.150.2.S1_A_AT	receptor)		
GGA.19956.1.S1_S_AT	chitinase, acidic		
GGA.7471.2.A1_S_AT	heparan sulfate proteoglycan 2		
GGAAFFX.20952.1.A1_S_AT	collagen, type II, alpha 1		
GGAAFFX.4556.1.S1_AT	proline rich Gla (G-carboxyglutamic acid) 3 (transmembrane)		
GGA.2558.1.S1_A_AT	similar to collagen XIV; collagen, type XIV, alpha 1 (undulin); similar to collagen, type XIV, alpha 1 (undulin)		
GGA.653.1.S1_AT	tenascin XB		
GGA.4955.1.S1_AT	frizzled-related protein		
GGAAFFX.25487.1.S1_AT	avidin related protein 2		

GGAAFFX.25090.2.S1_AT	collagen, type IV, alpha 2
GGA.496.1.S1_AT	reelin
GGA.3928.1.S1_AT	hyaluronan and proteoglycan link protein 1
GGAAFFX.24460.1.S1_AT	complement C4
GGA.2613.1.S1_S_AT	secreted protein, acidic, cysteine-rich (osteonectin)
GGA.2156.2.S1_AT	slit homolog 1 (Drosophila)
GGA.5710.2.S1_A_AT	HtrA serine peptidase 3
GGA.9088.1.S1_AT	somatostatin II
GGA.648.1.S2_AT	fibroblast growth factor 1 (acidic)
GGA.4990.1.S1_AT	collagen, type IX, alpha 1
GGAAFFX.10781.1.S1_AT	collagen, type IV, alpha 1
GGAAFFX.2012.1.S1_AT	angiopoietin-like 7
GGAAFFX.10135.1.S1_AT	CD109 molecule
GGA.6664.1.A1_X_AT	gallinacin 1
GGA.75.1.A1_AT	thrombospondin 3
GGA.636.1.S1_AT	integrin-binding sialoprotein (bone sialoprotein, bone sialoprotein II)
GGA.16844.1.S1_A_AT	collagen, type III, alpha 1
GGAAFFX.2973.1.S1_AT	collagen, type IV, alpha 3
GGAAFFX.23500.1.S1_AT	inhibin, alpha
GGA.4451.1.S1_AT	gelsolin
GGA.6218.1.S1_AT	microfibrillar associated protein 5
GGAAFFX.12486.1.S1_S_AT	SCO-spondin homolog (Bos taurus)
GGA.822.1.S1_A_AT	elastin (supravalvular aortic stenosis, Williams-Beuren syndrome)
GGA.690.1.S1_AT	lymphocyte antigen 86
GGA.481.1.S1_AT	fibromodulin
GGA.866.1.S1_AT	collagen, type XVII, alpha 1
GGA.1812.1.S1_AT	tumor necrosis factor receptor superfamily, member 11b (osteoprotegerin)
GGA.729.1.S1_AT	mature avidin; similar to Avidin-related protein 3
GGA.3615.1.S1_AT	follistatin
GGA.473.1.S1_AT	Indian hedgehog homolog (Drosophila)
GGAAFFX.7243.1.S1_AT	netrin 4
GGA.4821.1.S1_AT	collagen, type XVIII, alpha 1
GGA.2805.1.S1_AT	coagulation factor C homolog, cochlin (Limulus polyphemus)
GGAAFFX.7996.1.S1_AT	collagen, type XV, alpha 1
GGAAFFX.12454.1.S1_S_AT	palmitoyl-protein thioesterase 1 (ceroid-lipofuscinosis, neuronal 1, infantile)
GGA.2587.1.S1_AT	similar to cellular proto-oncogene protein Nov; nephroblastoma overexpressed gene
GGA.5073.1.S1_AT	similar to Trappin-6
GGAAFFX.22067.1.S1_X_AT	WAP four-disulfide core domain 8
GGAAFFX.11829.1.S1_S_AT	chromosome 6 open reading frame 120
GGAAFFX.9712.2.S1_S_AT	collagen, type VIII, alpha 1
GGAAFFX.9042.3.S1_S_AT	similar to alpha-2-macroglobulin
GGA.4292.1.S1_AT	laminin, beta 2 (laminin S)
GGA.16392.1.S1_AT	collagen, type XI, alpha 1
GGA.9962.1.S1_AT	ectonucleoside triphosphate diphosphohydrolase 1
GGAAFFX.26374.1.S1_AT	TIMP metallopeptidase inhibitor 4
GGA.28.1.S1_AT	nodal homolog (mouse)
GGAAFFX.1490.1.S1_AT	agouti related protein homolog (mouse)
GGA.3573.2.S1_A_AT	dickkopf homolog 3 (Xenopus laevis)
GGA.13762.1.S1_AT	collagen, type IV, alpha 6
GGA.1776.1.S1_AT	similar to nidogen 1; entactin; similar to nidogen; hypothetical protein LOC769017; nidogen 1
GGA.423.1.S1_AT	clusterin
GGA.3075.1.S1_AT	ADAMTS-like 3
GGA.4303.1.S1_AT	lysyl oxidase
GGA.3977.3.S1_A_AT	aggrecan
GGA.4723.1.S1_AT	leukocyte cell derived chemotaxin 1
GGAAFFX.25194.1.S1_AT	angiopoietin-like 5
GGA.345.1.S1_AT	sonic hedgehog homolog (Drosophila)
GGA.1907.1.S1_AT	platelet derived growth factor C
GGA.3357.2.S1_A_AT	lysozyme G-like 2
GGAAFFX.9557.1.S1_S_AT	laminin, alpha 3
GGA.9233.1.S1_AT	growth hormone receptor
GGA.4051.1.S1_AT	connective tissue growth factor
GGA.19.1.S1_AT	interleukin 1, beta
GGAAFFX.22988.1.S1_AT	collagen, type I, alpha 2
GGAAFFX.915.1.S1_AT	ADAM metallopeptidase with thrombospondin type 1 motif, 8
GGAAFFX.20103.1.S1_S_AT	growth differentiation factor 9
GGAAFFX.24555.1.S1_S_AT	collagen, type X, alpha 1(Schmid metaphyseal chondrodysplasia)
GGA.3572.1.S2_AT	CD44 molecule (Indian blood group)
GGAAFFX.200.1.S1_S_AT	fibrillin 2

GGA.2551.2.S1_S_AT	lactotransferrin
GGA.19892.1.S1_AT	WNT1 inducible signaling pathway protein 2

**Enrichment Score: 8.63155799029768**	**GOTERM_CC_FAT: 0005578~proteinaceous extracellular matrix**	**Count: 36**	***p* = 1.09E-10**
GGA.16844.2.S1_AT	collagen, type III, alpha 1		
GGAAFFX.2973.1.S1_AT	collagen, type IV, alpha 3		
GGA.8352.1.S1_AT	similar to merosin; similar to laminin alpha 2 chain; similar to laminin alpha 2; laminin, alpha 2 (merosin, congenital muscular dystrophy); similar to mKIAA4087 protein		
GGAAFFX.24388.1.S1_AT	microfibrillar associated protein 5		
GGAAFFX.20104.1.S1_S_AT	periostin, osteoblast specific factor		
GGA.822.1.S1_A_AT	elastin (supravalvular aortic stenosis, Williams-Beuren syndrome)		
GGA.481.1.S1_AT	fibromodulin		
GGA.866.1.S1_AT	collagen, type XVII, alpha 1		
GGA.1812.1.S1_AT	tumor necrosis factor receptor superfamily, member 11b (osteoprotegerin)		
GGA.155.1.S1_S_AT	lectin, galactoside-binding, soluble, 3		
GGAAFFX.7243.1.S1_AT	netrin 4		
GGAAFFX.9712.1.S1_AT	collagen, type VIII, alpha 1		
GGA.7471.2.A1_S_AT	heparan sulfate proteoglycan 2		
GGAAFFX.20952.1.A1_S_AT	collagen, type II, alpha 1		
GGA.4292.1.S1_AT	laminin, beta 2 (laminin S)		
GGA.16392.1.S1_AT	collagen, type XI, alpha 1		
GGA.9962.1.S1_AT	ectonucleoside triphosphate diphosphohydrolase 1		
GGA.2558.1.S1_A_AT	similar to collagen XIV; collagen, type XIV, alpha 1 (undulin); similar to collagen, type XIV, alpha 1 (undulin)		
GGAAFFX.26374.1.S1_AT	TIMP metallopeptidase inhibitor 4		
GGAAFFX.25090.2.S1_AT	collagen, type IV, alpha 2		
GGA.496.1.S1_AT	reelin		
GGA.13762.1.S1_AT	collagen, type IV, alpha 6		
GGA.1776.1.S1_AT	similar to nidogen 1; entactin; similar to nidogen; hypothetical protein LOC769017; nidogen 1		
GGA.3075.1.S1_AT	ADAMTS-like 3		
GGA.3928.1.S1_AT	hyaluronan and proteoglycan link protein 1		
GGA.3977.3.S1_A_AT	aggrecan		
GGAAFFX.9557.1.S1_S_AT	laminin, alpha 3		
GGA.17661.1.S1_S_AT	secreted protein, acidic, cysteine-rich (osteonectin)		
GGA.4051.1.S1_AT	connective tissue growth factor		
GGAAFFX.22984.1.S1_X_AT	collagen, type I, alpha 2		
GGAAFFX.915.1.S1_AT	ADAM metallopeptidase with thrombospondin type 1 motif, 8		
GGAAFFX.24555.1.S1_S_AT	collagen, type X, alpha 1(Schmid metaphyseal chondrodysplasia)		
GGA.648.1.S2_AT	fibroblast growth factor 1 (acidic)		
GGA.4990.1.S1_AT	collagen, type IX, alpha 1		
GGAAFFX.10781.1.S1_AT	collagen, type IV, alpha 1		
GGAAFFX.200.1.S1_S_AT	fibrillin 2		

**Enrichment Score: 5.047321550808001**	**GOTERM_BP_FAT: 0022610~biological adhesion**	**Count: 43**	***p* = 2.19E-07**
GGA.636.1.S1_AT	integrin-binding sialoprotein (bone sialoprotein, bone sialoprotein II)		
GGA.629.1.S1_AT	neuron-glia cell adhesion molecule (Ng-CAM)		
GGA.3448.1.S1_AT	thrombospondin 1		
GGA.2734.1.S1_AT	activated leukocyte cell adhesion molecule		
GGA.4738.1.S1_AT	neogenin		
GGA.10972.1.S1_AT	collagen, type XXII, alpha 1		
GGA.84.1.A1_AT	cadherin		
GGAAFFX.22178.3.S1_S_AT	Fc fragment of IgG binding protein		
GGAAFFX.12486.1.S1_S_AT	SCO-spondin homolog (Bos taurus)		
GGAAFFX.20104.1.S1_S_AT	periostin, osteoblast specific factor		
GGA.3551.1.S1_AT	secreted phosphoprotein 1 (osteopontin, bone sialoprotein I, early T-lymphocyte activation 1)		
GGAAFFX.25095.1.S1_AT	integrin, beta-like 1 (with EGF-like repeat domains)		
GGA.9505.1.A1_AT	protocadherin gamma subfamily C, 5		
GGA.728.1.S1_A_AT	cadherin 3, type 1, P-cadherin (placental)		
GGAAFFX.26173.2.S1_S_AT	FAT tumor suppressor homolog 2 (Drosophila)		
GGA.4821.1.S1_AT	collagen, type XVIII, alpha 1		
GGA.3903.1.S2_AT	cadherin 13, H-cadherin (heart)		
GGAAFFX.7996.1.S1_AT	collagen, type XV, alpha 1		
GGA.7895.1.S1_AT	EGF-like repeats and discoidin I-like domains 3		
GGAAFFX.1674.1.S1_AT	protocadherin 12		
GGA.607.1.S1_AT	bone morphogenetic protein receptor, type IB		
GGAAFFX.3588.2.S1_AT	cadherin, EGF LAG seven-pass G-type receptor 3 (flamingo homolog, Drosophila)		
GGA.4394.1.S1_AT	B-cell CLL/lymphoma 2		

GGAAFFX.9712.2.S1_S_AT	collagen, type VIII, alpha 1
GGAAFFX.23137.1.S1_S_AT	heparan sulfate proteoglycan 2
GGAAFFX.5250.1.S1_AT	similar to Hapln3-prov protein
GGAAFFX.20952.1.A1_S_AT	collagen, type II, alpha 1
GGA.3562.1.S1_AT	collagen, type XI, alpha 1
GGA.2558.1.S1_A_AT	similar to collagen XIV; collagen, type XIV, alpha 1 (undulin); similar to collagen, type XIV, alpha 1 (undulin)
GGA.496.1.S1_AT	reelin
GGA.1776.1.S1_AT	similar to nidogen 1; entactin; similar to nidogen; hypothetical protein LOC769017; nidogen 1
GGA.89.1.A1_X_AT	similar to cadherin-8; cadherin 8, type 2
GGA.3928.1.S1_AT	hyaluronan and proteoglycan link protein 1
GGA.3977.3.S1_A_AT	aggrecan
GGA.4051.1.S1_AT	connective tissue growth factor
GGA.10389.1.S1_AT	integrin, alpha 11
GGA.4990.1.S1_AT	collagen, type IX, alpha 1
GGAAFFX.6084.3.S1_S_AT	hypothetical protein LOC768874; carboxypeptidase X (M14 family), member 2
GGAAFFX.25535.1.S1_AT	protocadherin 1
GGA.890.1.S1_AT	SRY (sex determining region Y)-box 9
GGA.3572.1.S2_AT	CD44 molecule (Indian blood group)
GGA.1247.1.S1_AT	B6.1
GGA.75.1.A1_AT	thrombospondin 3

**Enrichment Score: 3.7909011438346285**	**GOTERM_BP_FAT: 0001501~skeletal system development**	**Count: 28**	***p* = 4.87E-08**
GGA.636.1.S1_AT	integrin-binding sialoprotein (bone sialoprotein, bone sialoprotein II)		
GGAAFFX.549.2.S1_AT	mitogen-activated protein kinase 14		
GGA.13388.1.S1_AT	dentin matrix acidic phosphoprotein		
GGAAFFX.3680.1.S1_AT	phosphoglycolate phosphatase		
GGAAFFX.9647.2.S1_AT	proprotein convertase subtilisin/kexin type 5		
GGA.3381.1.S2_AT	homeobox A3		
GGA.963.1.S1_AT	T-box 3		
GGA.3551.1.S1_AT	secreted phosphoprotein 1 (osteopontin, bone sialoprotein I, early T-lymphocyte activation 1)		
GGA.10508.1.S1_AT	distal-less homeobox 1		
GGA.155.1.S1_S_AT	lectin, galactoside-binding, soluble, 3		
GGA.473.1.S1_AT	Indian hedgehog homolog (Drosophila)		
GGA.926.1.S1_AT	homeobox B8		
GGA.607.1.S1_AT	bone morphogenetic protein receptor, type IB		
GGA.245.1.S1_AT	fibroblast growth factor 18		
GGA.7471.2.A1_S_AT	heparan sulfate proteoglycan 2		
GGAAFFX.20952.1.A1_S_AT	collagen, type II, alpha 1		
GGA.3562.1.S1_AT	collagen, type XI, alpha 1		
GGA.3652.1.S1_AT	C-type lectin domain family 3, member B		
GGA.28.1.S1_AT	nodal homolog (mouse)		
GGA.620.1.S1_AT	bone morphogenetic protein 5		
GGA.3977.3.S1_A_AT	aggrecan		
GGA.4723.1.S1_AT	leukocyte cell derived chemotaxin 1		
GGA.345.1.S1_AT	sonic hedgehog homolog (Drosophila)		
GGA.4051.1.S1_AT	connective tissue growth factor		
GGAAFFX.22984.1.S1_X_AT	collagen, type I, alpha 2		
GGA.4344.1.S1_AT	similar to SRF-related protein		
GGA.4990.1.S1_AT	collagen, type IX, alpha 1		
GGA.890.1.S1_AT	SRY (sex determining region Y)-box 9		

Top annotation clusters downregulated ≥3-fold from 3D fibrin TEC to mature fibrin TEC (2066 probe sets)

**Enrichment Score: 7.114049593474077**	**GOTERM_MF_FAT: 0000166~nucleotide binding**	**Count: 136**	***p* = 9.78E-08**
GGA.4350.1.S1_AT	FYN oncogene related to SRC, FGR, YES		
GGA.4350.1.S2_AT	FYN oncogene related to SRC, FGR, YES		
GGA.2738.1.S1_AT	X-ray repair complementing defective repair in Chinese hamster cells 6 (Ku autoantigen, 70kDa)		
GGAAFFX.11417.1.S1_AT	kinesin family member 11; hypothetical LOC426105; similar to similar to kinesin like protein		
GGAAFFX.4223.1.S1_S_AT	kinesin family member 11; hypothetical LOC426105; similar to similar to kinesin like protein		
GGAAFFX.8504.2.S1_S_AT	ATP-binding cassette, sub-family F (GCN20), member 2		
GGA.9834.2.S1_A_AT	thiamin pyrophosphokinase 1		
GGA.9834.1.S1_AT	thiamin pyrophosphokinase 1		
GGA.12290.1.S1_AT	X-ray repair complementing defective repair in Chinese hamster cells 2		
GGA.4522.2.S1_A_AT	DEAH (Asp-Glu-Ala-His) box polypeptide 15; hypothetical LOC425141; similar to putative RNA helicase and RNA dependent ATPase		
GGAAFFX.23924.2.S1_S_AT	kinesin family member 5C		

GGA.8075.1.S1_AT	kinesin family member 5C
GGAAFFX.6763.3.S1_S_AT	ribosomal protein S6 kinase, 90kDa, polypeptide 5
GGAAFFX.6763.4.S1_S_AT	ribosomal protein S6 kinase, 90kDa, polypeptide 5
GGAAFFX.6405.1.S1_AT	heat shock 70kDa protein 4-like
GGAAFFX.6405.2.S1_S_AT	heat shock 70kDa protein 4-like
GGA.10214.1.S1_S_AT	mitogen-activated protein kinase 12
GGA.7164.1.S1_AT	similar to cdc21p; similar to DNA replication initiator protein; minichromosome maintenance complex component 4
GGAAFFX.12208.1.S1_S_AT	kinesin family member 20A
GGA.11746.1.S1_AT	PDZ binding kinase
GGA.18387.1.S1_S_AT	aquarius homolog (mouse)
GGAAFFX.6168.1.S1_S_AT	aquarius homolog (mouse)
GGAAFFX.23051.1.S1_AT	aquarius homolog (mouse)
GGA.7224.2.S1_S_AT	nuclear receptor binding protein 1
GGA.9668.1.S1_S_AT	alpha thalassemia/mental retardation syndrome X-linked
GGAAFFX.2572.1.S1_AT	myosin IE
GGA.1147.1.S1_S_AT	mitogen-activated protein kinase 8
GGAAFFX.12967.1.S1_S_AT	heat shock protein 90kDa alpha (cytosolic), class A member 1
GGAAFFX.12402.1.S1_S_AT	structural maintenance of chromosomes 5; similar to structural maintenance of chromosomes 5
GGAAFFX.2058.1.S1_S_AT	conserved helix-loop-helix ubiquitous kinase
GGAAFFX.22528.1.S1_S_AT	STE20-like kinase (yeast)
GGA.2087.1.S1_S_AT	casein kinase 1, epsilon
GGA.3674.1.S1_AT	serine threonine kinase 39 (STE20/SPS1 homolog, yeast)
GGA.2372.1.S1_AT	RAD54 homolog B (S. cerevisiae)
GGAAFFX.23654.1.S1_AT	ubiquitin-activating enzyme E1-like 2
GGAAFFX.7476.1.S1_S_AT	ubiquitin-activating enzyme E1-like 2
GGA.5199.1.S1_AT	receptor (TNFRSF)-interacting serine-threonine kinase 1
GGAAFFX.4138.1.S1_S_AT	ATP-binding cassette, sub-family C (CFTR/MRP), member 1
GGA.17061.1.S1_S_AT	ATP-binding cassette, sub-family C (CFTR/MRP), member 1
GGA.274.1.S1_AT	platelet-derived growth factor receptor, alpha polypeptide
GGAAFFX.11402.1.S1_AT	NIMA (never in mitosis gene a)-related kinase 2
GGAAFFX.25392.2.S1_S_AT	6-phosphofructo-2-kinase/fructose-2,6-biphosphatase 4
GGAAFFX.10224.1.S1_AT	dopey family member 2
GGA.4637.1.S1_S_AT	tyrosyl-tRNA synthetase
GGA.6034.2.S1_A_AT	NIMA (never in mitosis gene a)-related kinase 6
GGA.6034.1.S1_AT	NIMA (never in mitosis gene a)-related kinase 6
GGAAFFX.8270.1.S1_AT	chaperonin containing TCP1, subunit 5 (epsilon)
GGAAFFX.22887.1.S1_S_AT	hypothetical LOC426502; Obg-like ATPase 1; similar to GTP-binding protein 9 (putative)
GGA.15973.1.S1_AT	receptor-interacting serine-threonine kinase 4
GGAAFFX.12701.1.S1_AT	polo-like kinase 1 (Drosophila)
GGA.15359.1.S1_S_AT	apoptotic peptidase activating factor 1
GGA.15359.1.S1_AT	apoptotic peptidase activating factor 1
GGA.2464.1.S2_AT	glutamate-ammonia ligase (glutamine synthetase)
GGA.3983.1.S1_AT	eukaryotic translation initiation factor 2-alpha kinase 1
GGA.5262.1.S1_S_AT	t-complex 1
GGAAFFX.11524.1.S1_S_AT	chaperonin containing TCP1, subunit 4 (delta)
GGA.4676.1.S1_S_AT	nucleotide binding protein 2 (MinD homolog, E. coli)
GGAAFFX.12693.1.S1_AT	RIO kinase 2 (yeast)
GGAAFFX.9739.1.S1_S_AT	RIO kinase 2 (yeast)
GGAAFFX.7711.1.S1_AT	kinesin family member 18A
GGAAFFX.11920.1.S1_S_AT	SWI/SNF related, matrix associated, actin dependent regulator of chromatin, subfamily a, member 5
GGA.2964.1.S1_S_AT	chaperonin containing TCP1, subunit 6A (zeta 1)
GGA.16540.1.S1_S_AT	helicase, lymphoid-specific
GGAAFFX.12267.1.S1_AT	ATPase, class II, type 9B
GGA.4555.1.S1_A_AT	heat shock 70kDa protein 8
GGA.5687.4.S1_A_AT	kinesin family member 2C
GGA.5687.2.S1_A_AT	kinesin family member 2C
GGA.6269.2.S1_S_AT	phenylalanyl-tRNA synthetase, beta subunit
GGAAFFX.12079.1.S1_AT	kinesin heavy chain member 2A
GGAAFFX.12000.1.S1_AT	succinate-CoA ligase, GDP-forming, beta subunit
GGAAFFX.20374.1.S1_AT	succinate-CoA ligase, GDP-forming, beta subunit
GGAAFFX.20374.1.S1_S_AT	succinate-CoA ligase, GDP-forming, beta subunit
GGAAFFX.22748.1.S1_AT	mitogen-activated protein kinase kinase kinase kinase 4; similar to mitogen-activated protein kinase kinase kinase kinase 4
GGAAFFX.25074.1.S1_S_AT	mitogen-activated protein kinase kinase kinase kinase 4; similar to mitogen-activated protein kinase kinase kinase kinase 4
GGAAFFX.5597.1.S1_AT	mitogen-activated protein kinase kinase kinase kinase 4; similar to mitogen-activated protein kinase kinase kinase kinase 4

GGA.17079.1.S1_S_AT	mitogen-activated protein kinase kinase kinase 7
GGAAFFX.24722.1.S1_S_AT	mitogen-activated protein kinase kinase kinase 7
GGAAFFX.11959.1.S1_S_AT	phosphoinositide-3-kinase, regulatory subunit 4, p150
GGAAFFX.7334.1.S1_S_AT	phosphoinositide-3-kinase, regulatory subunit 4, p150
GGA.9476.1.S1_AT	receptor tyrosine kinase-like orphan receptor 1
GGAAFFX.22977.1.S1_S_AT	similar to hensin
GGAAFFX.4800.1.S1_S_AT	myosin IB
GGAAFFX.22337.3.S1_S_AT	myosin IB
GGAAFFX.22337.2.S1_S_AT	myosin IB
GGA.2163.1.S1_AT	mitogen-activated protein kinase 1
GGA.2995.1.S1_AT	SFRS protein kinase 1
GGAAFFX.10518.1.S1_S_AT	maternal embryonic leucine zipper kinase
GGAAFFX.7284.1.S1_AT	dynein, cytoplasmic 1, light intermediate chain 1
GGAAFFX.11485.1.S1_AT	ATP-binding cassette, sub-family C (CFTR/MRP), member 4
GGAAFFX.9426.1.S1_AT	protein kinase, AMP-activated, alpha 1 catalytic subunit
GGAAFFX.20296.1.S1_AT	similar to similar to 60 kDa heat shock protein, mitochondrial precursor (Hsp60) (60 kDa chaperonin) (CPN60) (Heat shock protein 60) (HSP-60) (Mitochondrial matrix protein P1) (HSP-65); heat shock 60kDa protein 1 (chaperonin); similar to 60 kDa heat shock protein, mitochondrial precursor (Hsp60) (60 kDa chaperonin) (CPN60) (Heat shock protein 60) (HSP-60) (Mitochondrial matrix protein P1) (P60 lymphocyte protein) (HuCHA60)
GGA.9897.2.S1_AT	similar to similar to 60 kDa heat shock protein, mitochondrial precursor (Hsp60) (60 kDa chaperonin) (CPN60) (Heat shock protein 60) (HSP-60) (Mitochondrial matrix protein P1) (HSP-65); heat shock 60kDa protein 1 (chaperonin); similar to 60 kDa heat shock protein, mitochondrial precursor (Hsp60) (60 kDa chaperonin) (CPN60) (Heat shock protein 60) (HSP-60) (Mitochondrial matrix protein P1) (P60 lymphocyte protein) (HuCHA60)
GGAAFFX.11965.1.S1_S_AT	similar to similar to 60 kDa heat shock protein, mitochondrial precursor (Hsp60) (60 kDa chaperonin) (CPN60) (Heat shock protein 60) (HSP-60) (Mitochondrial matrix protein P1) (HSP-65); heat shock 60kDa protein 1 (chaperonin); similar to 60 kDa heat shock protein, mitochondrial precursor (Hsp60) (60 kDa chaperonin) (CPN60) (Heat shock protein 60) (HSP-60) (Mitochondrial matrix protein P1) (P60 lymphocyte protein) (HuCHA60)
GGAAFFX.20296.1.S1_S_AT	similar to similar to 60 kDa heat shock protein, mitochondrial precursor (Hsp60) (60 kDa chaperonin) (CPN60) (Heat shock protein 60) (HSP-60) (Mitochondrial matrix protein P1) (HSP-65); heat shock 60kDa protein 1 (chaperonin); similar to 60 kDa heat shock protein, mitochondrial precursor (Hsp60) (60 kDa chaperonin) (CPN60) (Heat shock protein 60) (HSP-60) (Mitochondrial matrix protein P1) (P60 lymphocyte protein) (HuCHA60)
GGAAFFX.5725.1.S1_AT	actin-like 6A; hypothetical protein LOC772110
GGA.8210.1.S1_S_AT	vacuolar protein sorting 4 homolog B (S. cerevisiae)
GGAAFFX.21211.1.S1_S_AT	vacuolar protein sorting 4 homolog B (S. cerevisiae)
GGAAFFX.25174.1.S1_S_AT	spastic ataxia of Charlevoix-Saguenay (sacsin)
GGAAFFX.4938.1.S1_AT	phosphatidylinositol-5-phosphate 4-kinase, type II, alpha
GGAAFFX.1523.1.S1_S_AT	casein kinase 1, gamma 1; similar to KIAA0101
GGA.5962.1.S1_AT	actin, beta-like 2; actin, alpha, cardiac muscle 1; actin, alpha 1, skeletal muscle
GGA.3200.1.S1_S_AT	structural maintenance of chromosomes 4
GGAAFFX.10149.2.S1_S_AT	DEAD (Asp-Glu-Ala-Asp) box polypeptide 43
GGAAFFX.10149.1.S1_AT	DEAD (Asp-Glu-Ala-Asp) box polypeptide 43
GGA.805.1.S1_AT	EPH receptor A3
GGAAFFX.24294.1.S1_AT	AFG3 ATPase family gene 3-like 2 (yeast)
GGA.764.1.S1_AT	activin A receptor, type IIB
GGAAFFX.8426.1.S1_S_AT	pyruvate dehydrogenase kinase, isozyme 1; hypothetical LOC425178
GGAAFFX.11753.1.S1_AT	pyruvate dehydrogenase kinase, isozyme 1; hypothetical LOC425178
GGA.7719.1.S1_S_AT	chromodomain helicase DNA binding protein 7
GGAAFFX.24083.1.S1_S_AT	WNK lysine deficient protein kinase 1
GGA.4066.1.S1_AT	structural maintenance of chromosomes 2
GGA.19118.1.S1_AT	ATP-binding cassette, sub-family D (ALD), member 2
GGAAFFX.13032.1.S1_S_AT	kinesin family member 23
GGAAFFX.6712.1.S1_AT	protein kinase D3
GGA.18271.1.S1_AT	helicase, POLQ-like
GGA.4076.1.S1_AT	ARP3 actin-related protein 3 homolog (yeast)
GGA.8984.1.S1_AT	ARP3 actin-related protein 3 homolog (yeast)
GGA.11492.1.S1_AT	similar to Plaucible mixed-lineage kinase protein; similar to MLTK-alpha; similar to MLTK alpha; similar to mixed lineage kinase ZAK; sterile alpha motif and leucine zipper containing kinase AZK
GGA.1138.1.S1_S_AT	alanyl-tRNA synthetase
GGA.7689.2.S1_X_AT	calcium/calmodulin-dependent serine protein kinase (MAGUK family)
GGA.421.2.S1_A_AT	thymidine kinase 1, soluble
GGA.13248.1.S1_AT	ARP2 actin-related protein 2 homolog (yeast)
GGA.3020.1.S1_AT	ARP2 actin-related protein 2 homolog (yeast)
GGAAFFX.23603.1.S1_S_AT	kinesin family member 26A
GGA.726.2.S1_A_AT	cell division cycle 2, G1 to S and G2 to M

GGA.5656.1.S1_A_AT	DEAD (Asp-Glu-Ala-Asp) box polypeptide 50		
GGA.16459.1.S1_S_AT	DEAD (Asp-Glu-Ala-Asp) box polypeptide 50		
GGAAFFX.12510.1.S1_AT	hypothetical protein LOC776374; dihydroxyacetone kinase 2 homolog (S. cerevisiae)		
GGAAFFX.3712.1.S1_S_AT	hypothetical protein LOC776374; dihydroxyacetone kinase 2 homolog (S. cerevisiae)		
GGAAFFX.5788.1.S1_S_AT	CDC42 binding protein kinase alpha (DMPK-like)		
GGA.3729.1.S1_AT	calcium/calmodulin-dependent protein kinase kinase 2, beta		
GGA.3411.2.S1_AT	mitogen-activated protein kinase 6; similar to Mapk6 protein		
GGAAFFX.20558.1.S1_AT	chromodomain helicase DNA binding protein 1		
GGA.8463.1.S1_S_AT	chromodomain helicase DNA binding protein 1		
GGA.3500.1.S1_AT	Rho-associated, coiled-coil containing protein kinase 2		
GGAAFFX.10528.1.S1_AT	Rho-associated, coiled-coil containing protein kinase 2		
GGA.19162.1.S1_AT	ret proto-oncogene		
GGAAFFX.20123.1.S1_S_AT	chaperonin containing TCP1, subunit 8 (theta)		
GGAAFFX.11896.1.S1_S_AT	PRP4 pre-mRNA processing factor 4 homolog B (yeast)		
GGA.8038.2.S1_S_AT	replication factor C (activator 1) 4, 37kDa		
GGAAFFX.12380.1.S1_S_AT	protein kinase, AMP-activated, gamma 2 non-catalytic subunit		
GGAAFFX.10553.2.S1_S_AT	ATPase family, AAA domain containing 2B		
GGA.3058.1.S1_AT	succinocarboxamide synthetase		
GGAAFFX.6121.1.S1_S_AT	asparagine synthetase		
GGAAFFX.10856.1.S1_AT	hypothetical LOC428066		
GGAAFFX.845.1.S1_S_AT	mitogen-activated protein kinase kinase 2		
GGA.2151.1.S1_AT	ATPase, Ca++ transporting, cardiac muscle, slow twitch 2		
GGA.12424.1.S1_AT	hypothetical protein LOC776448; hypothetical protein LOC771499; kinesin family member 21A; similar to kinesin family member 21A		
GGAAFFX.10124.2.S1_S_AT	receptor-interacting serine-threonine kinase 2		
GGA.2010.2.S1_A_AT	topoisomerase (DNA) II alpha 170kDa		
GGA.2517.2.S1_A_AT	CHK2 checkpoint homolog (S. pombe)		
GGA.7180.1.S1_S_AT	BUB1 budding uninhibited by benzimidazoles 1 homolog (yeast)		
GGA.8770.2.S1_A_AT	chromosome 9 open reading frame 103		
GGA.633.1.S1_AT	EPH receptor B6		
GGA.4109.1.S2_AT	ATP-binding cassette, sub-family B (MDR/TAP), member 1		
GGA.4109.1.S1_AT	ATP-binding cassette, sub-family B (MDR/TAP), member 1		
GGAAFFX.11571.1.S1_AT	vaccinia related kinase 1		
GGA.11797.1.S1_S_AT	vaccinia related kinase 1		
GGAAFFX.24078.1.S1_S_AT	similar to Trio splicing; triple functional domain (PTPRF interacting)		
GGAAFFX.644.2.S1_S_AT	mitogen-activated protein kinase kinase 4		
GGAAFFX.2325.1.S1_AT	myosin IXB		
GGAAFFX.2326.1.S1_AT	myosin IXB		
GGAAFFX.20762.1.S1_AT	ch-runtB2		
GGA.7759.1.S1_A_AT	chaperonin containing TCP1, subunit 2 (beta)		
GGA.4871.1.S1_AT	proteasome (prosome, macropain) 26S subunit, ATPase, 5		
GGAAFFX.22965.1.S1_S_AT	tousled-like kinase 1		
GGAAFFX.10484.1.S1_AT	ATPase family, AAA domain containing 2		
GGAAFFX.10107.1.S1_AT	TTK protein kinase		
GGA.6326.1.S1_S_AT	similar to Gu protein		
GGA.1331.2.S1_A_AT	topoisomerase (DNA) II beta 180kDa		
GGAAFFX.24044.3.S1_S_AT	tyrosyl-tRNA synthetase 2, mitochondrial		
GGA.4358.1.S1_AT	kinesin family member 4A		
GGA.4358.2.S1_A_AT	kinesin family member 4A		
**Enrichment Score: 6.1517929297878915**	**GOTERM_CC_FAT: 0005694~chromosome**	**Count: 43**	***p* = 8.46E-09**
GGA.2738.1.S1_AT	X-ray repair complementing defective repair in Chinese hamster cells 6 (Ku autoantigen, 70kDa)		
GGAAFFX.12938.12.S1_S_AT	RAD18 homolog (S. cerevisiae)		
GGA.4252.1.S2_AT	ZW10, kinetochore associated, homolog (Drosophila)		
GGA.11314.1.S1_AT	integrin beta 3 binding protein (beta3-endonexin)		
GGAAFFX.20558.1.S1_AT	chromodomain helicase DNA binding protein 1		
GGA.8463.1.S1_S_AT	chromodomain helicase DNA binding protein 1		
GGA.8462.1.S1_AT	BUB1 budding uninhibited by benzimidazoles 1 homolog beta (yeast)		
GGAAFFX.13010.1.S1_AT	NUF2, NDC80 kinetochore complex component, homolog (S. cerevisiae)		
GGA.9459.1.S1_S_AT	NUF2, NDC80 kinetochore complex component, homolog (S. cerevisiae)		
GGAAFFX.12584.1.S1_AT	transcriptional adaptor 2 (ADA2 homolog, yeast)-like		
GGA.5262.1.S1_S_AT	t-complex 1		
GGAAFFX.11896.1.S1_S_AT	PRP4 pre-mRNA processing factor 4 homolog B (yeast)		
GGAAFFX.10866.1.S1_S_AT	nuclear fragile X mental retardation protein interacting protein 1		
GGA.8038.2.S1_S_AT	replication factor C (activator 1) 4, 37kDa		
GGA.14210.2.S1_AT	MLF1 interacting protein		
GGA.3200.1.S1_S_AT	structural maintenance of chromosomes 4		
GGA.31.1.S1_AT	centromere protein I		

GGA.944.1.S1_AT	centromere protein H
GGA.4287.1.S1_AT	protein phosphatase 2 (formerly 2A), catalytic subunit, beta isoform
GGA.2010.2.S1_A_AT	topoisomerase (DNA) II alpha 170kDa
GGAAFFX.12571.1.S1_S_AT	origin recognition complex, subunit 3-like (yeast)
GGA.7180.1.S1_S_AT	BUB1 budding uninhibited by benzimidazoles 1 homolog (yeast)
GGA.4349.1.S1_S_AT	SWI/SNF related, matrix associated, actin dependent regulator of chromatin, subfamily b, member 1
GGA.7431.1.S1_AT	origin recognition complex, subunit 4-like (yeast)
GGA.16540.1.S1_S_AT	helicase, lymphoid-specific
GGA.11620.1.S1_AT	similar to single-strand telomeric DNA-binding protein; cPot1; POT1 protection of telomeres 1 homolog (S. pombe)
GGA.7719.1.S1_S_AT	chromodomain helicase DNA binding protein 7
GGA.1251.2.S1_A_AT	inner centromere protein antigens 135/155kDa
GGAAFFX.6156.1.S1_AT	NSL1, MIND kinetochore complex component, homolog (S. cerevisiae)
GGA.4066.1.S1_AT	structural maintenance of chromosomes 2
GGA.4493.1.S2_AT	breast cancer 1, early onset
GGAAFFX.12402.1.S1_S_AT	structural maintenance of chromosomes 5; similar to structural maintenance of chromosomes 5
GGA.196.1.S1_AT	nibrin
GGA.3705.1.S1_AT	centromere protein F, 350/400ka (mitosin)
GGAAFFX.24391.1.S1_AT	PDS5, regulator of cohesion maintenance, homolog A (S. cerevisiae)
GGAAFFX.12023.1.S1_AT	transcription factor 3 (E2A immunoglobulin enhancer binding factors E12/E47)
GGA.5576.1.S1_A_AT	similar to germinal histone H4 gene; similar to histone H4; germinal histone H4 gene
GGA.5576.2.S1_AT	similar to germinal histone H4 gene; similar to histone H4; germinal histone H4 gene
GGA.7812.2.S1_A_AT	origin recognition complex, subunit 6 like (yeast)
GGAAFFX.8595.2.S1_S_AT	centromere protein N; similar to centromere protein N
GGA.1551.2.S1_A_AT	baculoviral IAP repeat-containing 5 (survivin)
GGA.7355.1.S1_AT	MIS12, MIND kinetochore complex component, homolog (yeast)
GGAAFFX.20752.1.S1_AT	SET domain containing (lysine methyltransferase) 7
GGAAFFX.23665.1.S1_X_AT	similar to histone H2B
GGAAFFX.23665.1.S1_AT	similar to histone H2B
GGA.1331.2.S1_A_AT	topoisomerase (DNA) II beta 180kDa
GGAAFFX.11402.1.S1_AT	NIMA (never in mitosis gene a)-related kinase 2

**Enrichment Score: 4.492052522**	**GOTERM_CC_FAT: 0005856~cytoskeleton**	**Count: 65**	***p* = 3.13E-04**
GGAAFFX.11417.1.S1_AT	kinesin family member 11; hypothetical LOC426105; similar to similar to kinesin like protein		
GGAAFFX.4223.1.S1_S_AT	kinesin family member 11; hypothetical LOC426105; similar to similar to kinesin like protein		
GGAAFFX.7284.1.S1_AT	dynein, cytoplasmic 1, light intermediate chain 1		
GGAAFFX.5404.1.S1_S_AT	nucleolar and spindle associated protein 1		
GGA.4108.1.S2_AT	tropomyosin 1 (alpha)		
GGA.4108.4.S1_A_AT	tropomyosin 1 (alpha)		
GGAAFFX.11794.1.S1_S_AT	capping protein (actin filament) muscle Z-line, alpha 1		
GGA.2783.1.S1_AT	capping protein (actin filament) muscle Z-line, alpha 1		
GGAAFFX.23924.2.S1_S_AT	kinesin family member 5C		
GGA.8075.1.S1_AT	kinesin family member 5C		
GGA.9459.1.S1_S_AT	NUF2, NDC80 kinetochore complex component, homolog (S. cerevisiae)		
GGAAFFX.13010.1.S1_AT	NUF2, NDC80 kinetochore complex component, homolog (S. cerevisiae)		
GGAAFFX.12208.1.S1_S_AT	kinesin family member 20A		
GGA.15766.1.S1_AT	eukaryotic translation initiation factor 6		
GGA.5962.1.S1_AT	actin, beta-like 2; actin, alpha, cardiac muscle 1; actin, alpha 1, skeletal muscle		
GGA.4477.1.S1_S_AT	chromosome 21 open reading frame 33; hypothetical protein LOC777096; similar to homolog of zebrafish ES1		
GGA.3957.2.S1_A_AT	enabled homolog (Drosophila)		
GGA.1899.1.S1_A_AT	nestin		
GGAAFFX.2572.1.S1_AT	myosin IE		
GGA.9818.1.S1_S_AT	developmentally regulated GTP binding protein 1		
GGA.185.1.S1_A_AT	actin filament associated protein 1		
GGAAFFX.13032.1.S1_S_AT	kinesin family member 23		
GGA.2375.1.S1_AT	catenin (cadherin-associated protein), alpha 2		
GGAAFFX.12568.1.S1_AT	coiled-coil domain containing 5 (spindle associated)		
GGAAFFX.11652.1.S1_AT	twinfilin, actin-binding protein, homolog 2 (Drosophila); WD repeat domain 82		
GGA.4076.1.S1_AT	ARP3 actin-related protein 3 homolog (yeast)		
GGA.8984.1.S1_AT	ARP3 actin-related protein 3 homolog (yeast)		
GGA.13558.1.S1_AT	ADP-ribosylation factor-like 8B; ADP-ribosylation factor-like 8A		
GGA.14450.1.S1_AT	ADP-ribosylation factor-like 8B; ADP-ribosylation factor-like 8A		
GGA.13442.1.S1_AT	radixin		
GGA.13442.1.S2_S_AT	radixin		
GGAAFFX.6144.1.S1_S_AT	protein tyrosine phosphatase, non-receptor type 14		
GGA.1551.2.S1_A_AT	baculoviral IAP repeat-containing 5 (survivin)		

GGA.3020.1.S1_AT	ARP2 actin-related protein 2 homolog (yeast)
GGA.13248.1.S1_AT	ARP2 actin-related protein 2 homolog (yeast)
GGA.3146.1.S1_AT	cyclin B2
GGA.4661.2.S1_AT	tubulin, alpha 1c
GGAAFFX.11402.1.S1_AT	NIMA (never in mitosis gene a)-related kinase 2
GGA.2409.1.S2_AT	WD repeat domain 1
GGA.16968.1.S1_S_AT	HAUS augmin-like complex, subunit 6; hypothetical LOC427239
GGAAFFX.12114.1.S1_AT	HAUS augmin-like complex, subunit 6; hypothetical LOC427239
GGA.3179.1.S1_AT	brain abundant, membrane attached signal protein 1
GGAAFFX.12587.1.S1_S_AT	cytoskeleton associated protein 2
GGA.714.1.S1_AT	formin 1
GGA.714.1.S2_AT	formin 1
GGA.3500.1.S1_AT	Rho-associated, coiled-coil containing protein kinase 2
GGAAFFX.10528.1.S1_AT	Rho-associated, coiled-coil containing protein kinase 2
GGA.3041.1.S1_AT	troponin C type 1 (slow)
GGA.5262.1.S1_S_AT	t-complex 1
GGA.7794.1.S1_AT	tubulin, beta 6; similar to Tubulin beta-5 chain (Beta-tubulin class-V); similar to tubulin, beta 3; tubulin, beta 2B
GGA.1815.1.S1_AT	tubulin, beta 6; similar to Tubulin beta-5 chain (Beta-tubulin class-V); similar to tubulin, beta 3; tubulin, beta 2B
GGA.6254.1.S1_S_AT	actin related protein 2/3 complex, subunit 5, 16kDa
GGA.1368.1.S2_AT	lamin B1
GGA.1368.1.S1_AT	lamin B1
GGA.11453.1.S1_AT	similar to tropomodulin 3 (ubiquitous); tropomodulin 3 (ubiquitous)
GGAAFFX.11865.1.S1_S_AT	ARP6 actin-related protein 6 homolog (yeast)
GGA.715.1.S1_AT	lamin A
GGA.3130.2.S1_A_AT	CAP-GLY domain containing linker protein 1
GGA.4287.1.S1_AT	protein phosphatase 2 (formerly 2A), catalytic subunit, beta isoform
GGAAFFX.21017.1.S1_S_AT	caldesmon 1
GGA.8000.1.S1_AT	FERM, RhoGEF (ARHGEF) and pleckstrin domain protein 1 (chondrocyte-derived)
GGAAFFX.24513.3.S1_S_AT	erythrocyte membrane protein band 4.1-like 3; similar to Band 4.1-like protein 3 (4.1B) (Differentially expressed in adenocarcinoma of the lung protein 1) (DAL-1)
GGAAFFX.24513.10.S1_S_AT	erythrocyte membrane protein band 4.1-like 3; similar to Band 4.1-like protein 3 (4.1B) (Differentially expressed in adenocarcinoma of the lung protein 1) (DAL-1)
GGAAFFX.24513.7.S1_S_AT	erythrocyte membrane protein band 4.1-like 3; similar to Band 4.1-like protein 3 (4.1B) (Differentially expressed in adenocarcinoma of the lung protein 1) (DAL-1)
GGA.787.2.S1_A_AT	cofilin 2 (muscle)
GGA.388.2.S1_A_AT	similar to beta-keratin related protein; beta-keratin related protein
GGA.388.1.S1_AT	similar to beta-keratin related protein; beta-keratin related protein
GGA.1251.2.S1_A_AT	inner centromere protein antigens 135/155kDa
GGA.3635.1.S1_AT	cortactin
GGA.3635.2.S1_S_AT	cortactin
GGAAFFX.12079.1.S1_AT	kinesin heavy chain member 2A
GGA.3705.1.S1_AT	centromere protein F, 350/400ka (mitosin)
GGAAFFX.2326.1.S1_AT	myosin IXB
GGAAFFX.2325.1.S1_AT	myosin IXB
GGA.698.1.S2_AT	vinculin
GGA.15343.1.S1_AT	similar to WAS/WASL interacting protein family, member 1; WAS/WASL interacting protein family, member 1
GGAAFFX.4800.1.S1_S_AT	myosin IB
GGAAFFX.22337.3.S1_S_AT	myosin IB
GGAAFFX.22337.2.S1_S_AT	myosin IB
GGAAFFX.21767.1.S1_S_AT	stathmin 1
GGA.2844.2.S1_S_AT	stathmin 1
GGAAFFX.12738.1.S1_S_AT	cell division cycle 16 homolog (S. cerevisiae)
GGA.7271.1.S1_S_AT	membrane protein, palmitoylated 1, 55kDa
GGAAFFX.12737.1.S1_AT	membrane protein, palmitoylated 1, 55kDa
GGA.4895.1.S2_AT	destrin (actin depolymerizing factor)
GGA.2163.1.S1_AT	mitogen-activated protein kinase 1
GGA.4358.1.S1_AT	kinesin family member 4A
GGA.4358.2.S1_A_AT	kinesin family member 4A
GGA.18411.1.S1_AT	microtubule-associated protein 1B
GGA.2129.1.S1_S_AT	microtubule-associated protein 1B

Top annotation clusters downregulated ≥2<3-fold from 3D fibrin TEC to mature fibrin TEC (3213 probe sets)

**Enrichment Score: 6.124052997734279**	**GOTERM_CC_FAT: 0043233~organelle lumen**	**Count: 78**	***p* = 1.25E-08**
GGAAFFX.5746.1.S1_AT	partner of NOB1 homolog (S. cerevisiae)		

GGAAFFX.8064.1.S1_S_AT	PAK1 interacting protein 1
GGA.1297.1.S1_AT	lamin B2
GGAAFFX.11791.1.S1_S_AT	mitochondrial ribosomal protein L3
GGA.9897.1.S1_AT	similar to similar to 60 kDa heat shock protein, mitochondrial precursor (Hsp60) (60 kDa chaperonin) (CPN60) (Heat shock protein 60) (HSP-60) (Mitochondrial matrix protein P1) (HSP-65); heat shock 60kDa protein 1 (chaperonin); similar to 60 kDa heat shock protein, mitochondrial precursor (Hsp60) (60 kDa chaperonin) (CPN60) (Heat shock protein 60) (HSP-60) (Mitochondrial matrix protein P1) (P60 lymphocyte protein) (HuCHA60)
GGA.1329.1.S2_AT	aryl hydrocarbon receptor nuclear translocator-like
GGA.9585.1.S1_A_AT	processing of precursor 4, ribonuclease P/MRP subunit (S. cerevisiae)
GGA.9585.1.S1_S_AT	processing of precursor 4, ribonuclease P/MRP subunit (S. cerevisiae)
GGA.18010.1.S1_AT	trimethyllysine hydroxylase, epsilon
GGA.1029.3.S1_AT	retinoblastoma binding protein 4
GGA.1029.1.S1_A_AT	retinoblastoma binding protein 4
GGA.6220.2.S1_A_AT	ICER protein
GGA.891.1.S1_AT	ferredoxin 1
GGA.16450.1.S1_AT	glutamate dehydrogenase 1
GGAAFFX.4414.1.S1_S_AT	large subunit GTPase 1 homolog (S. cerevisiae)
GGA.4434.1.S1_AT	TBP-like 1
GGAAFFX.12860.1.S1_AT	thioredoxin domain containing 5 (endoplasmic reticulum)
GGA.3213.1.S1_AT	E2F transcription factor 1
GGA.5355.1.S1_AT	general transcription factor IIA, 2, 12kDa
GGA.4446.1.S3_AT	G protein-coupled receptor kinase interactor 2
GGA.4446.1.S2_AT	G protein-coupled receptor kinase interactor 2
GGA.4446.1.S1_AT	G protein-coupled receptor kinase interactor 2
GGAAFFX.11945.1.S1_S_AT	eukaryotic translation initiation factor 6
GGAAFFX.12036.1.S1_S_AT	three prime histone mRNA exonuclease 1
GGA.2061.1.S1_AT	GATA binding protein 6
GGA.12337.3.S1_S_AT	Werner syndrome
GGAAFFX.13131.1.S1_S_AT	GrpE-like 1, mitochondrial (E. coli)
GGA.4512.3.S1_AT	ribosomal protein S14
GGAAFFX.11830.1.S1_S_AT	hypoxia up-regulated 1
GGA.5235.1.S1_AT	hypoxia up-regulated 1
GGAAFFX.12116.1.S1_AT	Sjogren syndrome antigen B (autoantigen La)
GGA.1146.1.S1_S_AT	Sjogren syndrome antigen B (autoantigen La)
GGA.12072.2.S1_A_AT	mediator of RNA polymerase II transcription, subunit 31 homolog (S. cerevisiae)
GGA.4597.1.S1_AT	TAF13 RNA polymerase II, TATA box binding protein (TBP)-associated factor, 18kDa
GGAAFFX.11969.1.S1_AT	tripartite motif-containing 8
GGA.3803.1.S1_AT	E1A binding protein p400
GGAAFFX.12180.1.S1_S_AT	(xeroderma pigmentosum group B complementing)
GGAAFFX.12338.1.S1_S_AT	tuftelin interacting protein 11
GGAAFFX.11958.1.S1_S_AT	nucleolar protein 11
GGAAFFX.12876.1.S1_S_AT	ash2 (absent, small, or homeotic)-like (Drosophila)
GGAAFFX.1795.3.S1_S_AT	transcription factor Dp-2 (E2F dimerization partner 2)
GGAAFFX.1795.1.S1_AT	transcription factor Dp-2 (E2F dimerization partner 2)
GGAAFFX.11797.1.S1_AT	SWI/SNF related, matrix associated, actin dependent regulator of chromatin, subfamily e, member 1
GGA.4954.3.S1_AT	TNFAIP3 interacting protein 1
GGA.8825.1.S1_AT	macrophage erythroblast attacher
GGA.6364.3.S1_AT	RNA binding motif protein 19
GGAAFFX.12861.1.S1_S_AT	apoptosis antagonizing transcription factor
GGA.4133.1.S1_S_AT	similar to SMAD, mothers against DPP homolog 5 (Drosophila); SMAD family member 5
GGA.9488.1.S1_AT	aprataxin
GGA.2110.1.S2_AT	histone deacetylase 3
GGA.2110.1.S1_AT	histone deacetylase 3
GGA.3952.1.S1_AT	transcription factor Dp-1
GGAAFFX.11733.1.S1_S_AT	NOL1/NOP2/Sun domain family, member 2
GGA.5517.1.S1_S_AT	similar to nuclear DNA-binding protein; small unique nuclear receptor corepressor; C1D DNA-binding protein
GGAAFFX.13164.1.S1_AT	similar to nuclear DNA-binding protein; small unique nuclear receptor corepressor; C1D DNA-binding protein
GGAAFFX.12255.1.S1_AT	similar to polyhomeotic homolog 1 (Drosophila); polyhomeotic homolog 1 (Drosophila)
GGA.4036.1.S1_AT	proliferating cell nuclear antigen
GGA.4811.3.S1_S_AT	heterogeneous nuclear ribonucleoprotein K
GGAAFFX.11221.1.S1_S_AT	WD repeats and SOF1 domain containing; similar to WD repeats and SOF1 domain containing
GGAAFFX.11836.1.S1_S_AT	WD repeats and SOF1 domain containing; similar to WD repeats and SOF1 domain containing
GGAAFFX.11836.1.S1_AT	WD repeats and SOF1 domain containing; similar to WD repeats and SOF1 domain containing
GGAAFFX.6371.2.S1_S_AT	integrator complex subunit 10
GGAAFFX.20726.1.S1_S_AT	endoplasmic reticulum protein 29

GGAAFFX.12838.1.S1_S_AT	endoplasmic reticulum protein 29
GGAAFFX.13179.1.S1_S_AT	transcription elongation factor A (SII), 1
GGAAFFX.2596.2.S1_S_AT	suppressor of var1, 3-like 1 (S. cerevisiae)
GGAAFFX.12457.1.S1_S_AT	suppressor of var1, 3-like 1 (S. cerevisiae)
GGA.1006.2.S1_A_AT	TATA box binding protein
GGAAFFX.12161.1.S1_S_AT	transient receptor potential cation channel, subfamily C, member 4 associated protein
GGA.2694.1.S2_S_AT	lymphoid enhancer-binding factor 1; similar to lymphoid enhancer-binding factor 1
GGAAFFX.8604.1.S1_AT	UTP15, U3 small nucleolar ribonucleoprotein, homolog (S. cerevisiae)
GGAAFFX.12917.1.S1_S_AT	cleavage and polyadenylation specific factor 3-like
GGA.4061.2.S1_A_AT	high-mobility group box 2
GGAAFFX.21604.1.S1_S_AT	mediator complex subunit 22; similar to Surf5b
GGAAFFX.21943.1.S1_S_AT	mediator complex subunit 15
GGA.1355.1.S1_AT	complement component 1, q subcomponent binding protein
GGAAFFX.13054.1.S1_AT	kelch-like 7 (Drosophila)
GGA.3120.1.S1_AT	kelch-like 7 (Drosophila)
GGA.2835.1.S1_A_AT	WW domain binding protein 4 (formin binding protein 21)
GGAAFFX.12843.1.S1_AT	5'-3' exoribonuclease 2
GGAAFFX.6135.1.S1_AT	centromere protein F, 350/400ka (mitosin)
GGAAFFX.20508.1.S1_S_AT	centromere protein F, 350/400ka (mitosin)
GGA.7023.1.S1_AT	mediator complex subunit 1
GGAAFFX.24919.1.S1_AT	mediator complex subunit 1
GGA.1356.1.S1_AT	RAD52 motif 1
GGA.9141.1.S1_S_AT	neurofibromin 2 (bilateral acoustic neuroma)
GGA.4979.1.S1_AT	transcription factor 3 (E2A immunoglobulin enhancer binding factors E12/E47)
GGAAFFX.20792.1.S1_S_AT	zinc finger E-box binding homeobox 1
GGA.1250.1.S1_AT	protein phosphatase 1, catalytic subunit, beta isoform
GGAAFFX.12113.1.S1_AT	metastasis associated 1
GGA.7478.1.S1_S_AT	metastasis associated 1
GGA.18929.1.S1_S_AT	similar to WAS/WASL interacting protein family, member 1; WAS/WASL interacting protein family, member 1
GGA.1149.1.S1_AT	similar to WAS/WASL interacting protein family, member 1; WAS/WASL interacting protein family, member 1
GGA.1447.3.S1_S_AT	integrator complex subunit 2
GGAAFFX.12093.1.S1_S_AT	integrator complex subunit 2
GGAAFFX.5416.2.S1_S_AT	Rtf1, Paf1/RNA polymerase II complex component, homolog (S. cerevisiae)
GGA.2707.2.S1_A_AT	clock homolog (mouse)

**Enrichment Score: 5.5949115557968465**	**GOTERM_BP_FAT: 0043632~modification-dependent macromolecule catabolic process**	**Count: 42**	***p* = 8.69E-08**
GGAAFFX.21410.1.S1_S_AT	similar to ubiquitin protein ligase E3B; similar to ubiquitin protein ligase; ubiquitin protein ligase E3B		
GGA.17096.1.S1_S_AT	membrane-associated ring finger (C3HC4) 5		
GGAAFFX.11480.1.S1_S_AT	ubiquitin-fold modifier 1		
GGAAFFX.12503.1.S1_S_AT	similar to Usp10-prov protein; ubiquitin specific peptidase 10; hypothetical LOC430975		
GGA.3193.3.S1_AT	similar to Usp10-prov protein; ubiquitin specific peptidase 10; hypothetical LOC430975		
GGA.9399.1.S1_AT	YOD1 OTU deubiquinating enzyme 1 homolog (S. cerevisiae)		
GGAAFFX.13122.1.S1_S_AT	proteasome (prosome, macropain) subunit, alpha type, 2		
GGAAFFX.13093.1.S1_S_AT	Cas-Br-M (murine) ecotropic retroviral transforming sequence-like 1		
GGAAFFX.12230.1.S1_AT	Cas-Br-M (murine) ecotropic retroviral transforming sequence; ubiquitination factor E4A (UFD2 homolog, yeast)		
GGA.4159.2.S1_A_AT	TMEM189-UBE2V1 readthrough transcript		
GGA.13191.1.S1_S_AT	chromosome 10 open reading frame 46		
GGA.1295.1.S1_AT	ubiquitin-conjugating enzyme E2A (RAD6 homolog)		
GGAAFFX.12597.1.S1_AT	ubiquitin specific peptidase 1		
GGAAFFX.25326.1.S1_AT	ariadne homolog, ubiquitin-conjugating enzyme E2 binding protein, 1 (Drosophila)		
GGAAFFX.25219.1.S1_AT	ariadne homolog, ubiquitin-conjugating enzyme E2 binding protein, 1 (Drosophila)		
GGA.8240.1.S1_AT	arginyltransferase 1		
GGAAFFX.12236.1.S1_S_AT	ubiquitin carboxyl-terminal hydrolase L5		
GGAAFFX.12888.1.S1_S_AT	mindbomb homolog 2 (Drosophila)		
GGAAFFX.2100.1.S1_AT	ubiquitin specific peptidase 42		
GGA.3661.1.S1_A_AT	proteasome (prosome, macropain) subunit, alpha type, 1		
GGAAFFX.12408.1.S1_AT	proteasome (prosome, macropain) subunit, alpha type, 5		
GGA.915.1.S1_AT	suppressor of cytokine signaling 3		
GGA.1001.1.S1_AT	proteasome (prosome, macropain) subunit, alpha type, 6		
GGAAFFX.12055.1.S1_AT	ATG7 autophagy related 7 homolog (S. cerevisiae)		
GGAAFFX.3074.1.S1_S_AT	ATG7 autophagy related 7 homolog (S. cerevisiae)		
GGAAFFX.12150.1.S1_AT	ubiquitin-conjugating enzyme E2L 3		
GGA.9799.2.S1_S_AT	chromosome 4 open reading frame 20		
GGAAFFX.26407.3.S1_S_AT	cullin 3		

GGA.1883.1.S1_AT	ubiquitin specific peptidase 15
GGAAFFX.11588.1.S1_S_AT	S-phase kinase-associated protein 2 (p45)
GGA.2045.2.S1_A_AT	proteasome (prosome, macropain) subunit, alpha type, 7
GGA.4470.1.S1_AT	ubiquitin-conjugating enzyme E2N (UBC13 homolog, yeast)
GGA.2832.1.S1_AT	ubiquitin-conjugating enzyme E2N (UBC13 homolog, yeast)
GGA.1017.1.S1_S_AT	DET1 and DDB1 associated 1
GGA.20004.1.S1_S_AT	DET1 and DDB1 associated 1
GGAAFFX.12373.1.S1_AT	DET1 and DDB1 associated 1
GGAAFFX.12210.1.S1_S_AT	SUMO1 activating enzyme subunit 2
GGA.2381.1.S1_AT	similar to Putative ubiquitin-conjugating enzyme E2 D3-like protein
GGA.12733.1.S1_A_AT	ubiquitin specific peptidase 40
GGAAFFX.4436.1.S1_AT	cullin 2
GGA.7042.1.S1_A_AT	cullin 2
GGAAFFX.24906.1.S1_AT	ubiquitin specific peptidase 9, X-linked
GGAAFFX.2584.1.S1_S_AT	anaphase promoting complex subunit 5
GGAAFFX.12184.1.S1_AT	anaphase promoting complex subunit 5
GGA.6715.1.S1_AT	ubiquitin specific peptidase 34
GGA.6715.1.S1_S_AT	ubiquitin specific peptidase 34
GGAAFFX.26215.1.S1_S_AT	ubiquitin specific peptidase 34
GGAAFFX.23065.1.S1_S_AT	ubiquitin specific peptidase 38
GGA.16354.1.S1_S_AT	ATG4 autophagy related 4 homolog B (S. cerevisiae)
GGA.16354.1.S2_S_AT	ATG4 autophagy related 4 homolog B (S. cerevisiae)
GGA.5543.1.S1_S_AT	huntingtin interacting protein 2
GGA.5543.2.S1_AT	huntingtin interacting protein 2
GGA.10028.1.S1_AT	ubiquitin protein ligase E3C
GGAAFFX.11392.1.S1_S_AT	ubiquitin protein ligase E3C
GGAAFFX.12096.1.S1_S_AT	DCN1, defective in cullin neddylation 1, domain containing 1 (S. cerevisiae)

**Enrichment Score: 5.229145547043784**	**GOTERM_BP_FAT: 0006396~RNA processing**	**Count: 47**	***p* = 5.76E-08**
GGA.4811.3.S1_S_AT	heterogeneous nuclear ribonucleoprotein K		
GGA.4316.2.S1_A_AT	poly(A) polymerase alpha		
GGAAFFX.13197.1.S1_S_AT	poly(A) polymerase alpha		
GGAAFFX.11836.1.S1_S_AT	WD repeats and SOF1 domain containing; similar to WD repeats and SOF1 domain containing		
GGAAFFX.11221.1.S1_S_AT	WD repeats and SOF1 domain containing; similar to WD repeats and SOF1 domain containing		
GGAAFFX.11836.1.S1_AT	WD repeats and SOF1 domain containing; similar to WD repeats and SOF1 domain containing		
GGAAFFX.12997.1.S1_S_AT	mitochondrial translation optimization 1 homolog (S. cerevisiae)		
GGAAFFX.10146.1.S1_S_AT	mitochondrial translation optimization 1 homolog (S. cerevisiae)		
GGAAFFX.5884.2.S1_AT	RNA binding motif protein 25		
GGAAFFX.5884.1.S1_S_AT	RNA binding motif protein 25		
GGAAFFX.6371.2.S1_S_AT	integrator complex subunit 10		
GGA.4930.3.S1_A_AT	similar to KIAA0332		
GGAAFFX.20095.1.S1_AT	exosome component 9		
GGAAFFX.11671.1.S1_S_AT	RNA binding motif protein 22		
GGA.4751.1.S1_AT	RNA binding motif protein 22		
GGAAFFX.26094.1.S1_AT	La ribonucleoprotein domain family, member 6		
GGA.18371.1.S1_S_AT	La ribonucleoprotein domain family, member 6		
GGAAFFX.5692.4.S1_S_AT	CWC22 spliceosome-associated protein homolog (S. cerevisiae)		
GGAAFFX.5692.3.S1_S_AT	CWC22 spliceosome-associated protein homolog (S. cerevisiae)		
GGAAFFX.5692.1.S1_S_AT	CWC22 spliceosome-associated protein homolog (S. cerevisiae)		
GGA.9585.1.S1_S_AT	processing of precursor 4, ribonuclease P/MRP subunit (S. cerevisiae)		
GGA.9585.1.S1_A_AT	processing of precursor 4, ribonuclease P/MRP subunit (S. cerevisiae)		
GGA.2011.2.S1_S_AT	CLP1, cleavage and polyadenylation factor I subunit, homolog (S. cerevisiae)		
GGA.7783.1.S1_S_AT	survival of motor neuron protein interacting protein 1		
GGAAFFX.12391.1.S1_S_AT	nuclear cap binding protein subunit 1, 80kDa		
GGA.7069.1.S1_AT	serine/threonine kinase receptor associated protein		
GGA.2286.1.S1_AT	exosome component 10		
GGAAFFX.12036.1.S1_S_AT	three prime histone mRNA exonuclease 1		
GGAAFFX.11554.1.S1_AT	transducin (beta)-like 3		
GGA.5513.1.S1_A_AT	nucleolar protein 1, 120kDa		
GGAAFFX.12824.1.S1_AT	ribonuclease III, nuclear		
GGA.1146.1.S1_S_AT	Sjogren syndrome antigen B (autoantigen La)		
GGAAFFX.12116.1.S1_AT	Sjogren syndrome antigen B (autoantigen La)		
GGAAFFX.8604.1.S1_AT	UTP15, U3 small nucleolar ribonucleoprotein, homolog (S. cerevisiae)		
GGAAFFX.12917.1.S1_S_AT	cleavage and polyadenylation specific factor 3-like		
GGA.9367.2.S1_S_AT	tudor domain containing 3		
GGAAFFX.12338.1.S1_S_AT	tuftelin interacting protein 11		
GGAAFFX.11261.1.S1_AT	ribosomal RNA processing 1 homolog B (S. cerevisiae); similar to similar to Protein KIAA0179; hypothetical LOC426975		

GGA.3961.1.S1_AT	survival motor neuron
GGA.3961.1.S2_S_AT	survival motor neuron
GGA.6351.1.S1_AT	ribosomal L1 domain containing 1
GGA.9742.1.S1_S_AT	splicing factor 3a, subunit 1, 120kDa
GGAAFFX.21456.1.S1_AT	nucleolar protein family A, member 1 (H/ACA small nucleolar RNPs)
GGAAFFX.26050.1.S1_S_AT	M-phase phosphoprotein 10 (U3 small nucleolar ribonucleoprotein)
GGA.2835.1.S1_A_AT	WW domain binding protein 4 (formin binding protein 21)
GGAAFFX.12843.1.S1_AT	5'-3' exoribonuclease 2
GGAAFFX.11591.1.S1_S_AT	queuine tRNA-ribosyltransferase domain containing 1
GGAAFFX.26467.1.S1_AT	coiled-coil domain containing 76
GGAAFFX.11733.1.S1_S_AT	NOL1/NOP2/Sun domain family, member 2
GGAAFFX.22607.1.S1_S_AT	heterogeneous nuclear ribonucleoprotein L-like
GGAAFFX.13164.1.S1_AT	similar to nuclear DNA-binding protein; small unique nuclear receptor corepressor; C1D DNA-binding protein
GGA.5517.1.S1_S_AT	similar to nuclear DNA-binding protein; small unique nuclear receptor corepressor; C1D DNA-binding protein
GGAAFFX.23153.1.S1_S_AT	coiled-coil domain containing 131
GGA.17024.1.S1_AT	coiled-coil domain containing 131
GGA.9536.1.S1_S_AT	coiled-coil domain containing 131
GGAAFFX.13113.1.S1_AT	WD repeat domain 36
GGA.15911.1.S1_S_AT	WD repeat domain 36
GGA.1447.3.S1_S_AT	integrator complex subunit 2
GGAAFFX.12093.1.S1_S_AT	integrator complex subunit 2
GGA.5501.2.S1_A_AT	dihydrouridine synthase 1-like (S. cerevisiae)
GGAAFFX.12349.1.S1_AT	WD repeat domain 3; similar to WD repeat domain 3
GGAAFFX.525.3.S1_S_AT	protein arginine methyltransferase 7
GGAAFFX.12660.1.S1_S_AT	similar to nuclear receptor coactivator 6 interacting protein; trimethylguanosine synthase homolog (S. cerevisiae)
GGAAFFX.7445.1.S1_AT	cleavage stimulation factor, 3' pre-RNA, subunit 3, 77kDa

Supplementary Table 3. List of genes expressed during formation of collagen-gel based TECs.

Total probe sets with significant ≥2-fold change from 2D to collagen gel (restrained) = 694 (q < 0.01)

Top annotation clusters upregulated from 2D to collagen gel (restrained) (from 392 probe sets)

**Enrichment Score: 4.293716661032693**	**SP_PIR_KEYWORDS: disulfide bond**	**Count: 24**	***p* = 6.3463E-07**
GGA.3332.1.S1_AT	matrix-remodelling associated 8		
GGA.2961.1.S1_AT	gremlin 1, cysteine knot superfamily, homolog (Xenopus laevis)		
GGA.701.1.S1_AT	leukocyte ribonuclease A-1; leukocyte ribonuclease A-2		
GGA.701.1.S1_S_AT	leukocyte ribonuclease A-1; leukocyte ribonuclease A-2		
GGA.520.1.S1_AT	activin beta B		
GGA.520.2.S1_A_AT	activin beta B		
GGA.10960.1.S1_AT	collectin sub-family member 12		
GGA.15998.1.S1_AT	collectin sub-family member 12		
GGA.4345.1.S1_AT	secreted frizzled-related protein 2		
GGA.5058.1.S1_S_AT	cystatin C		
GGA.2679.1.S1_AT	fibulin 1		
GGA.1784.1.S1_AT	integrin, alpha 8		
GGA.1784.1.S2_AT	integrin, alpha 8		
GGA.1906.1.S2_AT	cysteine-rich secretory protein LCCL domain containing 1		
GGA.1907.1.S1_AT	platelet derived growth factor C		
GGA.3477.1.S1_AT	fibroblast growth factor receptor 2		
GGA.3477.1.S2_AT	fibroblast growth factor receptor 2		
GGA.663.1.S1_AT	purinergic receptor P2Y, G-protein coupled, 5		
GGA.5002.1.S1_AT	midkine (neurite growth-promoting factor 2)		
GGA.13901.1.S1_AT	similar to collagen XIV; collagen, type XIV, alpha 1 (undulin); similar to collagen, type XIV, alpha 1 (undulin)		
GGA.2558.1.S1_A_AT	similar to collagen XIV; collagen, type XIV, alpha 1 (undulin); similar to collagen, type XIV, alpha 1 (undulin)		
GGA.2558.3.S1_A_AT	similar to collagen XIV; collagen, type XIV, alpha 1 (undulin); similar to collagen, type XIV, alpha 1 (undulin)		
GGA.3977.3.S1_A_AT	aggrecan		
GGA.759.1.S1_AT	insulin-like growth factor binding protein 2, 36kDa		
GGA.1148.1.S2_AT	ST6 beta-galactosamide alpha-2,6-sialyltranferase 1		
GGA.1479.2.S1_A_AT	pleiotrophin		
GGA.1479.1.S1_AT	pleiotrophin		
GGA.483.1.S1_AT	cathepsin K		
GGA.635.1.S1_AT	glutaredoxin (thioltransferase)		
GGAAFFX.22381.3.S1_S_AT	ankyrin repeat and kinase domain containing 1		
GGA.817.1.S1_AT	plasminogen activator, urokinase		
GGA.4194.1.S1_AT	osteoglycin		

**Enrichment Score: 3.288419638810808**	**GOTERM_CC_FAT: 0005576~extracellular region**	**Count: 31**	***p* = 3.76E-08**
GGAAFFX.24566.1.S1_AT	laminin, alpha 4		
GGA.2625.1.S1_AT	retinoic acid receptor responder (tazarotene induced) 1		
GGA.12800.1.S1_AT	matrix metallopeptidase 3 (stromelysin 1, progelatinase)		
GGAAFFX.3200.1.S1_S_AT	fibulin 2		
GGA.17488.1.S1_AT	fibulin 2		
GGA.12454.1.S1_AT	relaxin 3		
GGA.3037.1.S1_S_AT	chordin-like 1		
GGA.1906.1.S2_AT	cysteine-rich secretory protein LCCL domain containing 1		
GGA.1907.1.S1_AT	platelet derived growth factor C		
GGA.398.1.S1_AT	matrix metalloproteinase-13		
GGA.5002.1.S1_AT	midkine (neurite growth-promoting factor 2)		
GGA.2558.1.S1_A_AT	similar to collagen XIV; collagen, type XIV, alpha 1 (undulin); similar to collagen, type XIV, alpha 1 (undulin)		
GGA.2558.3.S1_A_AT	similar to collagen XIV; collagen, type XIV, alpha 1 (undulin); similar to collagen, type XIV, alpha 1 (undulin)		
GGA.13901.1.S1_AT	similar to collagen XIV; collagen, type XIV, alpha 1 (undulin); similar to collagen, type XIV, alpha 1 (undulin)		
GGA.759.1.S1_AT	insulin-like growth factor binding protein 2, 36kDa		
GGA.3977.3.S1_A_AT	aggrecan		
GGA.1148.1.S2_AT	ST6 beta-galactosamide alpha-2,6-sialyltranferase 1		
GGA.1479.1.S1_AT	pleiotrophin		
GGA.1479.2.S1_A_AT	pleiotrophin		
GGA.2961.1.S1_AT	gremlin 1, cysteine knot superfamily, homolog (Xenopus laevis)		

GGAAFFX.26432.1.S1_S_AT	collagen, type XI, alpha 1		
GGA.690.1.S1_AT	lymphocyte antigen 86		
GGA.520.1.S1_AT	activin beta B		
GGA.520.2.S1_A_AT	activin beta B		
GGA.12006.1.S1_AT	adrenomedullin		
GGA.701.1.S1_S_AT	leukocyte ribonuclease A-1; leukocyte ribonuclease A-2		
GGA.701.1.S1_AT	leukocyte ribonuclease A-1; leukocyte ribonuclease A-2		
GGA.4345.1.S1_AT	secreted frizzled-related protein 2		
GGA.5058.1.S1_S_AT	cystatin C		
GGAAFFX.1236.1.S1_AT	matrix metallopeptidase 24 (membrane-inserted)		
GGA.2679.1.S1_AT	fibulin 1		
GGAAFFX.1557.1.S1_AT	glypican 1; similar to Glypican-1 precursor (Heparan sulfate proteoglycan core protein)		
GGA.4328.1.S1_AT	insulin-like growth factor binding protein 7		
GGAAFFX.2804.1.S1_S_AT	tenascin N		
GGA.817.1.S1_AT	plasminogen activator, urokinase		
GGA.11317.1.S1_AT	fibroblast growth factor 7 (keratinocyte growth factor)		
GGA.11317.2.S1_A_AT	fibroblast growth factor 7 (keratinocyte growth factor)		
GGA.4194.1.S1_AT	osteoglycin		

**Enrichment Score: 2.0064241**	**GOTERM_BP_FAT: 0030198~extracellular matrix organization**	**Count: 5**	***p* = 0.00507561**
GGAAFFX.26432.1.S1_S_AT	collagen, type XI, alpha 1		
GGA.3225.1.S1_AT	myosin, heavy chain 10, non-muscle; myosin, heavy chain 11, smooth muscle; similar to Myosin-11 (Myosin heavy chain, gizzard smooth muscle); similar to myosin, heavy chain 9, non-muscle		
GGA.3977.3.S1_A_AT	aggrecan		
GGA.2679.1.S1_AT	fibulin 1		
GGA.1784.1.S1_AT	integrin, alpha 8		
GGA.1784.1.S2_AT	integrin, alpha 8		

Top annotation clusters downregulated from 2D to collagen gel (restrained) (from 302 probe sets)

**Enrichment Score: 1.865281654990913**	**GOTERM_MF_FAT: 0005524~ATP binding**	**Count: 26**	***p* = 0.00480784**
GGA.4522.2.S1_A_AT	DEAH (Asp-Glu-Ala-His) box polypeptide 15; hypothetical LOC425141; similar to putative RNA helicase and RNA dependent ATPase		
GGA.1056.2.S1_S_AT	hypothetical LOC426502; Obg-like ATPase 1; similar to GTP-binding protein 9 (putative)		
GGAAFFX.2596.3.S1_S_AT	suppressor of var1, 3-like 1 (S. cerevisiae)		
GGAAFFX.6405.2.S1_S_AT	heat shock 70kDa protein 4-like		
GGAAFFX.26040.1.S1_S_AT	excision repair cross-complementing rodent repair deficiency, complementation group 6-like		
GGAAFFX.26684.1.S1_AT	similar to PAK3 protein; p21 (CDKN1A)-activated kinase 3; p21 protein (Cdc42/Rac)-activated kinase 1		
GGAAFFX.11524.1.S1_S_AT	chaperonin containing TCP1, subunit 4 (delta)		
GGAAFFX.12224.1.S1_AT	DNA2 DNA replication helicase 2-like (yeast)		
GGAAFFX.2478.1.S1_AT	threonyl-tRNA synthetase-like 2		
GGAAFFX.10854.1.S1_AT	hypothetical LOC428066		
GGAAFFX.9739.1.S1_S_AT	RIO kinase 2 (yeast)		
GGAAFFX.7711.1.S1_AT	kinesin family member 18A		
GGA.11797.1.S1_S_AT	vaccinia related kinase 1		
GGA.5687.4.S1_A_AT	kinesin family member 2C		
GGA.5687.2.S1_A_AT	kinesin family member 2C		
GGA.6269.2.S1_S_AT	phenylalanyl-tRNA synthetase, beta subunit		
GGAAFFX.12676.1.S1_S_AT	phenylalanyl-tRNA synthetase, beta subunit		
GGAAFFX.644.2.S1_S_AT	mitogen-activated protein kinase kinase 4		
GGA.12171.1.S1_AT	DEAD (Asp-Glu-Ala-Asp) box polypeptide 24		
GGA.12171.1.S1_S_AT	DEAD (Asp-Glu-Ala-Asp) box polypeptide 24		
GGA.10727.1.S1_S_AT	3'-phosphoadenosine 5'-phosphosulfate synthase 1		
GGAAFFX.10108.1.S1_AT	TTK protein kinase		
GGA.115.1.S1_AT	RecQ protein-like (DNA helicase Q1-like)		
GGAAFFX.11562.1.S1_S_AT	origin recognition complex, subunit 1-like (yeast)		
GGAAFFX.21290.1.S1_S_AT	DEAD (Asp-Glu-Ala-Asp) box polypeptide 50		
GGA.16459.1.S1_S_AT	DEAD (Asp-Glu-Ala-Asp) box polypeptide 50		
GGAAFFX.3712.1.S1_S_AT	hypothetical protein LOC776374; dihydroxyacetone kinase 2 homolog (S. cerevisiae)		
GGAAFFX.7514.1.S1_AT	spermatogenesis associated 5		
GGAAFFX.3447.1.S1_S_AT	DEAD (Asp-Glu-Ala-Asp) box polypeptide 52		
GGAAFFX.10518.1.S1_S_AT	maternal embryonic leucine zipper kinase		

**Enrichment Score: 1.649835646167675**	**GOTERM_MF_FAT: 0008026~ATP-dependent helicase activity**	**Count: 6**	***p* = 0.00346133**
GGA.12171.1.S1_S_AT	DEAD (Asp-Glu-Ala-Asp) box polypeptide 24		
GGA.12171.1.S1_AT	DEAD (Asp-Glu-Ala-Asp) box polypeptide 24		

GGAAFFX.12224.1.S1_AT	DNA2 DNA replication helicase 2-like (yeast)
GGA.4522.2.S1_A_AT	DEAH (Asp-Glu-Ala-His) box polypeptide 15; hypothetical LOC425141; similar to putative RNA helicase and RNA dependent ATPase
GGA.115.1.S1_AT	RecQ protein-like (DNA helicase Q1-like)
GGAAFFX.21290.1.S1_S_AT	DEAD (Asp-Glu-Ala-Asp) box polypeptide 50
GGA.16459.1.S1_S_AT	DEAD (Asp-Glu-Ala-Asp) box polypeptide 50
GGAAFFX.3447.1.S1_S_AT	DEAD (Asp-Glu-Ala-Asp) box polypeptide 52

Total probe sets with significant ≥2-fold change from collagen gel (restrained) to 3D collagen TEC = 234 (q <

Top annotation cluster upregulated from collagen gel (restrained) to 3D collagen TEC (from 43 probe sets)

**Enrichment Score: 3.0348234776087404**	**UP_SEQ_FEATRE: disulfide bond**	**Count: 24**	***p* = 3.54E-06**
GGA.540.1.S1_AT	matrix Gla protein		
GGA.595.1.S1_AT	NEL-like 2 (chicken)		
GGA.701.1.S1_S_AT	leukocyte ribonuclease A-1; leukocyte ribonuclease A-2		
GGA.701.1.S1_AT	leukocyte ribonuclease A-1; leukocyte ribonuclease A-2		
GGA.10960.1.S1_AT	collectin sub-family member 12		
GGA.15998.1.S1_AT	collectin sub-family member 12		
GGA.514.1.S1_AT	similar to Coagulation factor X precursor (Stuart factor) (Virus-activating protease) (VAP); coagulation factor X		
GGA.481.1.S1_AT	fibromodulin		
GGA.4723.1.S1_AT	leukocyte cell derived chemotaxin 1		
GGA.870.2.S1_A_AT	fibroblast growth factor receptor 1		
GGAAFFX.21790.1.S1_S_AT	gap junction protein, alpha 1, 43kDa		
GGA.1987.1.S2_AT	neuronal cell adhesion molecule		
GGA.1987.1.S1_A_AT	neuronal cell adhesion molecule		
GGA.663.1.S1_AT	purinergic receptor P2Y, G-protein coupled, 5		
GGA.544.1.S1_S_AT	purinergic receptor P2Y, G-protein coupled, 5		
GGA.652.1.S1_AT	arginine vasopressin (neurophysin II, antidiuretic hormone, diabetes insipidus, neurohypophyseal)		
GGA.1479.2.S1_A_AT	pleiotrophin		
GGA.1479.1.S1_AT	pleiotrophin		
GGA.1148.1.S1_AT	ST6 beta-galactosamide alpha-2,6-sialyltranferase 1		
GGA.1148.1.S2_AT	ST6 beta-galactosamide alpha-2,6-sialyltranferase 1		
GGA.2551.2.S1_A_AT	lactotransferrin		
GGA.239.1.S1_AT	tolloid-like 1		
GGA.817.1.S1_AT	plasminogen activator, urokinase		
GGA.9772.1.S1_S_AT	fibronectin 1		
GGA.13487.1.A1_AT	fibronectin 1		
GGA.739.1.S1_AT	quiescence-specific protein		
GGA.4194.1.S1_AT	osteoglycin		

Top annotation clusters downregulated from collagen gel (restrained) to 3D collagen TEC (from 191 probe sets)

**Enrichment Score: 3.8738426346541743**	**GOTERM_BP_FAT: 0006260~DNA replication**	**Count: 9**	***p* = 1.42E-05**
GGAAFFX.11455.1.S1_S_AT	minichromosome maintenance complex component 3		
GGA.8862.1.S1_S_AT	chromatin assembly factor 1, subunit B (p60)		
GGAAFFX.12177.1.S1_S_AT	minichromosome maintenance complex component 5		
GGAAFFX.12419.1.S1_S_AT	denticleless homolog (Drosophila)		
GGAAFFX.11513.1.S1_AT	cyclin E2		
GGAAFFX.11696.1.S1_S_AT	minichromosome maintenance complex component 2		
GGAAFFX.12275.1.S1_S_AT	minichromosome maintenance complex component 6		
GGA.4514.2.S1_S_AT	ribonucleotide reductase M1 polypeptide		
GGA.7164.1.S1_AT	similar to cdc21p; similar to DNA replication initiator protein; minichromosome maintenance complex component 4		

**Enrichment Score: 3.046203999852737**	**GOTERM_BP_FAT: 0008610~lipid biosynthetic process**	**Count: 7**	***p* = 6.38E-04**
GGA.8817.1.S1_S_AT	farnesyl-diphosphate farnesyltransferase 1		
GGAAFFX.12935.1.S1_S_AT	24-dehydrocholesterol reductase		
GGAAFFX.9274.1.S1_AT	dimethylallyltranstransferase, geranyltranstransferase)		
GGAAFFX.12964.1.S1_S_AT	lanosterol synthase (2,3-oxidosqualene-lanosterol cyclase)		
GGAAFFX.3863.1.S1_S_AT	lanosterol synthase (2,3-oxidosqualene-lanosterol cyclase)		
GGAAFFX.12469.1.S1_S_AT	ELOVL family member 6, elongation of long chain fatty acids (FEN1/Elo2, SUR4/Elo3-like, yeast)		

GGAAFFX.12469.1.S1_AT	ELOVL family member 6, elongation of long chain fatty acids (FEN1/Elo2, SUR4/Elo3-like, yeast)
GGA.8851.2.S1_A_AT	isopentenyl-diphosphate delta isomerase 1
GGAAFFX.21726.1.S1_AT	3-hydroxy-3-methylglutaryl-Coenzyme A reductase

Total probe sets with significant ≥2-fold change from 3D collagen TEC to mature collagen TEC = 58 (q < 0.01)

No significant annotation cluster upregulated from 3D collagen TEC to mature collagen TEC (from 58 probe sets)

Top annotation clusters downregulated from 3D collagen TEC to mature collagen TEC (from 918 probe sets)

**Enrichment Score: 7.392746274101243**	**GOTERM_CC_FAT: 0005856~cytoskeleton**	**Count: 37**	***p* = 2.85E-06**
GGAAFFX.5870.1.S1_AT	A kinase (PRKA) anchor protein (yotiao) 9		
GGAAFFX.11417.1.S1_AT	kinesin family member 11; hypothetical LOC426105; similar to similar to kinesin like protein		
GGAAFFX.4223.1.S1_S_AT	kinesin family member 11; hypothetical LOC426105; similar to similar to kinesin like protein		
GGA.1297.1.S1_AT	lamin B2		
GGAAFFX.22491.2.S1_S_AT	spectrin, beta, non-erythrocytic 1		
GGAAFFX.22491.4.S1_S_AT	spectrin, beta, non-erythrocytic 1		
GGA.4547.2.S1_A_AT	myosin, heavy chain 10, non-muscle; myosin, heavy chain 11, smooth muscle; similar to Myosin-11 (Myosin heavy chain, gizzard smooth muscle); similar to myosin, heavy chain 9, non-muscle		
GGAAFFX.12587.1.S1_S_AT	cytoskeleton associated protein 2		
GGAAFFX.5404.1.S1_S_AT	nucleolar and spindle associated protein 1		
GGA.17943.1.S1_AT	Rho-associated, coiled-coil containing protein kinase 2		
GGA.3500.1.S1_AT	Rho-associated, coiled-coil containing protein kinase 2		
GGAAFFX.11794.1.S1_S_AT	capping protein (actin filament) muscle Z-line, alpha 1		
GGA.9459.1.S1_S_AT	NUF2, NDC80 kinetochore complex component, homolog (S. cerevisiae)		
GGAAFFX.13010.1.S1_AT	NUF2, NDC80 kinetochore complex component, homolog (S. cerevisiae)		
GGA.1368.1.S1_AT	lamin B1		
GGA.1368.1.S2_AT	lamin B1		
GGAAFFX.12208.1.S1_S_AT	kinesin family member 20A		
GGAAFFX.11455.1.S1_S_AT	minichromosome maintenance complex component 3		
GGAAFFX.12873.1.S1_AT	spermatid perinuclear RNA binding protein		
GGA.3130.2.S1_A_AT	CAP-GLY domain containing linker protein 1		
GGA.3957.2.S1_A_AT	enabled homolog (Drosophila)		
GGAAFFX.24513.7.S1_S_AT	(Differentially expressed in adenocarcinoma of the lung protein 1) (DAL-1)		
GGAAFFX.2572.1.S1_AT	myosin IE		
GGA.388.1.S1_AT	similar to beta-keratin related protein; beta-keratin related protein		
GGA.388.2.S1_A_AT	similar to beta-keratin related protein; beta-keratin related protein		
GGAAFFX.12418.1.S1_S_AT	pericentrin		
GGAAFFX.13032.1.S1_S_AT	kinesin family member 23		
GGA.2375.1.S1_AT	catenin (cadherin-associated protein), alpha 2		
GGAAFFX.6135.1.S1_AT	centromere protein F, 350/400ka (mitosin)		
GGAAFFX.6125.1.S1_AT	centromere protein F, 350/400ka (mitosin)		
GGA.3705.1.S1_AT	centromere protein F, 350/400ka (mitosin)		
GGAAFFX.20508.1.S1_S_AT	centromere protein F, 350/400ka (mitosin)		
GGAAFFX.12568.1.S1_AT	coiled-coil domain containing 5 (spindle associated)		
GGAAFFX.1153.1.S1_S_AT	coiled-coil domain containing 5 (spindle associated)		
GGAAFFX.9982.4.S1_S_AT	adducin 1 (alpha)		
GGA.14450.1.S1_AT	ADP-ribosylation factor-like 8B; ADP-ribosylation factor-like 8A		
GGA.13442.1.S2_S_AT	radixin		
GGA.13442.1.S1_AT	radixin		
GGA.1551.2.S1_A_AT	baculoviral IAP repeat-containing 5 (survivin)		
GGAAFFX.24061.2.S1_S_AT	similar to myosin X; myosin X		
GGA.698.1.S2_AT	vinculin		
GGAAFFX.11749.1.S1_AT	NDC80 homolog, kinetochore complex component (S. cerevisiae)		
GGA.9350.1.S1_S_AT	NDC80 homolog, kinetochore complex component (S. cerevisiae)		
GGAAFFX.8944.1.S1_S_AT	serine/threonine kinase 38 like		
GGAAFFX.8944.1.S1_AT	serine/threonine kinase 38 like		
GGA.3146.1.S1_AT	cyclin B2		
GGA.2844.2.S1_S_AT	stathmin 1		
GGA.4358.1.S1_AT	kinesin family member 4A		
GGA.4358.2.S1_A_AT	kinesin family member 4A		
GGA.4358.1.S1_A_AT	kinesin family member 4A		
GGA.2129.1.S1_S_AT	microtubule-associated protein 1B		
GGA.18411.1.S1_AT	microtubule-associated protein 1B		
GGAAFFX.11402.1.S1_AT	NIMA (never in mitosis gene a)-related kinase 2		

Enrichment Score: 6.608049695121445	GOTERM_BP_FAT: 0000087~M phase of mitotic cell cycle	Count: 13	*p* = 3.78E-08
GGAAFFX.4223.1.S1_S_AT	kinesin family member 11; hypothetical LOC426105; similar to similar to kinesin like protein		
GGAAFFX.11417.1.S1_AT	kinesin family member 11; hypothetical LOC426105; similar to similar to kinesin like protein		
GGA.4066.1.S1_AT	structural maintenance of chromosomes 2		
GGAAFFX.5404.1.S1_S_AT	nucleolar and spindle associated protein 1		
GGAAFFX.6135.1.S1_AT	centromere protein F, 350/400ka (mitosin)		
GGAAFFX.20508.1.S1_S_AT	centromere protein F, 350/400ka (mitosin)		
GGAAFFX.6125.1.S1_AT	centromere protein F, 350/400ka (mitosin)		
GGA.3705.1.S1_AT	centromere protein F, 350/400ka (mitosin)		
GGAAFFX.13010.1.S1_AT	NUF2, NDC80 kinetochore complex component, homolog (S. cerevisiae)		
GGA.9459.1.S1_S_AT	NUF2, NDC80 kinetochore complex component, homolog (S. cerevisiae)		
GGA.8462.1.S1_AT	BUB1 budding uninhibited by benzimidazoles 1 homolog beta (yeast)		
GGAAFFX.24391.1.S1_AT	PDS5, regulator of cohesion maintenance, homolog A (S. cerevisiae)		
GGA.9350.1.S1_S_AT	NDC80 homolog, kinetochore complex component (S. cerevisiae)		
GGAAFFX.11749.1.S1_AT	NDC80 homolog, kinetochore complex component (S. cerevisiae)		
GGA.3146.1.S1_AT	cyclin B2		
GGA.5793.1.S1_AT	similar to Brrn1-prov protein		
GGA.726.2.S1_A_AT	cell division cycle 2, G1 to S and G2 to M		
GGA.4129.1.S1_AT	cyclin A2		
GGAAFFX.11402.1.S1_AT	NIMA (never in mitosis gene a)-related kinase 2		

Enrichment Score: 5.992696601471587	GOTERM_CC_FAT: 0000793~condensed chromosome	Count: 13	*p* = 1.34E-10
GGA.4493.1.S1_AT	breast cancer 1, early onset		
GGA.4493.1.S2_AT	breast cancer 1, early onset		
GGA.12212.1.S1_A_AT	centromere protein K		
GGAAFFX.6135.1.S1_AT	centromere protein F, 350/400ka (mitosin)		
GGA.3705.1.S1_AT	centromere protein F, 350/400ka (mitosin)		
GGAAFFX.20508.1.S1_S_AT	centromere protein F, 350/400ka (mitosin)		
GGAAFFX.6125.1.S1_AT	centromere protein F, 350/400ka (mitosin)		
GGA.9459.1.S1_S_AT	NUF2, NDC80 kinetochore complex component, homolog (S. cerevisiae)		
GGAAFFX.13010.1.S1_AT	NUF2, NDC80 kinetochore complex component, homolog (S. cerevisiae)		
GGA.8462.1.S1_AT	BUB1 budding uninhibited by benzimidazoles 1 homolog beta (yeast)		
GGAAFFX.541.1.S1_S_AT	bromodomain adjacent to zinc finger domain, 1B		
GGA.9350.1.S1_S_AT	NDC80 homolog, kinetochore complex component (S. cerevisiae)		
GGAAFFX.11749.1.S1_AT	NDC80 homolog, kinetochore complex component (S. cerevisiae)		
GGA.1914.1.S1_S_AT	Bloom syndrome		
GGA.944.1.S1_AT	centromere protein H		
GGA.2010.2.S1_A_AT	topoisomerase (DNA) II alpha 170kDa		
GGA.4061.2.S1_A_AT	high-mobility group box 2		
GGA.7180.1.S1_S_AT	BUB1 budding uninhibited by benzimidazoles 1 homolog (yeast)		
GGAAFFX.11402.1.S1_AT	NIMA (never in mitosis gene a)-related kinase 2		

Supplementary Table 4. List of common genes upregulated during tendon development and formation of 3D TECs.

Comparison	Common genes
Common in formation of 3D fibrin TECs and during E11 to E14 tendon development	*Agxt2l1, Ahnak2, Ano1, Cd121a, Cybrd1, Fn1, Gpx3, Kcne4, Mxra8*
Common in formation of 3D fibrin TECs and during E14 to E17 tendon development	*Appl2, Fam189a2, Fbxo32, Gfpt2, Pde4d, Sox9*
Common in formation of 3D fibrin TECs and during both E11 to E14 and E14 to E17 tendon development	*Ninj2*
Common in formation of 3D collagen TECs and during E11 to E14 tendon development	*Ch25h, Crem, Loc422305, Loc770126*
Common in formation of 3D collagen TECs and during E14 to E17 tendon development	*Angtl7, Comp, Ltf, Mfap3l, Prosapip1, Sod3, St6gal1, Vnn1*
Common in formation of 3D collagen TECs and during both E11 to E14 and E14 to E17 tendon development	*Apoa1, Mgp*
Common in maturation of 3D fibrin TECs and during E11 to E14 tendon development	*Agx2l1, Ahank2, Cd109, Cd81, Clec3b, Col10a1, Creg1, D10jhu81e, Ensgalg00000011687, Ensgalg00000014965, F13a1, Faah, Fabp5, Fam70b, Fgl1, Hlf, Hnmt, Itgbl1, Loc423474, Loc776810, Mfap5, Ndrg1, P4ha1, Pdn, Perp, Rora*
Common in maturation of 3D fibrin TECs and during E14 to E17 tendon development	*5ht1e, Angptl7, Avd, Blb1, C11orf52, C20orf82, CBPch04, Chia, Comp, Eml1, Entpd1, Fam13a1, Fkbp5, Fker, Gal1, Hapln1, Kcnj15, Lama3, Lama5, Lgals3, Loc776860, Lonrf2, Ltf, Ly75, Lyg2, Mfap3l, Mbp, Pcolce2, Serpinf2, Sesn1, Slc13a4, Slc22a16, Sod3, Sox9, Sult1e1, Vnn1, Zbtb16, Zbtb20*
Common in maturation of 3D fibrin TECs and during both E11 to E14 and E14 to E17 tendon development	*Angptl5, Bcl2, Comp, Cpm, Eln, Fabp4, As6, Gpsm1, Igsf10, Loc772003, Ly86, Matn2, Mfap5, Pdk4, Rasd1, Tnxb, Tsc22d3, Vipr2*
Common in maturation of 3D collagen TECs and during E11 to E14 tendon development	*Hbe1, Igfbp7, Kcne4*
Common in maturation of 3D collagen TECs and during E14 to E17 tendon development	*C5h11orf96*

Common in maturation of 3D collagen TECs and during both E11 to E14 and E14 to E17 tendon development	*Apoa1, Mgp*

Supplementary Table 5. List of IPA-generated pathways regulated during E11 to E14 and E14 to E17 of tendon development.

Canonical Pathway	E11 to E14	E14 to E17
ILK Signaling	1.414213562	-1.897366596
RhoA Signaling		-2.449489743
LXR/RXR Activation	0.447213595	1.632993162
Agrin Interactions at Neuromuscular Junction		-2
Complement System		1
Calcium Signaling	0.447213595	0.447213595
Acute Phase Response Signaling	0.447213595	
Nitric Oxide Signaling in the Cardiovascular System	-0.447213595	
Urea Cycle		
Cytotoxic T Lymphocyte-mediated Apoptosis of Target Cells		
Agranulocyte Adhesion and Diapedesis		
Cysteine Biosynthesis III (mammalia)		
Allograft Rejection Signaling		
Tight Junction Signaling		
Hepatic Fibrosis / Hepatic Stellate Cell Activation		
Inhibition of Angiogenesis by TSP1		
LPS/IL-1 Mediated Inhibition of RXR Function		
STAT3 Pathway		
Cholesterol Biosynthesis I		
RAR Activation		
Cholesterol Biosynthesis III (via Desmosterol)		
Mechanisms of Viral Exit from Host Cells		
Retinol Biosynthesis		
Crosstalk between Dendritic Cells and Natural Killer Cells		
Caveolar-mediated Endocytosis Signaling		
Triacylglycerol Degradation		
Superpathway of Citrulline Metabolism		
Threonine Degradation II		
Atherosclerosis Signaling		
Heparan Sulfate Biosynthesis		
Retinoate Biosynthesis I		
Ethanol Degradation II		
Thyroid Hormone Metabolism II (via Conjugation and/or Degradation)		
Superpathway of Methionine Degradation		
Granulocyte Adhesion and Diapedesis		
Histamine Degradation		
Thyronamine and Iodothyronamine Metabolism		
Glycine Degradation (Creatine Biosynthesis)		
Type II Diabetes Mellitus Signaling		
Thyroid Hormone Metabolism I (via Deiodination)		
Cholesterol Biosynthesis II (via 24,25-dihydrolanosterol)		
Unfolded protein response		
Epithelial Adherens Junction Signaling		
Actin Cytoskeleton Signaling		
Methionine Degradation I (to Homocysteine)		
Superpathway of Cholesterol Biosynthesis		
Glycine Betaine Degradation		
Cellular Effects of Sildenafil (Viagra)		
Circadian Rhythm Signaling		

Chondroitin Sulfate Biosynthesis		
Remodeling of Epithelial Adherens Junctions		
Heparan Sulfate Biosynthesis (Late Stages)		

Legends to supplementary figures, tables and movies.

Supplementary Figure 1. Quality control of the microarray samples.

Gel electrophoresis was used to analysed the quality of RNA samples collected from sequential steps of embryonic tendon development *in vivo* (**a**), fibrin-based TECs (**b**) and collagen-based TECs (**c**). (**d**) Box plots of normalised microarray readouts of all the samples. *Indicates sample was not suitable to use for further analyses.

Supplementary Figure 2. Clustering of genes differentially regulated during tendon development.

Stringent selection of genes differentially regulated (fold change ≥±2, q <0.05) between E11 and E14, E14 and E17, and E11 and E17 of tendon development in vivo produced 2090 probe sets. K-means clustering algorithm produced 8 clusters based on similarity of expression profile across E11-E17 (mean values log2 standardised). (**a**) The mean expression of probe sets in each cluster is plotted in blue. (**b**) Heat map representation of the 8 clusters and comparison with expression in CTFs in vitro. Blue is low expression, red is high expression. *Indicates clusters where gene expression profiles in TECs are similar to tendon development in vivo. (**c**) Clusters 3, 5 and 6 were examined by functional enrichment analysis and the top GO terms from each cluster is shown.

Supplementary Figure 3. PCA mapping based on differentially regulated genes during tendon development *in vivo*.

Principal component analysis (PCA), on the mean of each of the conditions in this study, using only the 2090 probe sets that were differentially regulated (fold change ≥±2, q <0.05) between E11 and E14, E14 and E17, and E11 and E17 of tendon development in vivo. The first principal component (PC #1) accounted for 55% of the gene expression variance in the samples and separated the embryonic tendons from the in vitro samples. PC #2 accounted for 18% of the variance and revealed that embryonic tendons at E17 were similar to CTFs in mature fibrin TECs.

Supplementary Figure 4. Calcium signalling is a pathway that is similarly regulated during the E11-E14 and E14-E17 transition of tendon development.

Genes that were differentially regulated during E11-E14 and E14-E17 of tendon development were clustered into canonical pathways using IPA. Similarly regulated pathways identified in both transition stages included upregulation of ‘calcium signalling’ in E11 to E14 (**a**) and E14 to E17 (**b**)

of tendon development. Red indicates upregulation and green indicates downregulation. Bold colours indicate high degree of regulation (>±2.5-folds), pale colours indicate ±2- to ±2.5-fold differential regulation, and non-coloured genes mean no significant regulation found.

Supplementary Figure 5. Pathways that are regulated differently during the E11-E14 and E14-E17 transition of tendon development.

Genes that were differentially regulated during E11-E14 and E14-E17 of tendon development were clustered into canonical pathways using IPA. Pathways that were regulated differently during E11 and E14 compared with during E14 and E17 of tendon development included ‘integrin-linked kinase signalling’, which was upregulated during E11 to E14 (**a**) but downregulated from E14 to E17 (**b**), and ‘RhoA signalling’ which was not differentially regulated from E11 to E14 (**c**) but is downregulated from E14 to E17 (**d**). Red indicates upregulation and green indicates downregulation. Bold colours indicate high degree of regulation (>±2.5-folds), pale colours indicates ±2- to ±2.5-fold differential regulation, and non-coloured genes mean no significant regulation found.

Supplementary Figure 6. CTFs in 3D fibrin-base TECs synthesise parallel-aligned collagen fibrils.

Electron microscopy images of transverse section across a 3D fibrin-based TEC showing cells surrounded by narrow diameter (~35 nm) collagen fibrils that were aligned parallel to the long axis of the TECs. Scale bar 3 μm.

Supplementary Figure 7. CTFs attach to collagen fibrils synthesise *de novo* in a pre-existing collagen scaffold in collagen-based TECs.

Electron microscopy images of transverse section across a 3D collagen-based TEC showing cells surrounded by pre-existing collagen scaffold and newly synthesised narrow-diameter collagen fibrils. Scale bar 3 μm.

Supplementary Figure 8. Expression of *Yap1* in tendons *in vivo* and CTFs in culture.

Expression levels of *Yap1*, detected by two probe sets, from the microarray data were normalised relative to the expression level in CTFs culture in 2D. Bars show SEM.

Supplementary Table 1. List of genes expressed during chick embryonic tendon development.

Supplementary Table 2. List of genes expressed during formation of fibrin-gel based TECs.

Supplementary Table 3. List of genes expressed during formation of collagen-gel based TECs.

Supplementary Table 4. List of common genes upregulated during tendon development and formation of 3D TECs.

Supplementary Table 5. List of IPA-generated pathways regulated during E11 to E14 and E14 to E17 of tendon development.

Supplementary Movie 1. Step-through movie of SBF-SEM of a 3D fibrin gel-based TEC cut, and imaged, transversely to the long axis of the TEC.

The long axis of the TEC is perpendicular to the EM image. Individual cells are depicted in different colours. The EM image that is superimposed on the 3D reconstruction is 40 µm x 40 µm in size.

Supplementary Movie 2. Step-through movie of SBF-SEM of a 3D collagen gel-based TEC cut, and imaged, transversely to the long axis of the TEC.

The long axis of the TEC is perpendicular to the EM image. Individual cells are depicted in different colours. The EM image that is superimposed on the 3D reconstruction is 40 µm x 40 µm in size.
